# Catalytic Activation of Imines by Chalcogen Bond Donors in a Povarov [4+2] Cycloaddition Reaction

**DOI:** 10.1002/chem.202200917

**Published:** 2022-07-12

**Authors:** Tim Steinke, Patrick Wonner, Richard M. Gauld, Sascha Heinrich, Stefan M. Huber

**Affiliations:** ^1^ Fakultät für Chemie und Biochemie Ruhr-Universität Bochum Universitätsstraße 150 44801 Bochum Germany; ^2^ Fakultät für Biologie und Biotechnologie Ruhr-Universität Bochum Universitätsstraße 150 44801 Bochum Germany

**Keywords:** chalcogen bonding, cycloaddition, Lewis acids, non-covalent interactions, organocatalysis

## Abstract

Recently, chalcogen bonding has been investigated in more detail in organocatalysis and the scope of activated functionalities continues to increase. Herein, the activation of imines in a Povarov [4+2] cycloaddition reaction with bidentate cationic chalcogen bond donors is presented. Tellurium‐based Lewis acids show superior properties compared to selenium‐based catalysts and inactive sulfur‐based analogues. The catalytic activity of the chalcogen bonding donors increases with weaker binding anions. Triflate, however, is not suitable due to its participation in the catalytic pathway. A solvent screening revealed a more efficient activation in less polar solvents and a pronounced effect of solvent (and catalyst) on *endo : exo* diastereomeric ratio. Finally, new chiral chalcogen bonding catalysts were applied but provided only racemic mixtures of the product.

## Graphical abstract

**Tellurium‐based chalcogen** bonding catalysts successfully activate a Povarov [4+2] cycloaddition with catalyst loadings as low as 1 mol %, with different substrates, and in different solvents. Furthermore, new chiral chalcogen bonding catalysts were introduced and tested in this reaction, but only racemic mixtures of the product were obtained.

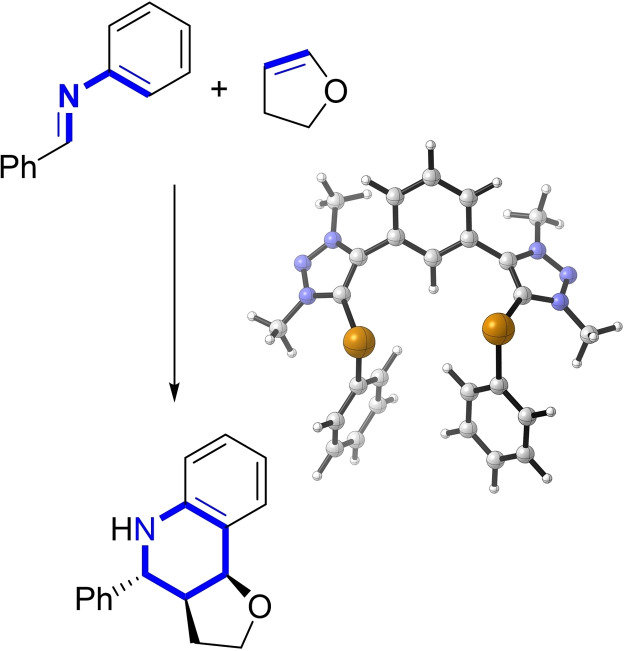

## Introduction

Non‐covalent interactions, such as hydrogen,^[1]^ halogen,^[2]^ and chalcogen bonding^[3]^ attract increasing interest in the field of organocatalysis. Whereas the first ones have been studied in more detail in the last decades,[^1^, ^4^] the latter is still on its way to be established in this field.

Chalcogen bonding can be defined as Lewis acidic chalcogen atoms interacting with Lewis bases, such as halides or nitrogen functionalities (like imines). The interaction is based on three electronic contributions: an n→σ* orbital interaction,^[5]^ electrostatic attraction to the region of positive potential in the elongation of the Ch−R bond (σ‐hole^[6]^), and dispersion.^[7]^ Compared to hydrogen bonding, chalcogen bonding is more tunable, as the chalcogen atom can be varied and the interaction strength be thus adjusted (Te>Se>S). Furthermore, the interactions angles are highly defined, which could be beneficial to achieve high selectivities.[^5b^, ^6b^, ^8^]

First applications of chalcogen bonding were described in solid state systems.[^5b^, ^9^] Especially the group of Gleiter performed pioneering work with quantum‐chemical studies[^7^, ^10^] and the construction of porous materials.^[11]^ The first applications in solution have been reported in the 1990s by Tomoda^[12]^ and Wirth,^[13]^ who used intramolecular selenium‐based chalcogen bonding to rigidify reagents towards stereoselective transformations.^[14]^ In the last decade, intermolecular interactions were introduced in anion recognition^[15]^ and transport.^[16]^ Subsequently, Matile et al. presented the first example of chalcogen bonding in organocatalysis in form of the reduction of quinolines by neutral sulfur‐based chalcogen bond donors.^[17]^ Our group then used cationic selenium‐based Lewis acids in carbon‐halogen bond activations,^[18]^ and in the reduction of quinolines.^[19]^ Other functional groups, such as carbonyl compounds^[20]^ and π‐systems^[21]^ were successfully targeted by Wang with selenium‐based chalcogen bond donors. Finally, tellurium as Lewis acidic center was also employed, and the corresponding catalysts were able to successfully activate nitro‐^[22]^ and carbonyl functionalities,^[23]^ as well as a carbon‐chloride bonds.^[24]^ Next to these tellurium(II) derivatives, recently higher oxidized telluronium cations have also been shown to be very potent organocatalysts.^[25]^

**Scheme 1:**
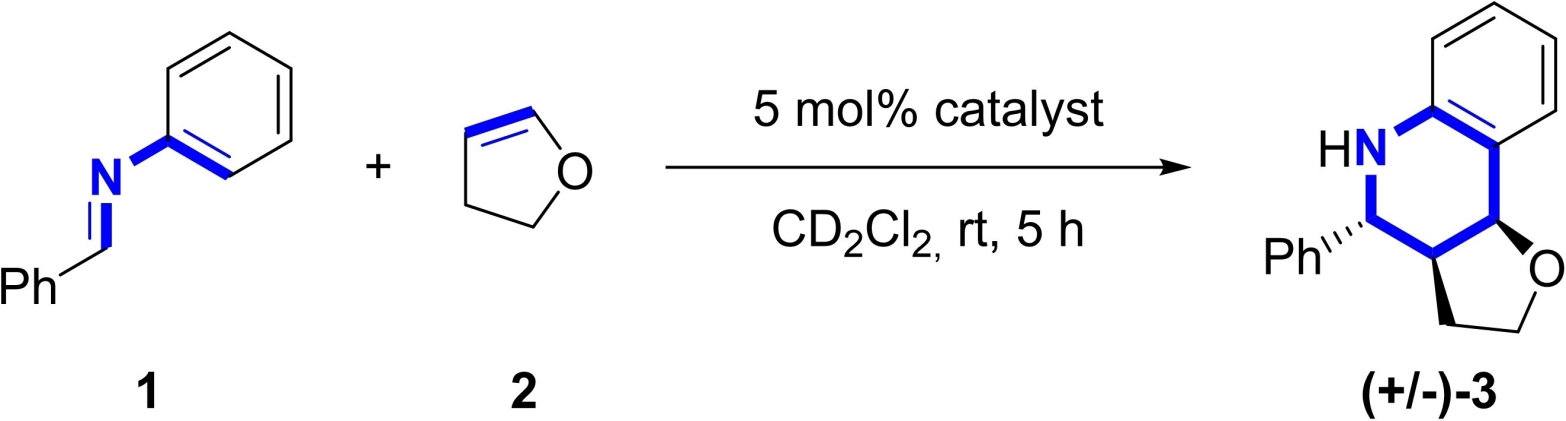
Povarov [4+2] cycloaddition reaction of *N*‐benzylideneaniline (**1**) with two equivalents of 2,3‐dihydrofuran (DHF) (**2**) in presence of 5 mol % of catalyst. For clarity reasons, only one diastereomer is shown.

An obvious next step in chalcogen‐based catalysis is the introduction of chiral catalyst for enantioselective transformations and therefore suitable test reactions are needed. As imines have already been successfully activated by chalcogen bonding,[^17^, ^19^, ^25a^] we decided to extend this approach to a Povarov [4+2] cycloaddition reaction (Scheme 1),^[26]^ which generates three new stereocenters and should thus constitute an ideal benchmark reaction for asymmetric induction.

Herein, we present the successful activation of *N*‐benzylidene‐aniline (**1**) and derivatives in this Povarov [4+2] cycloaddition with 2,3‐dihydrofuran (**2**) by catalytic amounts of achiral and chiral chalcogen bonding catalysts. In this context, we also introduce the first chiral tellurium‐based organocatalysts, which carry chiral aliphatic substituents close to the Lewis acidic center.

## Results and Discussion

**Figure 1:**
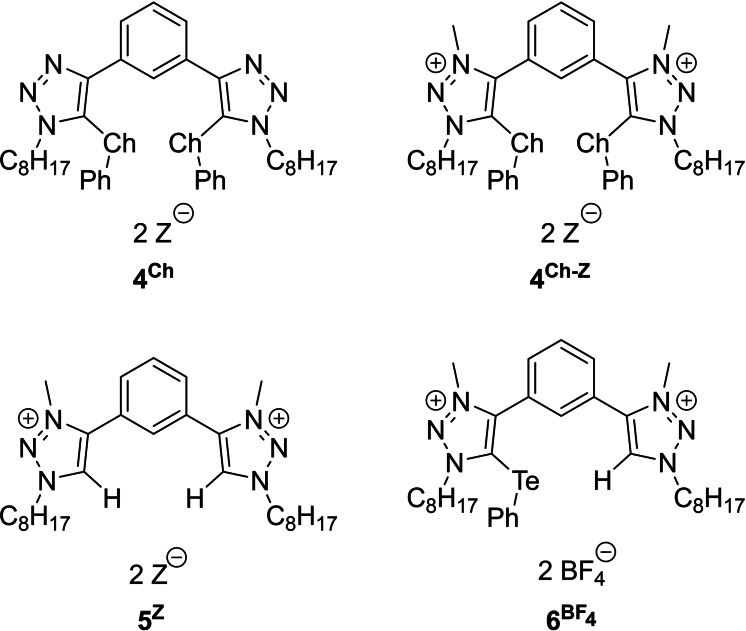
Chalcogen bonding and hydrogen bonding catalysts employed in the Povarov [4+2] Cycloaddition. Ch=Te, Se, S. Z=BAr^F^_4_, BF_4_, OTf.

First, the general ability of chalcogen bond donors to activate imine **1** towards the Povarov [4+2] cycloaddition was checked. Therefore, suitable reactions conditions were investigated using catalyst **4^Te−BArF4^** (Figure 1), as this catalyst proved to be the most active in previous reports.[^22^, ^23^, ^24^] A concentration of 59.8 mM and a catalyst loading of 5 mol % with respect to imine **1** turned out to be suitable. Under these conditions, strong activation of compound **1** was observed with product **3** being formed in 82 % yield after 5 h (TOF=36 h^−1^) and with a (+/−)‐**3** diastereomeric ratio of 47 : 53 (Table 2, entry 1). Nonetheless, the activated imine reacts also with residual water in the solvents, leading to the formation of benzaldehyde and aniline (max. 11 %). In absence of the activation agent, no reaction took place after 24 h (see Table 1, entry 1).

**Table 1:** Yield of (+/−)‐**3** after 5 h in DCM‐*d*_2_ at room temperature in the presence of the listed reference compounds.

Entry	Catalyst	Mol %^[a]^	Yield of (+/−)‐**3** [%]^[b,c]^
1	–	–	<5
2	Te	10	<5
3	Se	10	<5
4	S	10	<5
5	N(CH_3_)_4_BAR^F4^	10	<5
6	N(CH_3_)_4_BF_4_	10	<5
7	N(C_2_H_5_)_4_OTf	10	<5
8	Ph_2_Te_2_	10	<5
9	Ph_2_Se_2_	10	<5
10	Ph_2_S_2_	10	<5
11	**4^Te^**	5	<5
12	**4^Se^**	5	<5
13	**4^S^**	5	<5
14	Benzaldehyde	10	<5
15	Aniline	10	<5
16	**5^BArF4^**	5	<5
17	**5^OTf^**	5	<5
18	**5^BF4^**	5	<5
19	**4^Te‐BArF4^+**Cs_2_CO_3_	5+5	78 (45 : 55)
20	**4^Te‐BArF4^+**N(C_4_H_9_)Cl	5+5	<5

[a] With respect to imine **1**. [b] Determined by 1H NMR spectroscopy. A Measuring error of 5 % is assumed. [c] in brackets the endo : exo ratio.

**Table 2:** Yield of product (+/−)‐**3** after 5 h in DCM‐*d*_2_ in the presence of chalcogen bonding catalysts.

Entry	Catalyst	Mol %^[a]^	Yield of (+/−)‐**3** [%]^[b]^	*endo : exo* ^[b]^	TOF [h^−1^]
1	**4^Te−BArF4^**	5	82	47 : 53	36
2	**4^Te−BF4^**	5	81	68 : 32	17
3	**4^Te−OTf^**	5	64	70 : 30	11
4	**6^BF4^**	5	53	68 : 32	‐
5	**4^Se−BArF4^**	5	26	56 : 44	2.2
6	**4^Se−BF4^**	5	25	72 : 28	2.7
7	**4^Se−OTf^**	5	67	72 : 28	7.4
8	**4^S−BArF4^**	5	<5	–	–
9	**4^S−BF4^**	5	<5	–	–
10	**4^S−OTf^**	5	74	74 : 26	8.8
11	**4^Te−BArF4^**	2.5	69	46 : 54	–
12	**4^Te−BArF4^**	1	44	48 : 52	–

[a] With respect to imine **1**. [b] Determined by ^1^H NMR spectroscopy. A measuring error of 5 % is assumed.

Next, reference compounds were tested to rule out any other activation of the reaction than chalcogen bonding. To this end, elemental tellurium, selenium and sulfur (Table 1, entries 2–4) along with the ammonium salts of the respective counter anions of the herein employed catalysts were applied but all were inactive (Table 1, entries 5–7). The respective diphenyl dichalcogenides (Table 1, entries 8–10) and the neutral precursors of the catalysts (Table 1, entries 11–13) also yielded no product, as did benzaldehyde and aniline (Table 1, entries 14–15). Furthermore, the analogous hydrogen bonding donors (**5^BArF4^**, **5^BF4^**, **5^OTf^**) of the tellurium‐based catalysts did not induce any product formation (Table 1, entries 16–18). Therefore, activation by decomposition products, the anions of the catalysts and even by π‐π interactions can be ruled out. It is especially important to underline that likely decomposition products do not activate the reaction, as approximately 10 % of all tellurium‐based catalysts seem to decompose, forming the corresponding onefold chalcogen containing catalysts (like **6^BF4^**; see Supporting Information Figure S12 and Figure S13). The addition of 5 mol % of cesium carbonate did not inhibit the acceleration of the reaction by catalyst **4^Te−BArF4^**, as similar yields and *endo : exo* ratios were determined, which excludes catalysts by acid traces. Furthermore, the catalyst was selectively deactivated by addition of 5 mol % of tetrabutylammonium chloride to the reaction mixture and no reaction took place (presumably because of inhibition of the catalyst by the more competitive Lewis base). With the help of these experiments, the interaction mode can be pinned down to chalcogen bonding.

**Figure 2:**
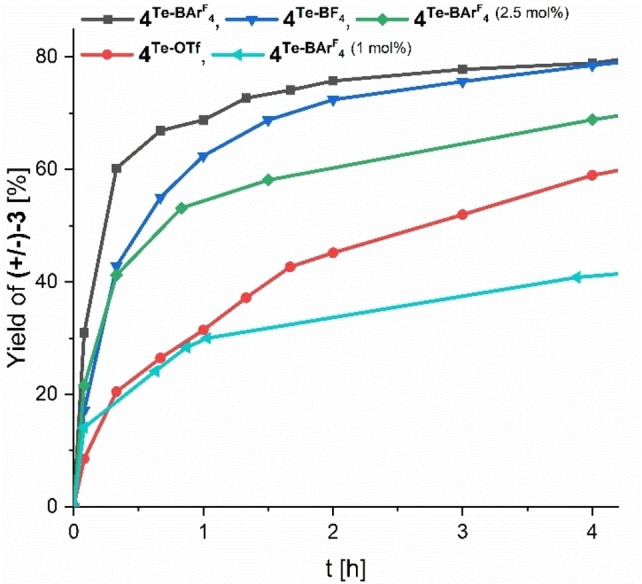
Yield versus time plot of the Povarov [4+2] cycloaddition reaction between **1** and **2**, catalyzed by chalcogen bonding catalysts **4^Te−BArF4^**, **4^Te−BF4^** ^,^ and **4^Te−OTf^**.

Next, the respective tetrafluoroborate salt **4^Te−BF4^** along with the triflate salt **4^Te−OTf^** were tested. The yield obtained with **4^Te−BF4^** (81 %) after 5 h is comparable to the yield obtained with **4^Te−BArF4^** (82 %). Nevertheless, the *endo : exo* ratio shifted markedly in favor or the endo diastereomer (from 47 : 53 to 68 : 32) (Table 2, entries 1 and 2). The yield obtained with **4^Te−OTf^** is smaller compared to the other catalysts (64 %), but the diastereomeric ratio (70 : 30) (Table 2, entry 3) is comparable to the one with **4^Te−BF4^**. Via kinetic profiles (Figure 2), the order of activation is determined as **4^Te−BArF4^** >**4^Te−BF4^** >**4^Te−OTf^**, which is as expected from previous applications of these catalysts.[^22^, ^23^, ^24^] The corresponding turn‐over frequencies (determined after the first 20 min of reaction time) drop from 36 h^−1^ for **4^Te−BArF4^** to 17 h^−1^ for **4^Te−BF4^**. and further to 11 h^−1^ for **4^Te−OTf^**.

The onefold‐tellurated catalyst **6^BF4^** was also implemented in the reaction and a 53 % yield of compound **3** was obtained with a diastereomeric ratio of 68 : 32 (Table 2, entry 4). While the latter is comparable to the one of **4^Te−BF4^**, less product is formed due to the reduced number of Lewis acidic centers (**5^BF4^** is inactive). These observations are in line with the remaining activation being based on chalcogen bonding.

Further, we investigated the behavior of the respective selenium‐based catalysts (Table 1, entries 5–7). With **4^Se−BArF4^**, 26 % yield of (+/−)‐**3** was obtained after 5 h, with a diastereomeric ratio of 56 : 44. A similar yield (25 %. 72 : 28) was found for **4^Se−BF4^**, while more product was formed by using **4^Se−OTf^** (67 %, 72 : 28). Compared to the tellurium‐based chalcogen bonding catalysts, the findings for **4^Se−BArF4^** and **4^Se−BF4^** agree with the expected behavior, as selenium‐based catalysts should show weaker activation due to the less Lewis acidic selenium. Whereas very much comparable *endo : exo* ratios were determined for **4^Te−BF4^** and **4^Se−BF4^**, the ratio for **4^Se−BArF4^** changed compared to **4^Te−BArF4^** and the *endo* diastereomer is now favored. The turn‐over frequency also drops more than one order of magnitude compared to the tellurium‐based catalysts. However, **4^Se−OTf^** in fact activates the reaction very similar to **4^Te−OTf^** in terms of yield, *endo : exo* ratio, and turn‐over frequency. This somewhat suspicious result might already indicate a potential involvement of the counterion in the catalytic pathway. With the help of the sulfur‐containing catalysts, this tendency was further analyzed. Catalysts **4^S−BArF4^** and **4^S−BF4^** do not activate the reaction (Table 2, entries 8 and 9), as the Lewis acidic properties of sulfur are not suitable for this reaction. But tetrahydroquinoline (+/−)‐**3** was formed in 74 % yield with **4^S−OTf^** (Table 2, entry 10). Thus, the triflate salts of the three corresponding catalysts show almost the same activation of the reaction, as similar yields, diastereomeric ratios, and turn‐over frequencies are obtained after 5 h.^[27]^ This indicates a mode of activation in which the triflate actively takes part in the catalytic cycle. Interestingly, without the ChB catalyst, no activation takes place, as tetraethylammonium triflate is inactive on its own (Table 1 entry 7). In an organocatalysis study of this reaction with chiral hydrogen bond (HB) donors in combination with a Brønsted acid, Jacobsen et al. postulated a coordination of the triflate to the protonated imine and the HB donor simultaneously, to provide an ordered environment for asymmetric induction.^[26a]^ Such a model does not seem plausible in our case, not least because here the imine is not protonated. Instead, we speculate that triflate might take part in the follow‐up tautomerism step (somewhat similarly to the role of water in a ChB‐catalyzed Michael addition reaction).^[28]^

Next, we aimed to reduce the catalyst loading, as 5 mol % of **4^Te−BArF4^** already showed a strong acceleration. Indeed, the amount of catalyst could be reduced to 2.5 mol % (Table 1, entry 11) and 1 mol % (Table 1, entry 12), as even these yielded 69 % and 44 % of product. The diastereomeric ratio is still within the boundaries of the results obtained from applying 5 mol % of catalyst. Besides this, these experiments were also used for visual kinetic analysis,^[29]^ which showed that the catalyst acts as a second‐order component in this reaction (see Supporting Information Table S3 and S4, Fig S5 and S6).

To further investigate the chalcogen bonding interaction between **4^Te−BArF4^** and imine **1**, ^1^H NMR experiments were carried out. The signal of the benzylic proton of pure imine **1** is located at 8.48 ppm (see Supporting Information Figure S7). This proton is deshielded to a chemical shift of 8.55 ppm in a 1 : 1 mixture of **1** and **4^Te−BArF4^** and to 8.50 ppm with **4^Te−BF4^**, which is indicative of chalcogen bonding. Due to the overlapping of the different aromatic protons, no change in shifts at the chalcogen bond donor could be observed. ^13^C NMR experiments were subsequently performed and shifts of the imine carbon (N=*C*H) from 160.80 ppm to 161.11 ppm is detected for **4^Te−BF4^** (see Supporting Information Figure S8). Due to overlap of signals belonging to the BAr^F^_4_ anion, no such shift could be noticed for **4^Te−BArF4^** (see Supporting Information Figure S9). Nevertheless, shifts of the tellurium carrying carbon 125.45 ppm to 125.52 ppm for **4^Te−BF4^** and 125.15 ppm to 125.31 ppm for **4^Te−BArF4^** are spotted (see Supporting Information Figure S8 and Fig S9).^[30]^

**Figure 3:**
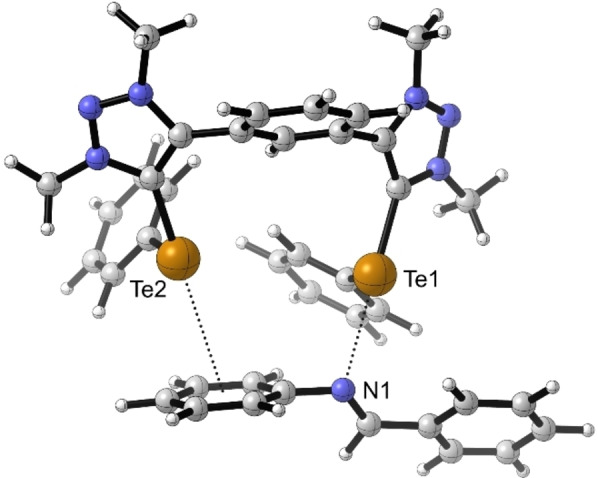
In silico calculated complex between catalyst **4^Te^** and imine **1** (graphics by CYL‐view^[34]^). The all‐methylated analogue of catalyst **4^Te^** was used to reduce computational costs. d_Te1‐N1_=2.86 Å; ∠_C‐Te1‐N1_=169°.

The binding was subsequently also modeled by DFT calculations using the M06‐2X functional^[31]^ including Grimme's D3 dispersion correction^[32]^ with the triple‐zeta def2‐TZVP(D) basis set.^[33]^ The calculations were performed on an all‐methylated analogue of **4^Te^** to reduce the computational costs (see Figure 3). Different from previously reported calculations of this catalyst,^[24]^ the adduct shows a monodentate chalcogen bond from the catalyst (Te1) to the imine nitrogen (N1). The second tellurium (Te2) is coordinated to the π‐system of the aniline moiety of the imine. This also explains the activity of the onefold chalcogenated catalysts **6^BF4^**.

**Figure 4:**
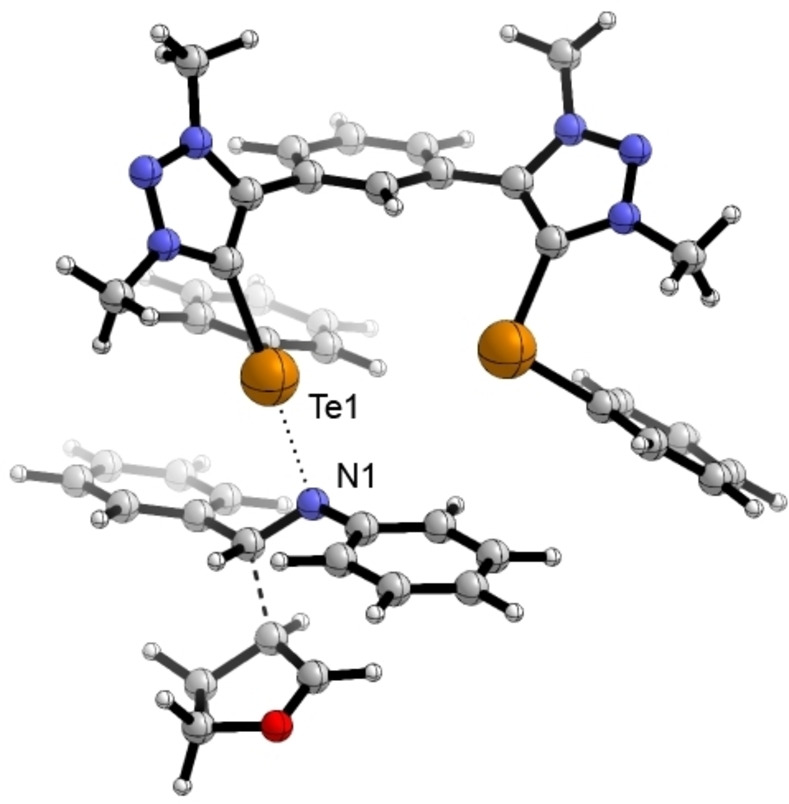
Calculated transition state. The all‐methylated analogue of catalyst **4^Te^** was used to reduce computational costs. d_Te1‐N1_=2.58 Å; ∠_C‐Te1‐N1_=171°.

In addition, we have also computed the likely transition state of the ChB‐catalyzed reaction with the above‐mentioned approach. Here, the conformer of the catalyst with the phenyl groups on Te pointing away from the central phenyl group (like in Figure 3) was part of a transition state that was approx. 2 kcal/mol higher in Gibbs free energy than the more favorable one with the phenyl group pointing towards the core unit (see Figure 4 and Supporting Information). Similar to the complex shown in Figure 3, the transition state also features one strong ChB to the imine nitrogen (d_Te‐N_=2.59 Å, ∠_C‐Te‐N_=171°) and a second ChB involving the π‐system of the nitrogen‐bound phenyl group. The activation barrier is noticeably reduced, from 36 kcal/mol for the uncatalyzed reaction to 29 kcal/mol for the ChB‐catalyzed one.

**Figure 5:**
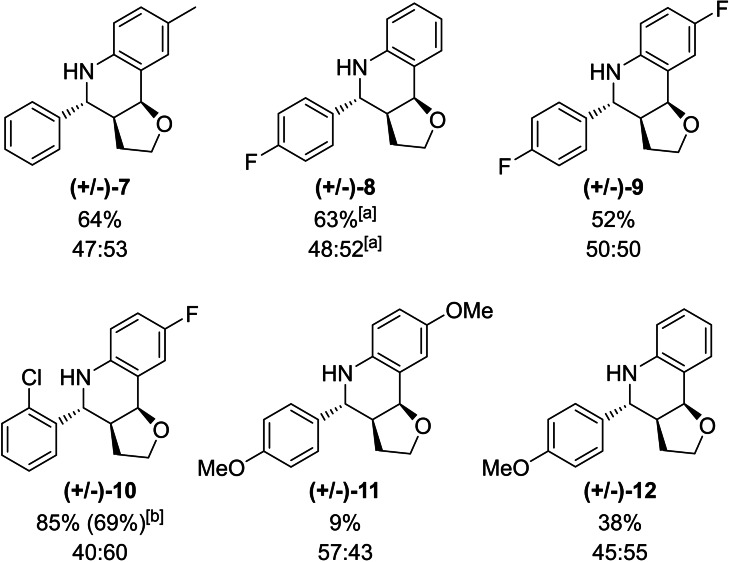
Substrate screening using imines with different electronic properties. [a] after 5 h 20 min, [b] after 1 h.

The scope of the activated imines was then further elucidated experimentally, as different commercially available imines, which feature no background reaction with **2**, were employed in the reaction. Whereas imines with similar electronic properties were successfully activated to give satisfying to good yields ((+/−)‐**7**, (+/−)‐**8**, (+/−)‐**9**, Figure 5) the respective imine of product (+/−)‐**10** was especially easily activated, as 69 % yield was obtained after just one hour. Imines deactivated by electron‐withdrawing substituents were more difficult to activate on the other hand ((+/−)‐**11**, (+/−)‐**12**, Figure 5). Compound (+/−)‐**11** was only obtained in 11 % yield after 5 h. The reasons for this poor activation could either be a coordination of the methoxy group to the chalcogen bond donor (as possibly evidenced by a slight deshielding of the former in NMR spectra ‐ see Supporting Information Figure S10 and S11) or the general deactivation of the substrate through the lower electrophilicity of the imine carbon.

The final reaction parameter that was varied while using achiral catalysts was the solvent. No reaction took place without catalyst **4^Te−BArF4^** in each tested solvent mentioned below.

First, we tested the reaction in chloroform‐*d* (Table 3, entry 2) to check if this cheaper solvent could also be used. In this case, product (+/−)‐**3** was obtained in 70 %. This lower yield compared to DCM‐*d_2_* is partially also due to the lower solubility of **4^Te−BArF4^** in chloroform‐*d*. The diastereomeric ratios are also comparable for these two solvents.

**Table 3:** Yields of (+/−)‐**3** after 5 h using **4^Te−BArF4^** in deuterated solvents with different polarities and nucleophilic properties.

Entry	Solvent	Yield of (+/−)‐**3** [%]^[a,b]^	*endo : exo*
1	DCM‐*d*_2_	82	47 : 53
2	Chloroform‐*d*	70	48 : 52
3	Acetonitrile‐*d*_3_	25	50 : 50
4	Acetone‐*d*_6_	63	47 : 53
5	THF‐*d*_8_	49 (70)	41 : 59 (41 : 59)
6	Methanol‐*d*_4_	10 (22)	74 : 26 (72 : 28)
7	Toluene‐*d*_8_	<5	–

[a] Determined by ^1^H NMR spectroscopy. A measuring error of 5 % is assumed. [b] Results after 24 h in brackets.

Acetonitrile*‐d_3_* as a more polar solvent lead to reduced product formation with an equal ratio of diastereoisomers (Table 3, entry 3). The former could potentially be explained by weaker chalcogen bonding in the more polar environment. Somewhat surprisingly, the reaction takes place in acetone‐*d*_6_ with good yields of 63 % and *endo : exo* ratios similar to other solvents (Table 3, entry 4). In THF‐*d_8_*, the ratio is shifted even slightly more towards the *exo* isomer, albeit with a reduced yield of 49 % (Table 3, entry 5). Increasing the reaction time to 24 h, a 70 % yield of **3** was realized. The more polar and hydrogen‐bonding methanol turned out to be less suitable for this reaction, as only 10 % yield was obtained after 5 h (and still only 22 % after 24 h). Nevertheless, the *endo* diastereomer is formed more selectively in this solvent. Although all components showed good solubility in toluene‐*d*_8_, no reaction took place after 24 h in this solvent.

Finally, we attempted to induce enantioselectivity (by the use of chiral chalcogen bond donors), as was previously already reported for hydrogen bonding organocatalysis.^[26a]^ In this context, chalcogen bonding has the advantage of a second substituent being attached to the divalent chalcogen, in contrast to the (mostly) monovalent halogen centers used for halogen bonding. Therefore, chiral information for enantioselective catalysis could potentially be introduced close to the interaction site. The obvious first approach is to attach chiral aliphatic substituents to the chalcogen via the use of chiral ditellurides. Currently, to the best of our knowledge, no chiral tellurium‐based chalcogen bond donors have been reported.

**Scheme 2:**
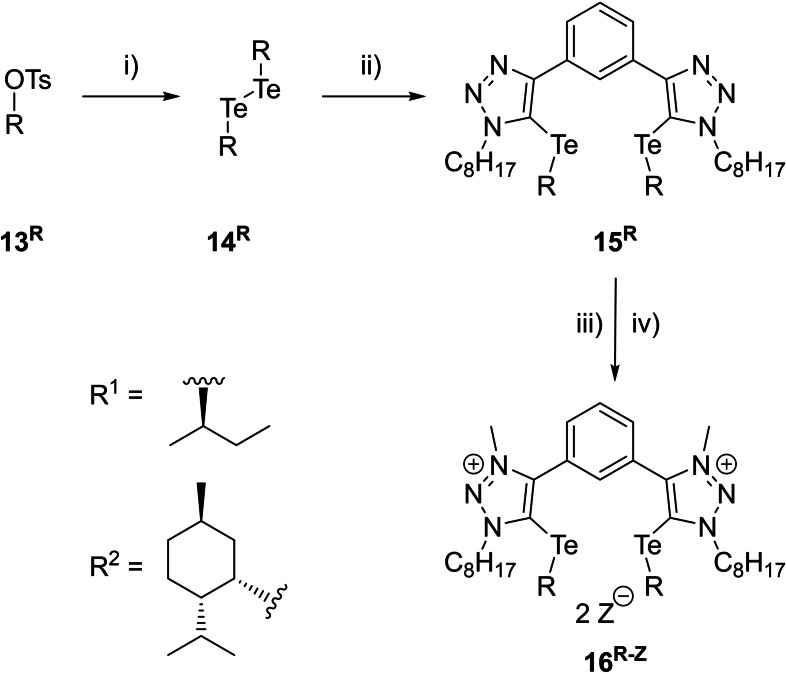
Synthetic pathway for the synthesis of chiral chalcogen bond donors. i) NaBH_4_, Te, DMF EtOH, 70 °C, 1 h→90 °C, 1 h→100 °C, 1 h, 84 % for R^1^, 87 % for R^2^; ii) 1,3‐bistriazolebenzene,^[36]^ *i*‐Pr_2_NH, *n*‐BuLi, THF, −78 °C→rt, 18 h; 23 % for R^1^, 44 % for R^2^ iii) Me_3_OBF_4_, DCM, rt, 18 h, 78 % for R^1^, 67 % for R^2^; iv) TMA BAr^F^_4_, Chloroform, rt, 24 h, 57 % for R^1^ (not performed for R^2^).

First, the respective chiral alcohol is transferred into the corresponding tosylate (**13^R^**), which is then substituted by the nucleophile tellurium species Na_2_Te_2_, giving the corresponding chiral ditelluride (**14^R^**) under inversion of the stereocenter (Scheme 2).^[35]^ Using the reported procedure for the formation of the chalcogen ethers by deprotonation of 1,3‐bistriazolebenzene with LDA and subsequent addition of the ditelluride gave only poor yields of **15^R^**.[^22^, ^23^, ^24^] Therefore, the ditelluride was oxidized by bromine or iodine before addition to the deprotonated triazole scaffold to increase the yield. After oxidation of the ditelluride with iodine, the yield was improved to up to 73 %. However, ^1^H NMR spectroscopy revealed the formation of an inseparable side product. The formation of this side product could not be avoided by using fewer equivalents of iodine with respect to the ditelluride. Similarly, a change of oxidation agent from iodine to bromine gave comparable yields, but also the corresponding side product. Consequently, the already published method without oxidizing the ditelluride had to be used with an excess of the ditelluride (up to 4.5 equiv.), which provided clean product with acceptable yields (up to 44 %). The final methylation and anion metathesis and exchanges followed known procedures.[^22^, ^23^, ^24^] Two different chiral chalcogen bonding catalysts were obtained with this synthetic pathway (see Scheme 2).

**Scheme 3:**
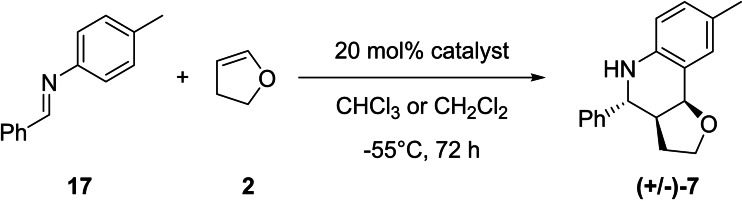
Chiral Povarov [4+2] cycloaddition reaction between *N*‐benzylidene‐p‐toluidine (**17**) and two equivalents of 2,3‐dihydrofuran (DHF) (**2**) in presence of 20 mol % of a chiral catalyst.

For the following study, *N*‐benzylidene‐p‐toluidine (**17**) was used as starting imine, chloroform as the solvent, and the tetrafluoroborate containing compound **4^Te−BF4^** as initial achiral catalyst (Scheme 3). After 3 days of reaction time at −55 °C in dry chloroform with 20 mol % catalyst loading, product (+/−)‐**7** was isolated in 26 % yield with a diastereomeric ratio of 92 : 8 (Table 3, entry 1). Although the tetrafluoroborate salt of the catalyst showed a slower acceleration of the reaction in NMR scale reaction (see Table 1), we decided to use this catalyst because of its higher diastereomeric selectivity compared to the BArF_4_ containing catalysts.

The chiral *sec*‐butyl substituted catalyst **16^R^**_**1**_^**−BF4**^ then provided a comparable yield of 25 % of **7** with a similar *endo : exo* ratio of 94 : 6 (Table 4, entry 2). Unfortunately, no noticeable enantiomeric excess was detected, probably because the side group may be too flexible to enable asymmetric induction in this reaction. An increased steric bulk of the chiral group ‐ in form of catalyst **16^R^**_**2**_^**−BF4**^ ‐ also did not lead to any detectable enantioselectivity (Table 4, entry 3). The diastereomeric selectivity was again comparable to the one obtained with **16^R^**_**1**_^**−BF4**^.

**Table 4:** Yield of product (+/−)‐**7** after 3 days at −55 °C in the presence of 20 mol % chiral chalcogen bonding catalysts.

Entry	Catalyst	Solvent	Yield [%]^[a]^	endo : exo^[b]^	ee [%]
1	**4^Te−BF4^**	CHCl_3_	26	92 : 8	–
2	**16^R^**_**1**_^**−BF4**^	CHCl_3_	25	94 : 6	–
3	**16^R^**_**2**_^**−BF4**^	CHCl_3_	26^[c]^	94 : 6	–
4	**16^R^**_**1**_^**−BArF4**^	CHCl_3_	–	–	–
5	**16^R^**_**1**_^**−BArF4**^	CH_2_Cl_2_	10	57 : 43	–
6	**16^R^**_**1**_^**−BF4**^	CH_2_Cl_2_	20	90 : 10	–

[a] Isolated Yields. [b] Determined by HLPC analysis. [c] After 4 days at −55 °C.

Since the BAr^F^_4_ salts were more active in this reaction (see Figure 2), they might also lead to improved enantioselectivity via shorter binding to the substrate. The surprisingly low solubility of **16^R^**_**1**_^**−BArF4**^ in chloroform at the given concentration and temperature, however, resulted in an oily precipitation of the catalyst during the reaction and in no noticeable activation (Table 4, entry 4). To avoid this problem, chloroform was substituted by dichloromethane. The solubility was indeed improved, but the yield suffered even more and no asymmetric induction was once again achieved (while the diastereomeric ratio went back to almost 50 : 50). This ratio also dropped slightly for the BF_4_ salt **16^R^**_**1**_^**−BF4**^ (Table 4, entry 6) in dichloromethane.

## Conclusion

In conclusion, the activation of imines by tellurium‐based chalcogen bonding catalysts in a Povarov [4+2] cycloaddition reaction was demonstrated, with product yields of up to 82 % after 5 h of reaction time at room temperature. Whereas triflate salts of the catalysts proved to be less suitable for the reaction due to the participation of the anion in the catalytic cycle, BAr^F^_4_ and BF_4_ containing catalysts showed favorable properties. Selenium‐based catalysts were less active in this test reaction, whereas sulfur‐based compounds did not catalyze the reaction. Also, variations of the solvent were tested, which revealed similar properties of *d*_2_‐DCM and *d*‐Chloroform and generally lower yields for solvents with higher polarity. Noticeable changes in the endo : exo diastereomeric ratio of the product were observed for different catalysts and depending on the solvent used. Finally, first chiral triazolium‐based chalcogen bond donors were synthesized and tested in this reaction. However, only racemic mixtures of the product were obtained, as the chiral substituents may still be too flexible. Ongoing work in our group now focuses on the rigidification of these sidearms.

## Experimental Section

All chemicals were purchased from commercially available sources and, if not stated otherwise, used without further purification. Flame dried or oven‐dried glassware was used for all reactions under argon atmosphere along with standard Schlenk techniques. Dry dichloromethane and tetrahydrofuran were received from an MBRAUN MB SPS‐800. The solvents were distilled and dried over 4 Å molecular sieve and finally dried on an alox column. Other dry solvents were dried with flame dried 4 Å molecular sieve. Residual water was determined by a Karl Fischer Titroline® 7500KF trace. Merck TLC aluminum sheets (silica gel 60, F254) were used for thin‐layer chromatography. Substances were detected by fluorescence under UV light (wavelength λ=254 nm). Column chromatography was performed with silica gel (grain size 0.04‐0.063 mm, Merck Si60) and distilled solvents. ^1^H NMR spectra, as well as ^13^C, were recorded with a Bruker AVIII 300 and a Bruker AVIII 400 spectrometer at room temperature. ^19^F NMR were recorded with a Bruker DPX 250 spectrometer at room temperature and were measured proton decoupled if not further noted. ESI‐MS spectra were recorded with a Thermo LTQ XL Orbitrap or a Waters Vion with compounds dissolved in acetonitrile or dichloromethane. IR spectra were recorded with a Shimadzu IR Affinity‐IS spectrometer. For stock solutions, a Mettler Toledo XSR 105 Dual Range balance was used to weight starting material.

**General NMR scale procedure for the Povarov [4+2] cycloaddition**: In a dried NMR tube, 200 μL of a 150 mM stock solution of *N*‐benzylidene aniline (**1**) (29.9 μmol, 5.41 mg, 1.00 equiv.) with 0.125 equiv. tetraethylsilane, 200 μL of a 300 mM stock solution of 2,3‐dihydrofuran (**2**) (58.9 μmol, 4.51 μL, 4.19 mg, 2.00 equiv.) and 100 μL of a 15.0 mM stock solution of the respective catalyst (1.50 μmol, 0.05 equiv.) were mixed. The NMR tube was thoroughly shaken and periodical ^1^H NMR measurements were taken. The yield of the corresponding product was determined as follows: The tetraethylsilane quartet was integrated and set to 1. Afterwards, the integrals of the characteristic signals for product **3** at 5.21 ppm (*endo* diastereomer) and 4.55 ppm (*exo* diastereomer) as well as the N=C*H* signal of **1** at 8.48 ppm and the formed benzaldehyde at 10.02 ppm were added. 100 was divided by this sum. This value was then multiplied by the sum of the integrals for product **3**, giving the overall yield of **3**.

**General asymmetric catalysis procedure for the Povarov [4+2] cycloaddition**: To a flame‐dried Schlenk tube with a septum, 400 μL of a 250 mM stock solution of *N*‐Benzylidene‐p‐toluidine (**17**) (100 μmol, 19.5 mg, 1.00 equiv.) were added and cooled to −55 °C. After 10 minutes, 400 μL of a 50.0 mM stock solution of the respective catalyst (20.0 μmol, 0.20 equiv.) were added. After another 10 minutes, 200 μL of a 1.00 M stock solution of 2,3‐dihydrofuran (**2**) (200 μmol, 15,1 μL, 14.0 mg, 2.00 equiv.) were added. The mixture was stirred for 72 h at −55 °C and then quenched with 14.0 μL triethylamine (1.00 eq). After warming to room temperature, the solvent was evaporated under reduced pressure and purified by column chromatography using pentane : ethyl acetate 10 : 1 (R_f_=0.52 (*exo*), R_f_=0.48 (*endo*)). The obtained diastereomeric mixture was analyzed by chiral HPLC using a Dr. Maisch ReproSil Chiral‐OM 5 μm column, 1.5 mL/min flow rate with hexane:isopropanol 96 : 4 (retention times: 5.75 min and 13.72 min for the *exo*‐enantiomers, 8.12 min and 11.05 min for the *endo*‐enantiomers). The spectroscopic data of the diastereomeric mixtures are in agreement with the literature.^[26a]^

## Conflict of interest

The authors declare no conflict of interest.

1

## Supporting information

As a service to our authors and readers, this journal provides supporting information supplied by the authors. Such materials are peer reviewed and may be re‐organized for online delivery, but are not copy‐edited or typeset. Technical support issues arising from supporting information (other than missing files) should be addressed to the authors.

Supporting Information

## Data Availability

The data that support the findings of this study are available from the corresponding author upon reasonable request.
