# Supplementary material for: Catalytic Activation of Imines by Chalcogen Bond Donors in a Povarov [4+2] Cycloaddition Reaction
Source: Chemistry. 2022 Jul 12;28(47):e202200917. doi: 10.1002/chem.202200917 (PMC9545453; doi:10.1002/chem.202200917)
Supplement: Supplementary file 1 — Supporting Information [file CHEM-28-0-s001.pdf]

Chemistry–A European Journal

Supporting Information

Catalytic Activation of Imines by Chalcogen Bond Donors in a Povarov [4+2] Cycloaddition Reaction

Tim Steinke, Patrick Wonner, Richard M. Gauld, Sascha Heinrich, and Stefan M. Huber*

Table of Contents

1 Experimental Section 2

1.1 Experimental Conditions 2

1.2 Analysis Methods 2

2 Synthesis of known compounds 3

3 Synthesis of new compounds 3

3.1 General procedure for the formation of chiral ditellurides 3

3.2 General procedure for the formation of chiral chalcogen ethers 4

3.3 General procedure for the methylation of chalcogen ethers 7

3.4 Products of the Povarov [4+2] cycloaddition 11

4 Catalysis experiments 17

4.1 Yield determination for NMR scale Povarov [4+2] cycloaddition 17

4.2 Determination of TOF numbers 20

4.3 Visual Kinetic Analysis 21

4.4 ^{1}H NMR Experiments on the interaction between imines and $4^{Te\text{-}BArF_4}$ and the catalyst stability 24

5 ^{1}H, ^{13}C, ^{19}F NMR spectra 30

6 DFT Calculations 46

1 Experimental Section

1.1 Experimental Conditions

Commercially available chemicals were purchased from *ABCR, Alfa Aesar, Carbolution, Merck, ChemPur, Sigma Aldrich, Roth or VWR* and were used without further purification. All experiments were carried out under argon atmosphere with dry solvents and flame dried glassware using standard *Schlenk* techniques. Dry dichloromethane, diethyl ether and tetrahydrofuran were received from a *MBRAUN MB SPS-800*. The solvents were distilled and dried over 4 Å molecular sieve and finally dried on an alox column. Additional dry solvents were dried with flame dried 4 Å molecular sieve. A Karl Fischer *Titroline® 7500KF trace* was used to determine residual water. *Merck TLC aluminium sheets* (silica gel 60, F254) were used for thin layer chromatography. Substances were detected by fluorescence under UV light (wavelength λ = 254 nm). Column chromatography was performed with silica gel (grain size 0.04-0.063 mm, *Merck Si60*) and distilled solvents. The used solvents as eluents with the corresponding R_f values are listed for the corresponding experiments.

1.2 Analysis Methods

1.2.1 NMR Spectroscopy

^{1}H NMR spectra as well as ^{13}C NMR were recorded with a *Bruker AVIII 300* and a *Bruker AVIII 400* spectrometer at room temperature. ^{19}F NMR spectra were recorded with a *Bruker DPX-250* spectrometer at room temperature and were measured proton decoupled if not further noted. Chemical shifts are given in parts per million (ppm) (δ scale) and are referenced to residual ^{1}H and ^{13}C signals from deuterated solvents. Multiplicities are displayed using the following abbreviations: s = singlet, d = doublet, dd = doublet of doublet, t = triplet, td = triplet of doublet, m = multiplet. Spin-spin coupling constants (*J*) are given in Hz.

1.2.2 ESI-MS Measurements

ESI-MS spectra were recorded with a Thermo LTQ XL Orbitrap or a Waters Vion with compounds dissolved in acetonitrile or dichloromethane.

1.2.3 ATR-IR Measurements

IR spectra were recorded with a *Shimadzu IR Affinity-IS* spectrometer. Signals are reported in $\nu = cm^{-1}$ and are indicated as follows: vs = very strong, s = strong, m = middle, w = weak.

1.2.4 Specific rotation

The optical rotations were measured using an Anton Paar Propol polarimeter with a path length of 0.5 dm, a wavelength of 589 nm at 25°C.

1.2.5 Balance

For stock solutions a *Mettler Toledo XSR 105 Dual Range* balance was used to weigh starting material.

2 Synthesis of known compounds

Octyl azide,[1] 1,3-bistriazolebenzene,[2] **SI-1**^{Te},[3] **SI-1**^{Se},[3] **SI-1**^{S},[3] **4**$^{Te\text{-}BAr^F{}_4}$,[3] **4**$^{Te\text{-}BF_4}$,[3] **4**$^{Te\text{-}OTf}$,[4] **4**$^{Se\text{-}BAr^F{}_4}$,[5] **4**$^{Se\text{-}BF_4}$,[3] **4**$^{Se\text{-}OTf}$,[4] **4**$^{S\text{-}BAr^F{}_4}$,[5] **4**$^{S\text{-}BF_4}$,[3] **4**$^{S\text{-}OTf}$[4], **5**$^{BAr^F{}_4}$,[5] **5** ^{BF_4},[3] **5**^{OTf}[2] and **6**^{BF_4}[3] were synthesized according to literature procedures.

3 Synthesis of new compounds

3.1 General procedure for the formation of chiral ditellurides

Chiral ditellurides were synthesized according to a literature procedure.[6] In a flame dried two-neck Schlenk flask with reflux condenser, sodium borohydride (1.05 eq.) tellurium powder (1.0 eq) were added and a 1:1 mixture of dry DMF and ethanol (0.50 M) was slowly added dropwise. The resulting suspension is stirred at 70°C for 1 hour. To the clear, slightly red mixture, another 1.0 eq. of tellurium powder is added and the mixture stirred at 90°C for 1 hour. Afterwards, the mixture is cooled to room temperature and the respective tosylate in dry DMF (2.00 M) is added. The mixture is stirred for 1 hour at 100°C. After cooling to room temperature, the mixture is poured onto water, extracted three times with pentane and dried over magnesium sulfate. After removal of the solvent under reduced pressure, the respective chiral tosylates are obtained and were used without further purification.

3.1.1 Synthesis of 14^{R1}

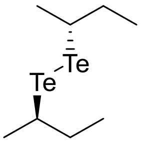

Compound **14^{R1}** was synthesized according to the general procedure for the formation of chiral ditellurides using 0.722 g sodium borohydride (19.1 mmol, 1.05 eq.), 2.32 g tellurium powder (18.2 mmol, 1.0 eq) in 36.4 mL dry DMF:EtOH (0.5 M, 1:1). Also, 4.15 g **13^{R1}** (18.2 mmol, 1.00 eq.) in 9.10 mL dry DMF (2.00 M) was used. The product was obtained as a red liquid and was used without further purification. Yield: 2.83 g (7.65 mmol, 84%).

^{1}H NMR (300 MHz, Chloroform-*d*): 3.31 – 3.16 (m, 1 H, CH_3-C***H***-CH_2-CH_3), 1.78 – 1.58 (m, 5 H, C$\boldsymbol{H}_3$-CH-C$\boldsymbol{H}_2$-CH_3), 0.96 (t, ^3J = 7.3 Hz, 3 H, CH_3-CH-CH_2-C$\boldsymbol{H}_3$)

3.1.2 Synthesis of 14^{R2}

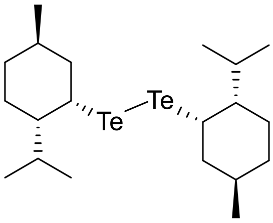

Compound **14^{R2}** was synthesized according to the general procedure for the formation of chiral ditellurides using 0.768 g sodium borohydride (20.3 mmol, 1.05 eq.), 2.47 g tellurium powder (19.3 mmol, 1.0 eq) in 38.6 mL dry DMF:EtOH (0.5 M, 1:1). Also, 6.00 g **13^{R1}** (19.3 mmol, 1.00 eq.) in 19.3 mL dry DMF (1.00 M) was used. The product was obtained as a red liquid which formed a wax-like solid after storage in a freezer and was used without further purification. The spectroscopic data is in agreement with literature data.[6] Yield: 4.48 g (8.39 mmol, 87%).

3.2 General procedure for the formation of chiral chalcogen ethers

An already published procedure was modified for the general procedure for the formation of chiral chalcogen ethers.[3-4] In a flame-dried Schlenk flask, *i*-Pr_2NH (2.20 eq.) was added to dry THF (0.1 M with respect to the triazole) and cooled to 0°C. Afterwards, *n*-BuLi (2.40 eq., 2.50 M solution in hexane) was added dropwise over the course of 15 minutes and the resulting solution was stirred for 30 minutes at 0°C. Subsequently, the solution was cooled to -78°C, stirred for 15 minutes and

1,3-bistriazolebenzene[2] (1.00 eq.) dissolved in dry THF (0.1 M) added dropwise. The resulting mixture was stirred for 3 hours at -78°C. To this mixture, the respective chiral ditelluride (4.50 eq.) dissolved in dry THF (0.1 M) was added and the mixture was stirred and slowly warmed to room temperature for 18 h. The solvent was removed under reduced pressure and the crude product purified by column chromatography to give the respective chiral chalcogen ether.

3.2.1 Synthesis of 15^{R1}

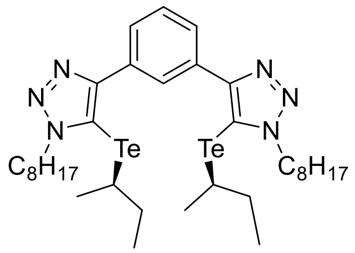

Compound $\mathbf{15^{R1}}$ was synthesized after the general procedure for the formation of chiral chalcogen ethers using 0.354 mL *i*-Pr_2NH (0.255 g, 2.52 mmol, 2.20 eq.) in 11.5 mL dry THF (0.1 M with respect to the triazole) and 1.10 mL of a 2.5 M *n*-BuLi solution (2.75 mmol, 2.40 eq.). Furthermore, 0.500 g 1,3-bistriazolebenzene (1.15 mmol, 1.00 eq.) in 11.5 mL THF (0.1 M) and 0.931 g $\mathbf{14^{R1}}$ (2.52 mmol, 2.52 eq.) in 25.2 mL dry THF (0.1 M) were used. After purification by column chromatography using Pentane:EtOAc 3:1 $\mathbf{15^{R1}}$ was obtained as an orange oil ($[\alpha]_D^{25}$= -25.8, 0.1 g/100 mL, DCM). Yield: 0.213 g (0.265 mmol, 23 %).

R_f = 0.50 (Pentane:EtOAc 3:1).

^{1}H NMR (300 MHz, Chloroform-*d*):
δ [ppm] = 8.69 (t, ^4J = 1.4 Hz, 1 H, $C_{triaz.}$-C-C***H***-C-$C_{triaz.}$), 8.05 (dd, ^3J = 7.8 H, ^4J = 1.3 Hz, 2 H, C-C***H***-CH-C***H***-C), 7.51 (t, ^3J = 7.8 Hz, 1 H, C-CH-C***H***-CH-C), 4.59 (t, ^3J = 7.4 Hz, 4 H, $N_{triaz.}$-C$\boldsymbol{H}_2$-CH_2), 3.34 (h, ^3J = 7.2 Hz, 2 H, CH_3-C***H***-Te), 1.94 (p, ^3J = 7.3 Hz, 4 H, $N_{triaz.}$-CH_2-C$\boldsymbol{H}_2$), 1.55 (p, ^3J = 7.1 Hz, 4 H, Te-CH-C$\boldsymbol{H}_2$), 1.44 – 1.20 (m, 26 H, C$\boldsymbol{H}_{aliph.}$, C$\boldsymbol{H}_3$-CH-Te), 0.93 – 0.79 (m, 12 H, CH_2-CH_2-C$\boldsymbol{H}_3$, Te-CH-CH_2-C$\boldsymbol{H}_3$).

^{13}C NMR (101 MHz, Chloroform-*d*):
δ [ppm] = 154.0 ($C_{arom.}$), 132.1 ($C_{arom.}$), 128.4 ($C_{triaz.}$-C-***C***H-C-$C_{triaz.}$), 128.4 (C-CH-***C***H-CH-C), 128.1 (C-***C***H-CH-***C***H-C), 100.2 ($C_{arom.}$), 51.4 ($N_{triaz.}$-***C***H_2-CH_2), 32.4 (Te-CH-

CH_2), 31.9 ($C_{aliph.}$), 31.2 ($C_{aliph.}$), 30.5 (CH_3-***C***H-Te), 29.3 ($C_{aliph.}$), 26.7 ($C_{aliph.}$), 23.6 (***C***H_3-CH-Te), 22.8 (CH_2-***C***H_2-CH_3), 14.3 (Te-CH-CH_2-***C***H_3), 13.8 (CH_2-CH_2-***C***H_3).

ATR-IR:

$\tilde{\nu}$ [cm^{-1}] = 3063 (w), 2951 (s), 2922 (vs), 2853 (s), 1609 (w), 1585 (w), 1452 (vs), 1375 (m), 1323 (m), 1217 (w), 1184 (m), 1128 (m), 997 (m), 982 (m), 905 (w), 799 (s), 721 (s), 696 (s).

ESI-MS:

m/z (+) = calc. 805.2735 $[M+H]^+$; found 805.2736 $[M+H]^+$.

3.2.2 Synthesis of 15^{R2}

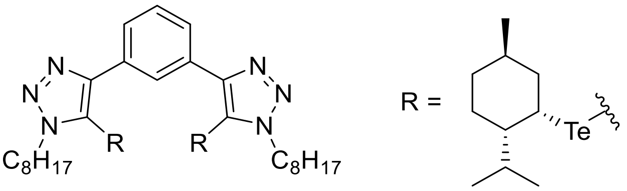

Compound $\mathbf{15^{R2}}$ was synthesized after the general procedure for the formation of chiral chalcogen ethers using 0.567 mL *i*-Pr_2NH (0.408 g, 4.03 mmol, 2.20 eq.) in 18.3 mL dry THF (0.1 M with respect to the triazole) and 1.76 mL of a 2.5 M *n*-BuLi solution (4.40 mmol, 2.40 eq.). Furthermore, 0.800 g 1,3-bistriazolebenzene (1.83 mmol, 1.00 eq.) in 18.3 mL THF (0.1 M) and 4.40 g $\mathbf{14^{R2}}$ (8.24 mmol, 4.50 eq.) in 82.4 mL dry THF (0.1 M) were used. After purification by column chromatography using Pentane:EtOAc 5:1 $\mathbf{15^{R2}}$ was obtained as an orange oil ($[\alpha]_D^{25}$= 24.2, 0.1 g/100 mL, DCM). Yield: 0.773 g (0.798 mmol, 44%).

$\mathbf{R_f}$ = 0.50 (Pentane:EtOAc 5:1).

^{1}H NMR (300 MHz, Chloroform-*d*):

δ [ppm] = 8.71 (d, ^4J = 1.5 Hz, 1 H, $C_{triaz.}$-C-C***H***-C-$C_{triaz.}$), 8.07 (dd, ^3J = 7.8 Hz, ^4J = 1.8 Hz, 2 H, C-C***H***-CH-C***H***-C), 7.50 (t, ^3J = 7.8 Hz, 1 H, C-CH-C***H***-CH-C), 4.57 (d, ^3J = 7.6 Hz, 4 H, $N_{triaz.}$-C***H***$_2$-CH_2), 3.85 (d, ^4J = 2.4 Hz, 2 H, C***H***$_{aliph.}$), 1.96 (p, ^3J = 7.7 Hz, 4 H, $N_{triaz.}$-CH_2-C***H***$_2$), 1.79 (dt, ^3J = 13.9, ^4J = 2.4 Hz, 2 H, C***H***$_{aliph.}$), 1.66 (dt, ^3J = 14.2 Hz, ^4J = 2.9 Hz, 4 H, C***H***$_{aliph.}$), 1.44 – 1.25 (m, 24 H, C***H***$_{aliph.}$), 0.97 – 0.80 (m, 20 H,

C*H*$_{aliph.}$), 0.69 (d, *J* = 6.3 Hz, 6 H, C*H*$_3$-CH-CH$_3$), 0.62 (d, *J* = 6.5 Hz, 6 H, CH$_3$-CH-C*H*$_3$).

^{13}C NMR (101 MHz, DCM-*d*$_2$):

δ [ppm] = 153.9, 132.9, 128.8, 128.4, 128.2, 99.8, 71.9, 51.8, 50.9, 50.2, 44.50 (t, *J* = 99.9 Hz), 35.39 (d, *J* = 25.6 Hz), 34.5, 32.4, 32.3, 31.6, 30.9, 29.7, 29.7, 29.3, 27.3, 26.5, 23.8, 23.2, 22.6, 22.1, 21.4, 20.34 (d, *J* = 11.6 Hz), 16.5, 14.5.

ATR-IR:

$\tilde{\nu}$ [cm^{-1}] = 2949 (s), 2920 (vs), 2866 (s), 2853 (s), 1454 (s), 1368 (m), 1314 (w), 1261 (m), 1225 (m), 1165 (m), 1103 (w), 1045 (m), 1024 (m), 982 (w), 918 (w), 853 (w), 800 (m), 721 (w), 696 (m), 642 (w), 548 (w).

ESI-MS:

m/*z* (+) = calc. 969.4300 [M+H]$^+$; found 969.4302 [M+H]$^+$.

3.3 General procedure for the methylation of chalcogen ethers

An already published procedure was modified for the general procedure for the formation of chiral chalcogen ethers.[3-4] The respective chalcogen ether (1.00 eq.) was dissolved in dry DCM (0.05 M) and subsequently trimethyloxonium tetrafluoroborate (2.50 eq.) was added to the solution. The mixture was stirred for 18 hours at room temperature. The solvent was removed under reduced pressure and the residue washed five times with Et$_2$O and five times with pentane in an ultrasonic bath. Afterwards, the obtained sticky solid was dried under high vacuum to obtain the respective methylated compound.

3.3.1 Synthesis of 16$^{R1\text{-}BF_4}$

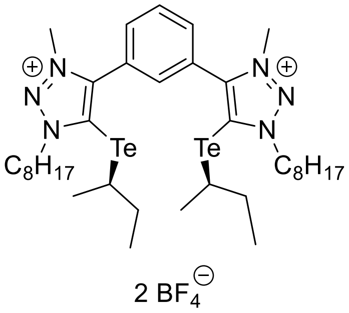

Compound **16$^{R1\text{-}BF_4}$** was synthesized after the general procedure for the methylation of chalcogen ethers using 0.230 g **15^{R1}** (0.286 mmol, 1.00 eq.) in 5.7 mL dry DCM

(0.05 M) and 0.106 g trimethyloxonium tetrafluoroborate (0.715 mmol, 2.50 eq.). After washing with Et_2O (5x20 mL) and pentane (5x20 mL) and drying under high vacuum, **$16^{R1\text{-}BF4}$** was obtained as a yellow sticky solid ($[\alpha]_D^{25}$ = -16.0, 0.1 g/100 mL, DCM). Yield: 0.225 mg (0.223 mmol, 78%).

^{1}H NMR (300 MHz, Chloroform-*d*):

δ [ppm] = 8.13 (t, ^{4}J = 1.4 Hz, 1 H, $C_{triaz.}$-C-C***H***-C-C_{triaz}), 7.99 (dd, ^{3}J = 7.4 Hz, ^{4}J = 1.7 Hz, 2 H, C-C***H***-CH-C***H***-C), 7.85 (t, J = 7.5 Hz, 1 H, C-CH-C***H***-CH-C), 4.74 (t, ^{3}J = 7.9 Hz, 4 H, $N_{triaz.}$-C$\boldsymbol{H}_2$-CH_2), 4.26 (s, 6 H, $N_{triaz.}$-C$\boldsymbol{H}_3$), 3.48 (q, ^{3}J = 6.9 Hz, 2 H, CH_3-C***H***-Te), 2.11 (p, ^{3}J = 6.9 Hz, 4 H, $N_{triaz.}$-CH_2-C$\boldsymbol{H}_2$), 1.69 – 1.24 (m, 30 H, C$\boldsymbol{H}_{aliph}$, Te-CH-C$\boldsymbol{H}_2$-CH_3, C$\boldsymbol{H}_3$-CH-Te), 0.95 – 0.79 (m, 12 H, CH_2-CH_2-C$\boldsymbol{H}_3$, Te-CH-CH_2-C$\boldsymbol{H}_3$).

^{13}C NMR (101 MHz, DCM-*d*$_2$):

δ [ppm] = 150.0, 137.7, 133.8, 131.2, 125.8. 111.81, 56.1, 39.7, 34.4, 33.0, 29.50 (d, J = 8.8 Hz), 26.9, 24.7, 23.2, 14.4, 14.0.

^{19}F NMR (235 MHz, Chloroform-*d*):

δ [ppm] = -153.2 (d, J = 11.9 Hz, B$\boldsymbol{F}_4$).

ATR-IR:

$\tilde{\nu}$ [cm^{-1}] = 2955 (w), 2924 (m), 2855 (w), 1736 (w), 1541 (w), 1456 (m), 1377 (w), 1319 (w), 1285 (), 1047 (s), 1030 (s), 843 (m), 816 (w), 764 (w), 704 (w), 660 (w), 623 (w), 519 (m).

ESI-MS:

m/*z* (+) = calc. 921.3155 $[M\text{-}BF_4]^+$, 417.1560 $[M\text{-}2BF_4]^{2+}$; found 921.3155 $[M\text{-}BF_4]^+$, 417.1558 $[M\text{-}2BF_4]^{2+}$.

m/*z* (-) = calc. 87.0035 $[BF_4]^-$; found 87.0024 $[BF_4]^-$.

3.3.2 Synthesis of $16^{R1\text{-}BAr^F_4}$

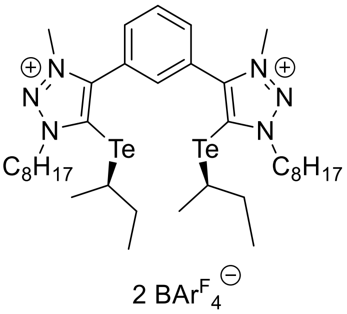

Compound **$16^{R1\text{-}BAr^F_4}$** was synthesized after an already published procedure.[3] In a flame-dried Schlenk flask, 0.296 mg **$16^{R1\text{-}BF_4}$** (0.293 mmol, 1.00 eq.) were dissolved in 30 mL dry chloroform (0.01 M) and 0.577 g tetramethylammonium tetrakis[3,5-bis(trifluoromethyl)phenyl]borate (0.616 mmol, 2.50 eq.) were added. The mixture was stirred for 18 hours at room temperature and the solvent was removed under reduced pressure afterwards. The resulted residue was dissolved in the minimum amount of Et_2O and cooled to -78°C until a precipitate was formed. The precipitate was filtered off and washed with -78°C cold Et_2O. The solvent was again removed under reduced pressure and the residue was dissolved in the minimum amount of chloroform. The solution was cooled to -55°C and the formed precipitate filtered off and washed with -55°C cold chloroform. After removing the solvent under reduced pressure and drying under high vacuum, **$16^{R1\text{-}BAr^F_4}$** was obtained as a yellow solid ($[\alpha]_D^{25}$ = -7.8, 0.1 g/100 mL, DCM). Yield: 0.428 g (0.167 mmol, 57%).

^{1}H NMR (300 MHz, DCM-*d₂*):

δ [ppm] = 7.94 (t, ^3J = 7.1 Hz, 1 H, C-CH-C***H***-CH-C), 7.77 (dd, ^3J = 7.7 Hz, ^4J = 2.0 Hz, 2 H, C-C***H***-CH-C***H***-C), 7.71 (s, 16 H, C***H***-C(CF_3)-CH-C(CF_3)-C***H***), 7.61 (d, ^4J = 2.0 Hz, 1 H, $C_{triaz.}$-C-C***H***-C-C_{triaz}), 7.55 (s, 8 H, CH-C(CF_3)-C***H***-C(CF_3)-CH), 4.78 (t, ^3J = 7.5 Hz, 4 H, $N_{triaz.}$-C***H***$_2$-CH_2), 4.20 (s, 6 H, $N_{triaz.}$-C***H***$_3$), 3.47 (d, ^3J = 7.0 Hz, 2 H, CH_3-C***H***-Te), 2.06 (d, ^3J = 7.2 Hz, 3 H, $N_{triaz.}$-CH_2-C***H***$_2$), 1.64 – 1.24 (m, 30 H, C***H***$_{aliph}$, Te-CH-C***H***$_2$-CH_3, C***H***$_3$-CH-Te), 0.95 – 0.76 (m, 12 H, CH_2-CH_2-C***H***$_3$, Te-CH-CH_2-C***H***$_3$).

^{13}C NMR (101 MHz, DCM-*d₂*):

δ [ppm] = 162.3 (dd, *J* = 99.6, 49.9 Hz), 149.6, 135.4, 134.6, 132.7, 132.5, 130.6, 129.5 (ddd, *J* = 31.6, 5.9, 2.9 Hz), 127.0, 125.5, 123.4, 119.8, 118.1, 112.4, 68.4, 56.9, 39.8, 35.9, 33.0, 32.2, 30.6, 29.40 (d, *J* = 11.0 Hz), 26.8, 26.2, 24.5, 23.1, 14.3, 14.0.

^{19}F NMR (235 MHz, DCM-*d₂*):

δ [ppm] = -62.8 (s, C***F***$_3$).

ATR-IR:

$\tilde{\nu}$ [cm^{-1}] = 2961 (w), 1932 (w), 1862 (w), 1611 (w), 1460 (w), 1352 (s), 1271 (vs), 1111 (vs), 945 (w), 932 (w), 885 (m), 839 (m), 810 (), 745 (), 712 (s), 681 (s), 669 (s), 619 (w), 581 (w), 449 (w).

ESI-MS:

m/z (-) = calc. 1697.3775 [M-BAr$^F_4$] $^+$; 417.1560 [M-2BAr$^F_4$]$^{2+}$; found 1697.3777 [M-BAr$^F_4$] $^+$; 417.1556 [M-2BAr$^F_4$]$^{2+}$.

m/z (-) = calc. 863.0654 [BAr$^F_4$]$^-$; found 863.0641 [BAr$^F_4$]$^-$.

3.3.3 Synthesis of 16$^{R2\text{-}BF4}$

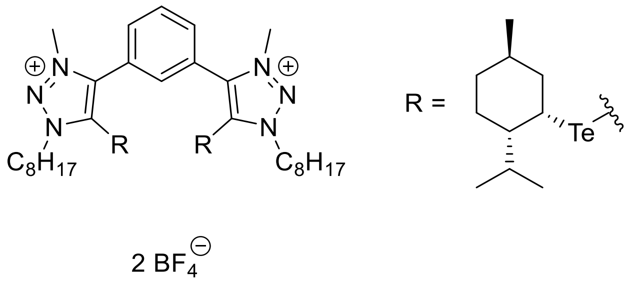

Compound **16$^{R2\text{-}BF4}$** was synthesized after the general procedure for the methylation of chalcogen ethers using 0.645 g **15^{R2}** (0.666 mmol, 1.00 eq.) in 13.3 mL dry DCM (0.05 M) and 0.246 g trimethyloxonium tetrafluoroborate (1.67 mmol, 2.50 eq.). After washing with Et_2O (5x30 mL) and pentane (5x30 mL) and drying under high vacuum, **16$^{R2\text{-}BF4}$** was obtained as a yellow sticky solid ($[\alpha]_D^{25}$= 51.8, 0.1 g/100 mL, DCM). Yield: 0.521 mg (0.445 mmol, 67%).

^{1}H NMR (300 MHz, Chloroform-*d*):

δ [ppm] = 8.09 (d, ^4J = 1.6 Hz, 1 H, C$_{triaz.}$-C-C***H***-C-C$_{triaz.}$), 8.01 (dd, ^3J = 7.4, ^4J = 1.7 Hz, 2 H, C-C***H***-CH-C***H***-C), 7.87 (d, ^3J = 7.6 Hz, 1 H, C-CH-C***H***-CH-C), 4.71 (d, ^3J = 8.1 Hz, 4 H, N$_{triaz.}$-C***H***$_2$-CH$_2$), 4.25 (s, 6 H, N$_{triaz.}$-C***H***$_3$), 4.04 (t, ^4J = 1.8 Hz, 2 H, C***H***$_{aliph.}$), 2.12 (d, ^3J = 7.8 Hz, 4 H, N$_{triaz.}$-CH$_2$-C***H***$_2$), 1.87 (d, ^3J = 11.0 Hz, 2 H, C***H***$_{aliph.}$), 1.76 – 1.58

(m, 6 H, C***H***$_{aliph.}$), 1.53 – 1.25 (m, 24 H, C***H***$_{aliph.}$), 0.95 – 0.78 (m, 22 H, C***H***$_{aliph.}$), 0.65 (d, *J* = 6.5 Hz, 6 H, C***H***$_{aliph.}$).

^{13}C NMR (101 MHz, Chloroform-*d*):

δ [ppm] = 149.3, 134.3, 133.7, 130.6, 125.4, 110.3, 55.5, 46.7, 47.3, 43.3, 43.3, 39.2, 34.8, 34.3, 31.9, 30.5, 29.7, 29.4, 29.1 (d, *J* = 10.0 Hz), 26.6, 22.7, 21.8, 20.5, 14.2.

^{19}F NMR (235 MHz, DCM-*d*$_2$):

δ [ppm] = -152.8 (d, *J* = 12.0 Hz, B***F***$_4$).

ATR-IR:

$\tilde{\nu}$ [cm^{-1}] = 3059 (w), 2924 (m), 2855 (m), 1543 (w), 1456 (m), 1368 (w), 1317 (w), 1285 (w), 1223 (w), 1167 (w), 1047 (vs), 1032 (vs), 851 (w), 818 (w), 704 (w), 660 (w), 557 (w), 521 (m), 478 (w).

ESI-MS:

m/*z* (-) = calc. 1085.4720 [M-BF$_4$]$^+$, 499.2343 [M-2BF$_4$]$^{2+}$; found 1085.4716 [M-BF$_4$]$^+$, 499.2339 [M-2BF$_4$]$^{2+}$.

m/*z* (-) = calc. 87.0035 [BF$_4$]$^-$; found 87.0024 [BF$_4$]$^-$.

3.4 Products of the Povarov [4+2] cycloaddition

3.4.1 Synthesis of (+/-)-3

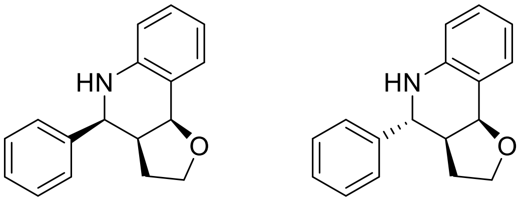

endo exo

Compound **(+/-)-3** was synthesized after the general NMR scale procedure for the Povarov [4+2] cycloaddition (see manuscript). The spectroscopic data are in agreement with literature data.[7]

3.4.2 Synthesis of (+/-)-7

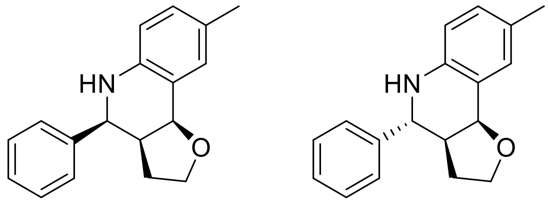

endo exo

Compound **(+/-)-7** was synthesized after the general NMR scale procedure for the Povarov [4+2] cycloaddition (see manuscript). The spectroscopic data are in agreement with literature data.[7a-c]

3.4.3 Synthesis of (+/-)-8

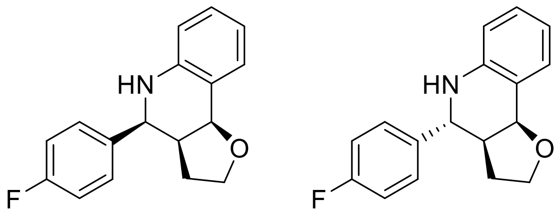

endo exo

For characterisation, compounds **(+/-)-8** were synthesized after the general asymmetric catalysis procedure for the Povarov [4+2] cycloaddition (see manuscript) at RT. The diastereomers were separated by column chromatography using Pentane:EtOAc 10:1.

R_f: endo diastereomer = 0.44, exo diastereomer = 0.36 (Pentane:EtOAc 5:1).

^{1}H NMR (300 MHz, DCM-d_2):

δ [ppm] endo diastereomer = 7.51 – 7.42 (m, 2 H), 7.29 (d, ^3J = 6.8 Hz, 1 H), 7.14 – 7.02 (m, 3 H), 6.78 (td, ^3J = 7.5 Hz, ^4J = 1.2 Hz, 1 H), 6.61 (dd, ^3J = 8.0 Hz, ^4J = 1.1 Hz, 1 H), 5.22 (d, ^3J = 8.0 Hz, 1 H), 4.68 (d, J = 3.0 Hz, 1 H), 3.87 (s, 1 H), 3.79 – 3.58 (m, 2 H), 2.74 (tdd, ^3J = 10.0 Hz, ^4J = 5.6 Hz, ^4J = 3.1 Hz, 1 H), 2.17 – 2.05 (m, 1 H), 1.50 – 1.41 (m, 1 H).

δ [ppm] exo diastereomer = 7.48 – 7.40 (m, 2 H), 7.34 (dd, ^3J = 7.6, ^4J = 1.5 Hz, 1 H), 7.15 – 7.04 (m, 3 H), 6.80 – 6.73 (m, 1 H), 6.63 (dd, ^3J = 8.1 Hz, ^4J = 1.1 Hz, 1 H), 4.55 (d, ^3J = 5.1 Hz, 1 H), 4.17 (s, 1 H), 4.03 – 3.93 (m, 1 H), 3.83 – 3.74 (m, 2 H), 2.45 – 2.34 (m, 1 H), 2.06 – 1.91 (m, 1 H), 1.70 – 1.60 (m, 1 H).

^{13}C NMR (101 MHz, DCM-*d*$_2$):

δ [ppm] endo diastereomer = 162.7 (d, $^{1}J_{C\text{-}F}$ = 244.9 Hz, ***C***F), 145.6, 138.9, 130.7, 128.7 (d, $J_{C\text{-}F}$ = 7.9 Hz, CF-***C***H), 123.5, 119.6, 116.0, 115.7, 115.5, 76.4, 67.2, 57.5, 46.4, 25.3.

δ [ppm] exo diastereomer = 163.1 (d, $^{1}J_{C\text{-}F}$ = 245.4 Hz), 138.4, 131.9, 130.5 (d, $^{3}J_{C\text{-}F}$ = 8.1 Hz), 129.3, 128.8, 121.0, 118.7, 115.9 (d, $^{2}J_{C\text{-}F}$ = 21.4 Hz), 115.2, 76.6, 65.6, 57.7, 44.1, 29.3.

^{19}F NMR (235 MHz, DCM-*d*$_2$):

δ [ppm] endo diastereomer = -115.8 – -115.9 (m)

δ [ppm] exo diastereomer = -115.1 – -115.3 (m)

ESI-MS:

endo diastereomer *m*/*z* (+) = calc. 270.1289 $[M+H]^+$; found 270.1280 $[M+H]^+$.

exo diastereomer *m*/*z* (+) = calc. 270.1289 $[M+H]^+$; found 270.1286 $[M+H]^+$.

3.4.4 Synthesis of (+/-)-9

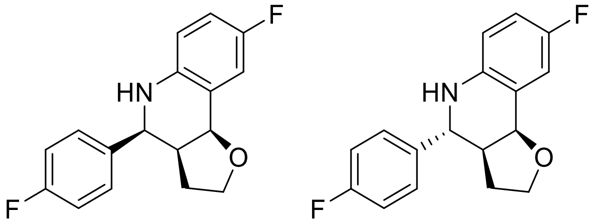

endo exo

For characterisation, compounds **(+/-)-9** were synthesized after the general asymmetric catalysis procedure for the Povarov [4+2] cycloaddition (see manuscript) at RT. The diastereomers could not be separated by column chromatography, but can be distinguished by ^{1}H NMR.

R*f* = endo:exo mixture 0.52 (Pentane:EtOAc 5:1)

^{1}H NMR (300 MHz, DCM-*d*$_2$):

δ [ppm] endo:exo mixture = 7.50 – 7.39 (m, 2 H), 7.15 – 6.99 (m, 3 H), 6.90 – 6.76 (m, 1 H), 6.59 (ddd, ^{3}J = 8.8 Hz, ^{3}J = 6.9 Hz, ^{3}J = 4.7 Hz, 1 H), 5.18 (d, ^{3}J = 7.9 Hz, 1 H_{endo}), 4.66 (d, ^{3}J = 3.0 Hz, 1 H_{endo}), 4.52 (d, ^{3}J = 5.3 Hz, 1 H_{exo}), 4.15 – 4.03 (m, 2 H), 3.98 (td, J = 8.4, 5.9 Hz, 1 H), 3.85 – 3.62 (m, 3 H), 2.80 – 2.65 (m, 1 H_{endo}), 2.42 (dddd, ^{3}J

= 10.8 Hz, ^3J = 7.9 Hz, ^3J = 5.3 Hz, ^4J = 2.4 Hz, 1 H_{exo}), 2.20 – 1.94 (m, 3 H_{endo}), 1.70 – 1.58 (m, 1 H_{exo}), 1.50 – 1.42 (m, 1 H_{endo}).

^{13}C NMR (101 MHz, DCM-*d*$_2$):

δ [ppm] endo diastereomer = 162.8 (d, $^1J_{C-F}$ = 244.9 Hz, endo), 157.1 (d, $^1J_{C-F}$ = 235.9 Hz, endo), 141.83 (d, J = 1.8 Hz), 138.67 (d, J = 3.1 Hz), 128.71 (d, J = 8.0 Hz), 124.71 (d, J = 6.2 Hz), 117.2, 116.45 (d, J = 2.5 Hz), 116.16 (d, J = 2.4 Hz), 115.88 (d, J = 21.4 Hz), 115.9, 76.2, 67.3, 57.5, 46.1, 25.0.

δ [ppm] exo diastereomer = 163.1 (d, $^1J_{C-F}$ = 245.5 Hz), 156.5 (d, $^1J_{C-F}$ = 235.4 Hz), 142.49 (d, J = 1.8 Hz), 138.17 (d, J = 3.1 Hz), 130.44 (d, J = 8.1 Hz), 122.30 (d, J = 6.8 Hz), 117.5, 116.38 (d, J = 1.8 Hz), 116.16 (d, J = 2.4 Hz), 115.95 (d, J = 21.2 Hz), 115.6, 76.4, 65.8, 58.1, 44.2, 29.3.

Distinction of the diastereomers is based on the different intensities of the signals, based on the major diasteromere (exo) and minor enantiomer (endo).

^{19}F NMR (235 MHz, DCM-*d*$_2$):

δ [ppm] endo diastereomer = -115.74 (td, J_{H-F} = 8.6 Hz, J_{H-F} = 4.7 Hz), -126.47 (td, J_{H-F} = 8.6 Hz, J_{H-F} = 4.7 Hz), 142.49 (d, J = 1.8 Hz)

δ [ppm] exo diastereomer = -115.06 (td, J_{H-F} = 8.7 Hz, J_{H-F} = 4.4 Hz), -127.43 (td, J = 8.8 Hz, J_{H-F} = 4.7 Hz).

Distinction of the diastereomers is based on the different intensities of the signals, based on the major diasteromere (exo) and minor enantiomer (endo).

ESI-MS:

endo:exo mixture *m*/*z* (+) = calc. 288.1194 $[M+H]^+$; found 288.1185 $[M+H]^+$.

3.4.5 Synthesis of (+/-)-10

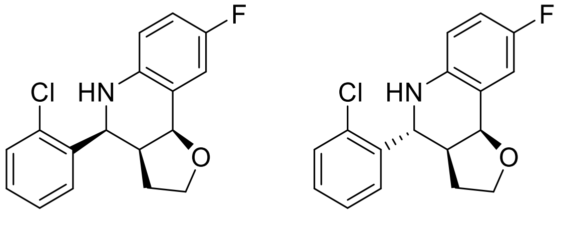

endo exo

For characterisation, compounds **(+/-)-10** were synthesized after the general asymmetric catalysis procedure for the Povarov [4+2] cycloaddition (see manuscript)

at RT. The diastereomers were separated by column chromatography using Pentane:EtOAc 10:1.

R_f = endo diastereomer 0.47, exo diastereomer 0.40 (Pentane:EtOAc 10:1).

^{1}H NMR (300 MHz, DCM-*d_2*):

δ [ppm] endo diastereomer = 7.75 (dd, ^{3}J = 7.7 Hz, ^{4}J = 1.8 Hz, 1 H), 7.41 (dd, ^{3}J = 7.7 Hz, ^{4}J = 1.5 Hz, 1 H), 7.38 – 7.23 (m, 2 H), 7.04 (dd, ^{3}J = 9.3 Hz, ^{3}J = 2.9 Hz, 1 H), 6.81 (td, ^{3}J = 8.7 Hz, ^{4}J = 3.1 Hz, 1 H), 6.60 (dd, ^{3}J = 8.8 Hz, ^{4}J = 4.7 Hz, 1 H), 5.20 (d, ^{3}J = 8.0 Hz, 1 H), 5.08 (d, J = 3.0 Hz, 1 H), 3.82 – 3.64 (m, 3 H), 3.00 (dtt, ^{3}J = 10.6 Hz, ^{3}J = 8.1 Hz, ^{4}J = 2.4 Hz, 1 H), 2.10 (ddt, ^{3}J = 12.1, ^{3}J = 10.7, ^{3}J = 8.9 Hz, 1 H), 1.41 (dddd, ^{3}J = 11.9 Hz, ^{3}J = 8.2 Hz, ^{3}J = 6.7 Hz, ^{4}J = 3.5 Hz, 1 H).

δ [ppm] exo diastereomer = 7.62 (dd, ^{3}J = 7.7 Hz, ^{4}J = 1.9 Hz, 1 H), 7.43 (dd, ^{3}J = 7.8 Hz, ^{4}J = 1.4 Hz, 1 H), 7.39 – 7.24 (m, 2 H), 7.08 (dd, ^{3}J = 9.1 Hz, ^{4}J = 3.0 Hz, 1 H), 6.86 (td, ^{3}J = 8.6 Hz, ^{4}J = 2.9 Hz, 1 H), 6.60 (dd, ^{3}J = 8.8 Hz, ^{4}J = 4.7 Hz, 1 H), 4.57 (d, ^{3}J = 5.2 Hz, 1 H), 4.45 (d, ^{3}J = 10.7 Hz, 1 H), 4.13 – 3.97 (m, 2 H), 3.83 (ddd, ^{3}J = 9.2 Hz, ^{3}J = 8.4 Hz, ^{3}J = 5.7 Hz, 1 H), 2.52 (dddd, ^{3}J = 10.6 Hz, ^{3}J = 7.8 Hz, ^{3}J = 5.3 Hz, ^{4}J = 2.5 Hz, 1 H), 2.14 – 2.01 (m, 1 H), 1.75 (dddd, ^{3}J = 13.2 Hz, ^{3}J = 8.1 Hz, ^{3}J = 5.6 Hz, ^{3}J = 2.4 Hz, 1 H).

^{13}C NMR (101 MHz, DCM-*d_2*):

δ [ppm] endo diastereomer = 157.2 (d, $^{1}J_{C-F}$ = 236.1 Hz), 142.0, 139.8, 133.1, 130.3, 129.2, 128.03 (d, $^{2}J_{C-F}$ = 44.2 Hz), 124.94 (d, $^{3}J_{C-F}$ = 6.2 Hz), 116.52 (d, $^{3}J_{C-F}$ = 4.9 Hz), 116.43 (t, $^{2}J_{C-F}$ = 34.2 Hz), 115.9, 115.6, 76.0, 67,4, 54.6, 42.3, 25.2.

δ [ppm] exo diastereomer = 156.5 (d, $^{1}J_{C-F}$ = 235.5 Hz), 142.4 (d, $^{4}J_{C-F}$ = 1.8 Hz), 139.8, 134.8, 130.1,129.9, 129.6, 129.1, 122.3 (d, ^{3}J = 6.8 Hz), 117.3 (d, ^{2}J = 21.8 Hz), 116.3 (d, ^{2}J = 13.0 Hz), 116.1 (d, ^{3}J = 2.4 Hz), 76.2, 66.2, 53.5, 44.1, 29.2.

^{19}F NMR (235 MHz, DCM-*d_2*):

δ [ppm] endo diastereomer = -126.3 (td, J = 8.8 Hz, J = 4.7 Hz).

δ [ppm] exo diastereomer = -127.4 (td, J = 8.7 Hz, J = 4.7 Hz).

ESI-MS:

endo diastereomer *m/z* (+) = calc. 304.0899 $[M+H]^+$; found 304.0884 $[M+H]^+$.

exo diastereomer *m/z* (+) = calc. 304.0899 $[M+H]^+$; found 304.0895 $[M+H]^+$.

3.4.6 Synthesis of (+/-)-11

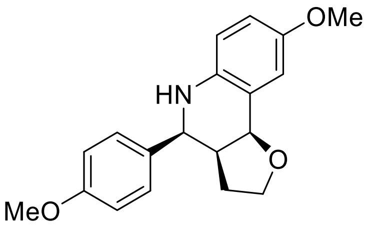

endo

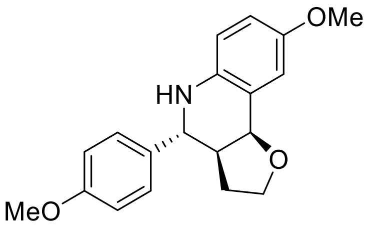

exo

For characterisation, compounds **(+/-)-11** were synthesized after the general asymmetric catalysis procedure for the Povarov [4+2] cycloaddition (see manuscript) at RT. The diastereomers were separated by column chromatography using Pentane:EtOAc 2:1.

$\mathbf{R_f}$ = endo diastereomer 0.61, exo diastereomer 0.53 (Pentane:EtOAc 2:1).

^{1}H NMR (300 MHz, DCM-d_2):

δ [ppm] endo diastereomer = 7.39 (dd, ^3J = 8.3 Hz, ^4J = 1.6 Hz, 2 H), 6.91 (dd, ^3J = 8.7 Hz, ^3J = 2.1 Hz, 2 H), 6.87 (d, ^3J = 2.9 Hz, 1 H), 6.68 (dd, ^3J = 8.8 Hz, ^4J = 3.0 Hz, 1 H), 6.55 (d, ^3J = 8.7 Hz, 1 H), 5.18 (d, ^3J = 8.0 Hz, 1 H), 4.56 (d, ^3J = 3.0 Hz, 1 H), 3.81 (s, 3 H), 3.76 – 3.72 (m, 4 H), 3.70 – 3.62 (m, 2 H), 2.73 (dtd, ^3J = 11.0 Hz, ^3J = 8.1 Hz, ^4J = 3.0 Hz, 1H), 2.15 (ddt, ^3J = 12.1, ^3J = 10.4, ^3J = 8.8 Hz, 1 H), 1.57 – 1.42 (m, 1 H).

δ [ppm] exo diastereomer = 7.36 (dd, ^3J = 8.6 Hz, ^4J = 2.0 Hz, 2 H), 6.95 – 6.88 (m, 3 H), 6.72 (dd, ^3J = 8.7 Hz, ^4J = 2.9 Hz, 1 H), 6.57 (d, ^3J = 8.7 Hz, 1 H), 4.53 (d, ^3J = 5.4 Hz, 1 H), 3.96 (td, ^3J = 8.4 Hz, ^4J = 5.9 Hz, 2 H), 3.81 (s, 3 H), 3.78 – 3.72 (m, 4 H), 2.42 (dddd, ^3J = 10.9 Hz, ^3J = 7.9 Hz, ^3J = 5.4 Hz, ^4J = 2.4 Hz, 1 H), 1.98 (dddd, ^3J = 13.0 Hz, ^3J = 9.1 Hz, ^3J = 8.1 Hz, ^3J = 5.9 Hz, 1 H), 1.65 (dddd, ^3J = 13.0 Hz, ^3J = 8.5 Hz, ^3J = 6.3 Hz, ^4J = 2.3 Hz, 1 H).

^{13}C NMR (101 MHz, DCM-d_2):

δ [ppm] endo diastereomer = 159.6, 153.6, 139.9, 129.2, 128.2, 124.4, 116.5, 115.9, 114.6, 114.4, 76.8, 67.3, 57.9, 56.2, 55.8, 46.6, 25.2.

δ [ppm] exo diastereomer = 160.1, 153.0, 140.5, 134.6, 129.9, 122.0, 116.5, 116.2, 115.7, 114.4, 77.0, 65.8, 58.4, 56.3, 55.8, 44.3, 29.5.

ESI-MS:

m/*z* (-) = calc. 312.1585 [M+H]$^+$; found 312.1584 [M+H]$^+$.

m/*z* (-) = calc. 312.1585 [M+H]$^+$; found 312.1584 [M+H]$^+$.

3.4.7 Synthesis of (+/-)-12

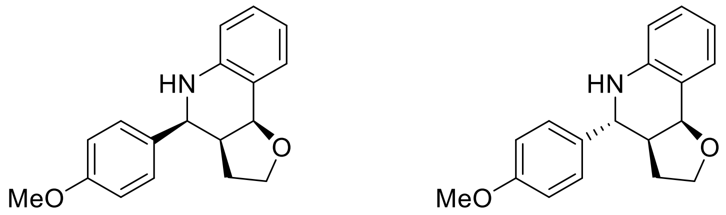

endo exo

Compound **(+/-)-12** was synthesized after the general NMR scale procedure for the Povarov [4+2] cycloaddition (see manuscript). The spectroscopic data are in agreement with literature data.[7b]

4 Catalysis experiments

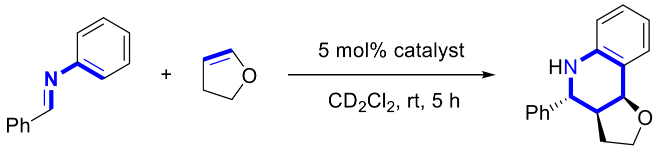

1 **2** **(+/-)-3**

Scheme 1: Povarov [4+2] cycloaddition of N-benzylideneaniline (**1**) and two equivalents of 2,3-dihydrofuran (DHF) (**2**) in presence of 5 mol% catalyst.

4.1 Yield determination for NMR scale Povarov [4+2] cycloaddition

The yield of the corresponding product **(+/-)-3** was determined as follows: The tetraethylsilane quartet was integrated and set to 1. Afterwards, the integrals of the characteristic signals for product **(+/-)-3** at 5.21 ppm (*endo* diastereomer) and 4.55 ppm (*exo* diastereomer) as well as the N=C*H* signal of **1** at 8.48 ppm and the formed benzaldehyde at 10.02 ppm were added. Afterwards, 100 was divided by this sum. This value was multiplied by the sum of the integrals for product **(+/-)-3**, giving the overall yield of **(+/-)-3** (Figure S1 and Figure S2).

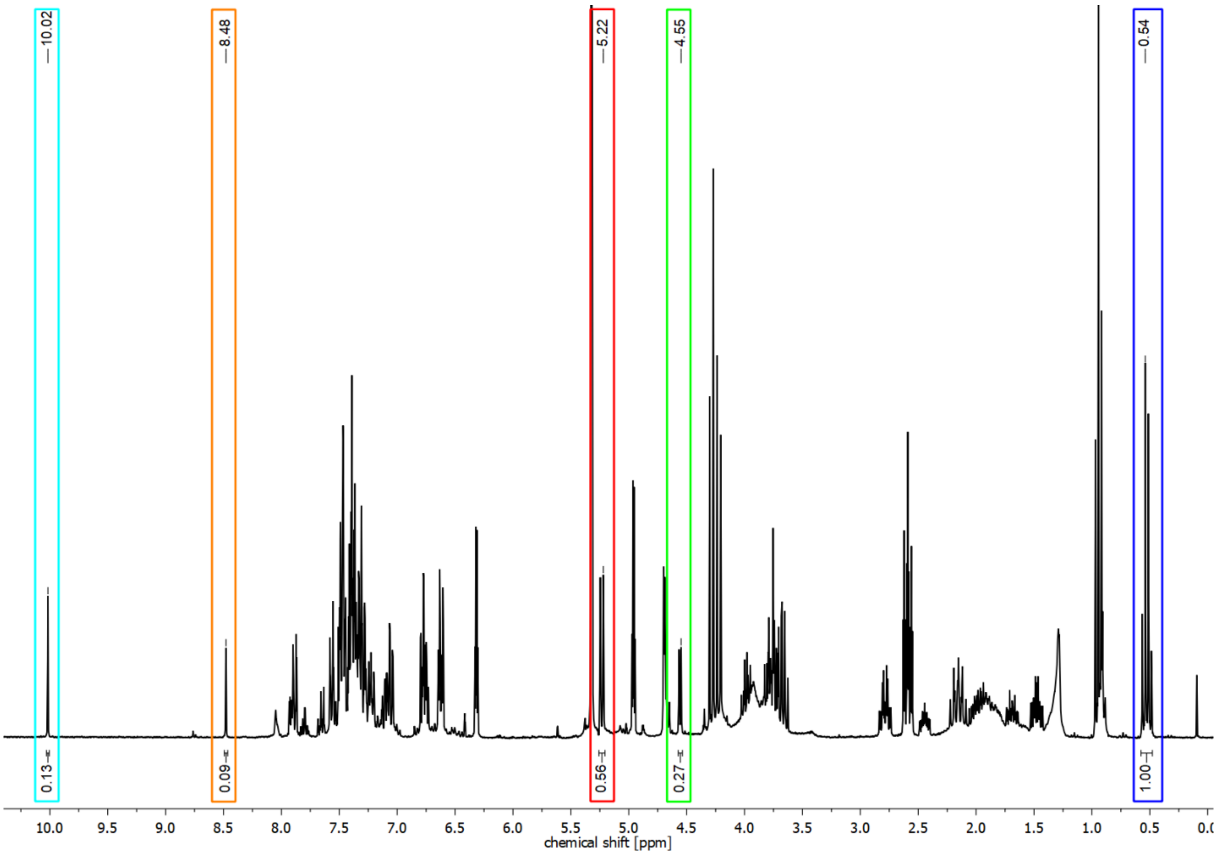

Figure S1: Example of a ^{1}H NMR spectrum of Povarov [4+2] cycloaddition after 5 hours reaction time in the presence of $\mathbf{4}^{Te\text{-}BF_4}$.The tetraethylsilane quartet at 0.54 ppm is highlighted in blue. The signals for product **3** are highlighted in red at 5.22 ppm for endo-**3** and in green at 4.55 ppm for exo-**3.** The signal for the formed benzaldehyde at 10.02 ppm is marked in cyan and the signal for starting material **1** is colored in orange at 8.48 ppm.

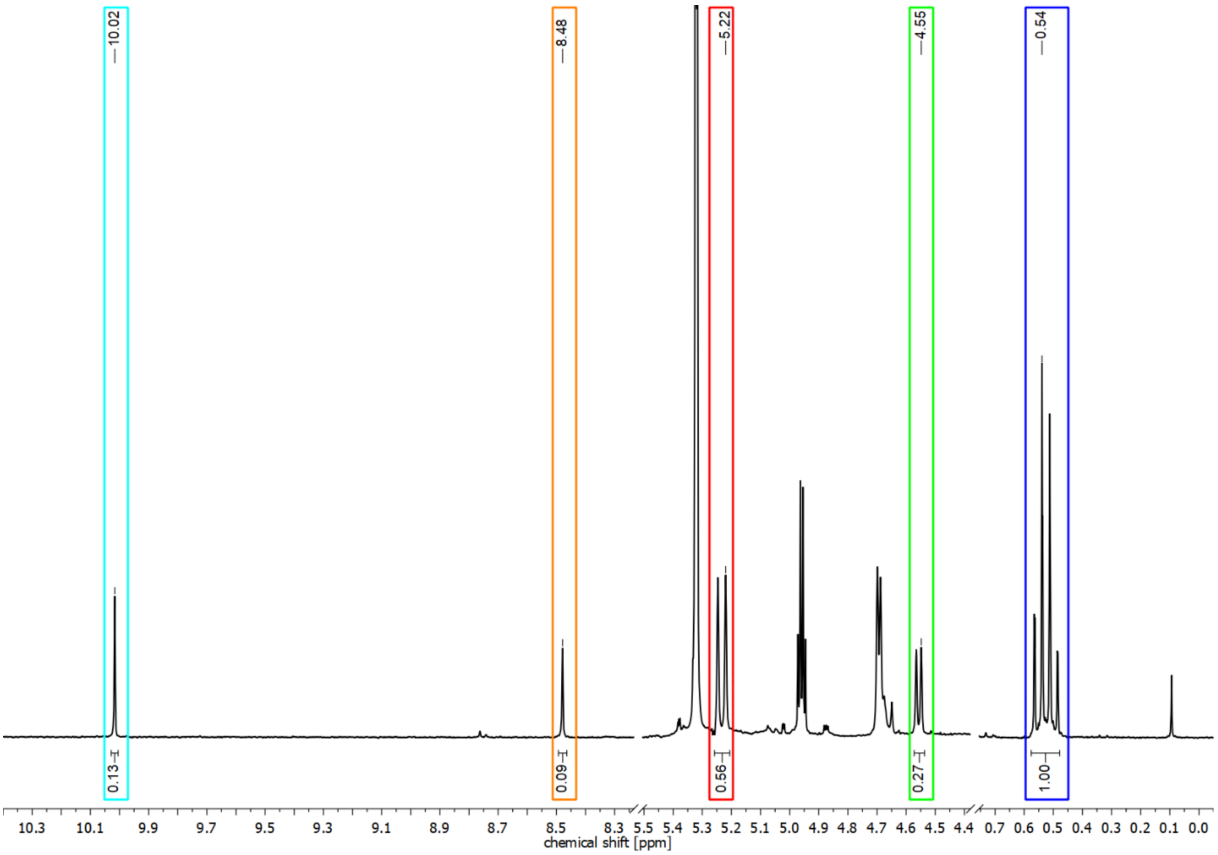

Figure S2: Enlargement of the previously shown spectra shown in Figure S1. The region of 8.25 ppm to 5.50 ppm and 0.75 ppm to 4.40 ppm has been cut for clarity.

4.2 Determination of TOF numbers

The turnover frequencies (TOF) were determined by the following equation.

$$\frac{n_{Product}\ [mol]/t\ [h]}{n_{cat}\ [mol]} = TOF\ [h^{-1}]$$

The yield after 20 minutes reaction time (t = 0.33) was used for the determination of the amount of product **(+/-)-3** (Table S1). The time vs. yield plot is shown in Figure S3.

Table S1: Determination of the TOF numbers. The yield of **(+/-)-3** after 20 minutes in presence of 5 mol% catalyst was used.

Entry	Catalyst	Yield [%][a]	Amount of **(+/-)-3** (μmol)	TOF [h^{-1}]
1	$\mathbf{4}^{\mathbf{Te\text{-}BArF_4}}$	60	17.8	36
2	$\mathbf{4}^{\mathbf{Te\text{-}BF_4}}$	29	8.58	17
3	$\mathbf{4}^{\mathbf{Te\text{-}OTf}}$	18	5.37	11
4	$\mathbf{4}^{\mathbf{Se\text{-}BArF_4}}$	4	1.09	2.2
5	$\mathbf{4}^{\mathbf{Se\text{-}BF_4}}$	5	1.35	2.7
6	$\mathbf{4}^{\mathbf{Se\text{-}OTf}}$	12	3.66	7.4
7	$\mathbf{4}^{\mathbf{S\text{-}OTf}}$	15	4.34	8.8

[a] Determined by ^{1}H NMR spectroscopy. A measuring error of 5% is assumed.

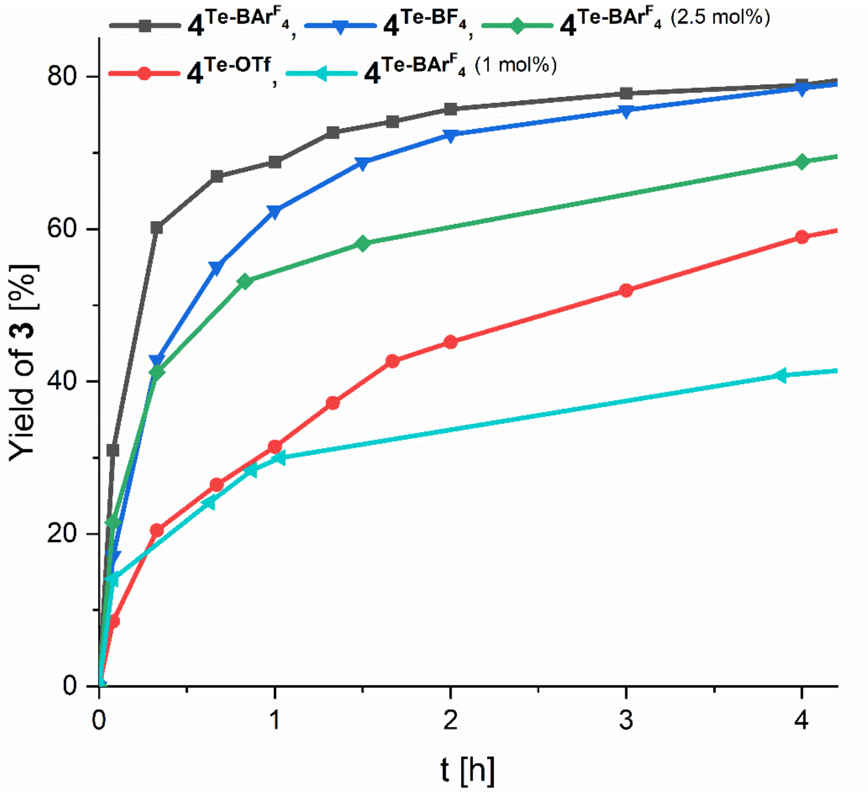

Figure S3: Yield versus time plot of the Povarov [4+2] cycloaddition between **1** and **2** catalyzed by chalcogen bonding catalysts $\mathbf{4}^{\mathbf{Te\text{-}BArF_4}}$, $\mathbf{4}^{\mathbf{Te\text{-}BF_4}}$, and $\mathbf{4}^{\mathbf{Te\text{-}OTf}}$.

4.3 Visual Kinetic Analysis

With the help of visual kinetic analysis,[8] the catalyst order in product **(+/-)-3** and in starting material **1** was determined. The reaction order with catalyst $\mathbf{4}^{\mathbf{Te\text{-}BArF_4}}$ was determined to be second order. The excel sheet provided by the authors was used for the analysis. t[cat] indicates the value of the normalized timescale for the concentration of the employed catalyst. t[cat] is usually giving without a unit and is calculated by the known concentration of the catalyst and reaction time as follows:

$$t[cat]_i = t[cat]_{i-1} + \left(\frac{[cat]_i + [cat]_{i-1}}{2}\right)^{order} * (t_i - t_{i-1})$$

Table S2: Data set for the visual kinetic analysis to determine the order of the catalyst in product **3**, using 2.5 mol% and 5 mol% of $\mathbf{4}^{\mathbf{Te\text{-}BArF_4}}$.

Experiment with 2.5 mol% $\mathbf{4}^{\mathbf{Te\text{-}BArF_4}}$			
$[A]_0$	$[Imine]_0$	0.0598 M	
$[B]_0$	$[DHF]_0$	0.1196 M	
$[Cat]_0$	$[4^{Te\text{-}BArF_4}]_0$	0.0015 M	
t [h]	t[cat]	[P] [M]	[cat] [M]
0.00	0.00	0.00000	0.0015
0.08	1,80E-07	0.01287	0.0015
0.33	7,43E-07	0.02463	0.0015
0.83	1,87E-06	0.03177	0.0015
1.50	3,38E-06	0.03474	0.0015
4.00	9,00E-06	0.04116	0.0015
5.00	1,13E-05	0.04324	0.0015
6.00	1,35E-05	0.04658	0.0015

Experiment with 5 mol% $\mathbf{4}^{\mathbf{Te\text{-}BArF_4}}$			
$[A]_0$	$[Imine]_0$	0.0598 M	
$[B]_0$	$[DHF]_0$	0.1196 M	
$[Cat]_0$	$[4^{Te\text{-}BArF_4}]_0$	0.003 M	
t [h]	t[cat]	[P] [M]	[cat] [M]
0.00	0.00	0.00000	0.003
0.008	7,20E-07	0.01852	0.003
0.33	2,97E-06	0.03601	0.003
0.67	6,03E-06	0.04000	0.003
1.00	9,00E-06	0.04113	0.003
1.33	1,20E-05	0.04370	0.003
1.67	1,50E-05	0.04432	0.003
2.00	1,80E-05	0.04530	0.003
3.00	2,70E-05	0.04651	0.003
4.00	3,60E-05	0.04706	0.003
5.00	4,50E-05	0.04811	0.003
6.00	5,40E-05	0.04946	0.0033

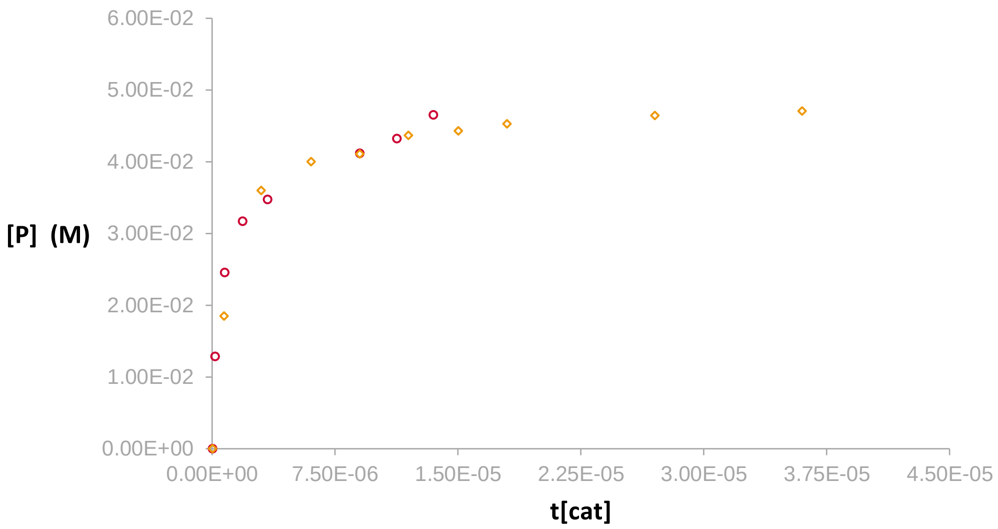

Figure S4: Plot of the corresponding visual kinetic analysis with using 2.5 mol% and 5 mol% of $\mathbf{4}^{\text{Te-BArF}_4}$ for the order of catalyst in product **3**.

Table S3: Data set for the visual kinetic analysis to determine the order of the catalyst in starting material **1**, using 2.5 mol% and 5 mol% of $\mathbf{4}^{\text{Te-BArF}_4}$.

Experiment with 2.5 mol% $\mathbf{4}^{\text{Te-BArF}_4}$			
$[A]_0$	$[Imine]_0$	0.0598 M	
$[B]_0$	$[DHF]_0$	0.1196 M	
$[Cat]_0$	$[4^{\text{Te-BArF}_4}]_0$	0.0015 M	
t [h]	t[cat]	[Imine] [M]	[cat] [M]
0.00	0.00	0,0598	0.0015
0.08	1,80E-07	0,0469	0.0015
0.33	7,43E-07	0,0351	0.0015
0.83	1,87E-06	0,0280	0.0015
1.50	3,38E-06	0,0251	0.0015
4.00	9,00E-06	0,0186	0.0015
5.00	1,13E-05	0,0166	0.0015
6.00	1,35E-05	0,0132	0.0015

Experiment with 5 mol% $\mathbf{4}^{\text{Te-BArF}_4}$			
$[A]_0$	$[Imine]_0$	0.0598 M	
$[B]_0$	$[DHF]_0$	0.1196 M	
$[Cat]_0$	$[4^{\text{Te-BArF}_4}]_0$	0.003 M	
t [h]	t[cat]	[Imine] [M]	[cat] [M]
0.00	0.00	0,0598	0.003
0.008	7,20E-07	0,0413	0.003
0.33	2,97E-06	0,0238	0.003
0.67	6,03E-06	0,0198	0.003
1.00	9,00E-06	0,0187	0.003
1.33	1,20E-05	0,0163	0.003
1.67	1,50E-05	0,0155	0.003
2.00	1,80E-05	0,0145	0.003
3.00	2,70E-05	0,0133	0.003
4.00	3,60E-05	0,1274	0.003
5.00	4,50E-05	0,0108	0.003
6.00	5,40E-05	0,0103	0.0033

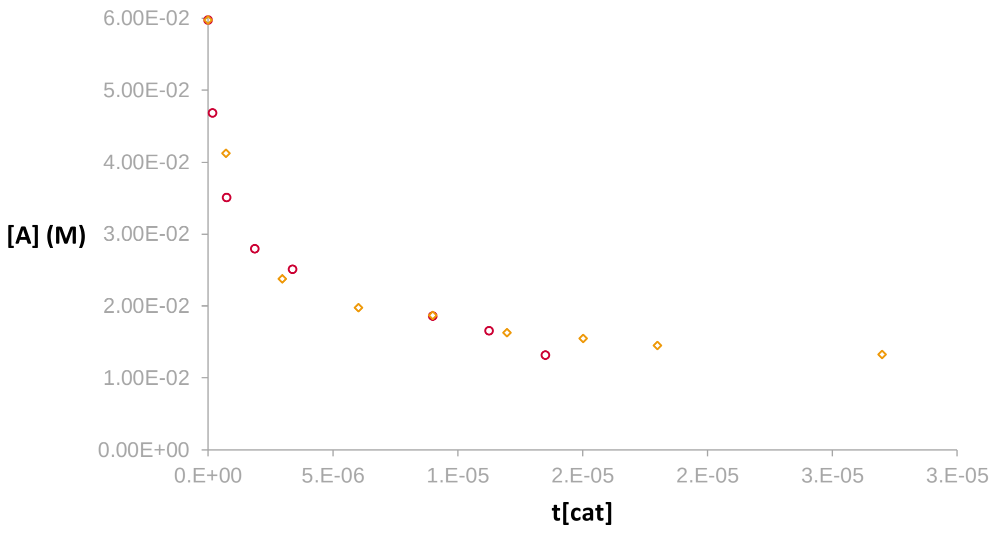

Figure S5: Plot of the corresponding visual kinetic analysis with using 2.5 mol% and 5 mol% of **4**$^{\text{Te-BArF}_4}$ for the order of catalyst in imine **1**.

4.4 ^{1}H NMR Experiments on the interaction between imines and $4^{Te\text{-}BArF_4}$ and the catalyst stability

^{1}H NMR experiments were carried out, in order to investigate the interaction between imine **1** and $\mathbf{4}^{Te\text{-}BArF_4}$ and $\mathbf{4}^{Te\text{-}BF_4}$. Downfield shifts have been observed for the N=C*H* proton, which is therefore deshielded due to the interaction of the imine nitrogen with the chalcogen bonding catalyst (1:1, 8.00 mM) (Figure S6). Also, downfield shifts of the imine carbon atom are observed (Figure S7). The signal of the imine carbon overlaps with $BAr^F{}_4$ signals of $\mathbf{4}^{Te\text{-}BArF_4}$ (Figure S8). Also, downfield shifts for the carbon positioned next to the tellurium was observed for $\mathbf{4}^{Te\text{-}BArF_4}$ and $\mathbf{4}^{Te\text{-}BF_4}$ (Figure S7 and Figure S8).

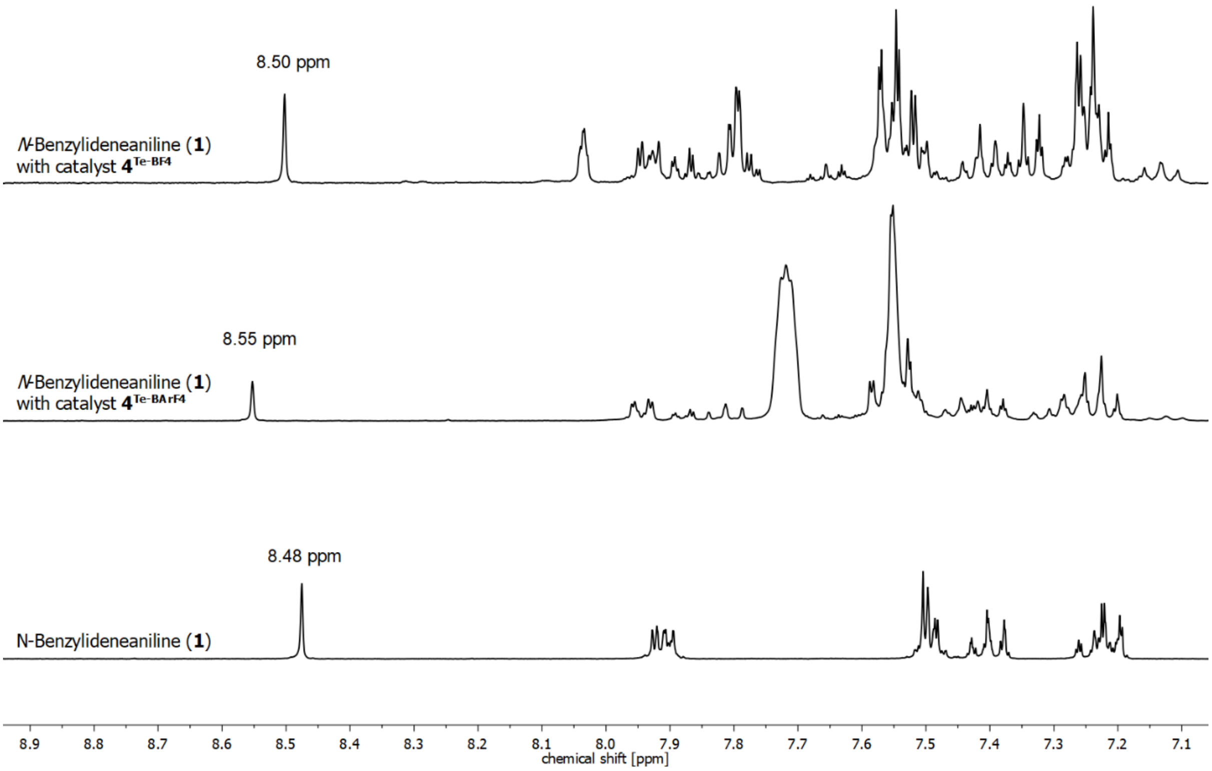

Figure S6: Investigation of the interaction between catalysts $\mathbf{4}^{Te\text{-}BArF_4}/\mathbf{4}^{Te\text{-}BF_4}$ and imine **1** by ^{1}H NMR spectroscopy. The signal for the N=C*H* proton is located at 8.48 ppm without catalyst. With the addition of 1 eq. of $\mathbf{4}^{Te\text{-}BArF_4}$ the signal shifts to 8.55 ppm which shows a deshielding of the proton. With $\mathbf{4}^{Te\text{-}BF_4}$ the signal shifts to 8.50 ppm. Due to overlapping of signals, no changes for signals of $\mathbf{4}^{Te\text{-}BArF_4}/\mathbf{4}^{Te\text{-}BF_4}$ could be obtained. Bottom: ^{1}H NMR spectrum of **1** in DCM-d_2 (8.00 mM). Middle: ^{1}H NMR Spectrum of **1** and $\mathbf{4}^{Te\text{-}BArF_4}$ (1:1) in DCM-d_2 (8.00 mM). Top: ^{1}H NMR Spectrum of **1** and $\mathbf{4}^{Te\text{-}BF_4}$ (1:1) in DCM-d_2 (8.00 mM). Referenced to the solvent DCM-d_2.

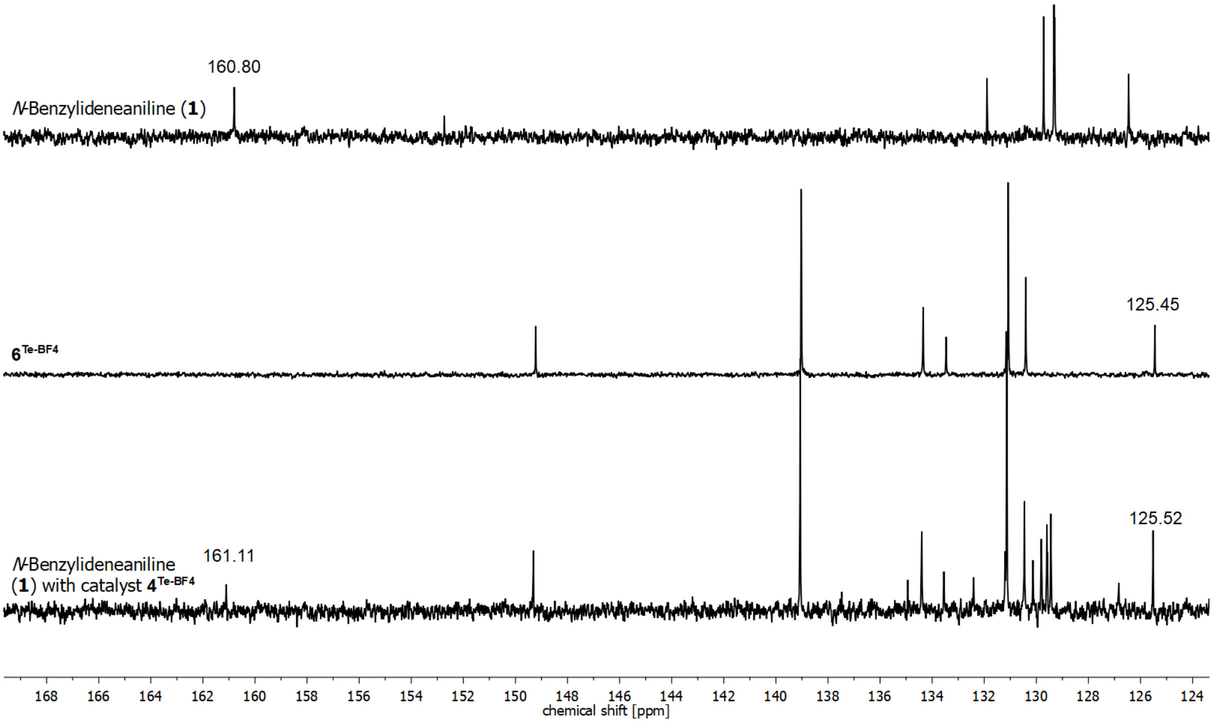

Figure S7: Investigation of the interaction between catalysts $\mathbf{4}^{\mathbf{Te\text{-}BF_4}}$ and imine **1** by ^{13}C NMR spectroscopy. The signal for the N=*C*H carbon is located at 160.80 ppm without catalyst. With the addition of 1 eq. of $\mathbf{4}^{\mathbf{Te\text{-}BF_4}}$ the signal shifts to 161.11 ppm. The carbon functionalized with the tellurium moiety shifts from 125.45 ppm to 125.52 ppm. Bottom: ^{13}C NMR Spectrum of **1** and $\mathbf{4}^{\mathbf{Te\text{-}BF_4}}$ (1:1) in DCM-d_2 (8.00 mM). Middle: ^{13}C NMR Spectrum of $\mathbf{4}^{\mathbf{Te\text{-}BF_4}}$ in DCM-d_2 (8.00 mM). Top: ^{13}C NMR spectrum of **1** in DCM-d_2 (8.00 mM). Referenced to the solvent DCM-d_2.

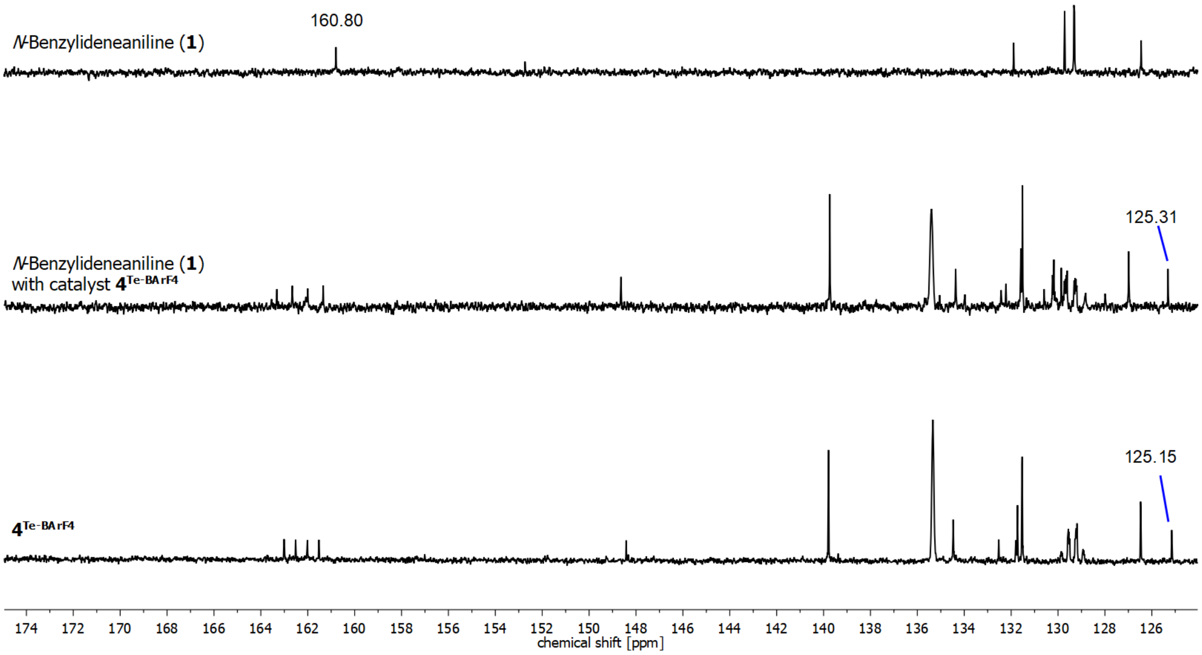

Figure S8: Investigation of the interaction between catalysts $\mathbf{4}^{\mathbf{Te\text{-}BAr^F_4}}$ and imine **1** by ^{13}C NMR spectroscopy. The signal for the N=*C*H carbon is located at 160.80 ppm without catalyst. With the addition of 1 eq. of $\mathbf{4}^{\mathbf{Te\text{-}BAr^F_4}}$ no shifts could be observed, as BAr^F_4 signals overlap with the new imine signal. The carbon functionalized with the tellurium moiety shifts from 125.15 ppm to 125.15 ppm. Bottom: ^{13}C NMR Spectrum of $\mathbf{4}^{\mathbf{Te\text{-}BAr^F_4}}$ in DCM-d_2 (8.00 mM). Middle: ^{13}C NMR Spectrum of **1** and

4$^{Te\text{-}BArF_4}$ (1:1) in DCM-d_2 (8.00 mM). Top: ^{13}C NMR spectrum of **1** in DCM-d_2 (8.00 mM). Referenced to the solvent DCM-d_2.

The ^{1}H NMR experiments were also performed for methoxy substituted imines, because of their slower reactivity (Figure S9 and Figure S10). Downfield shifts of both the N=C*H* protons and the methoxy protons could conclude competitive binding of the imine nitrogen and the methoxy oxygen to the chalcogen bonding donor.

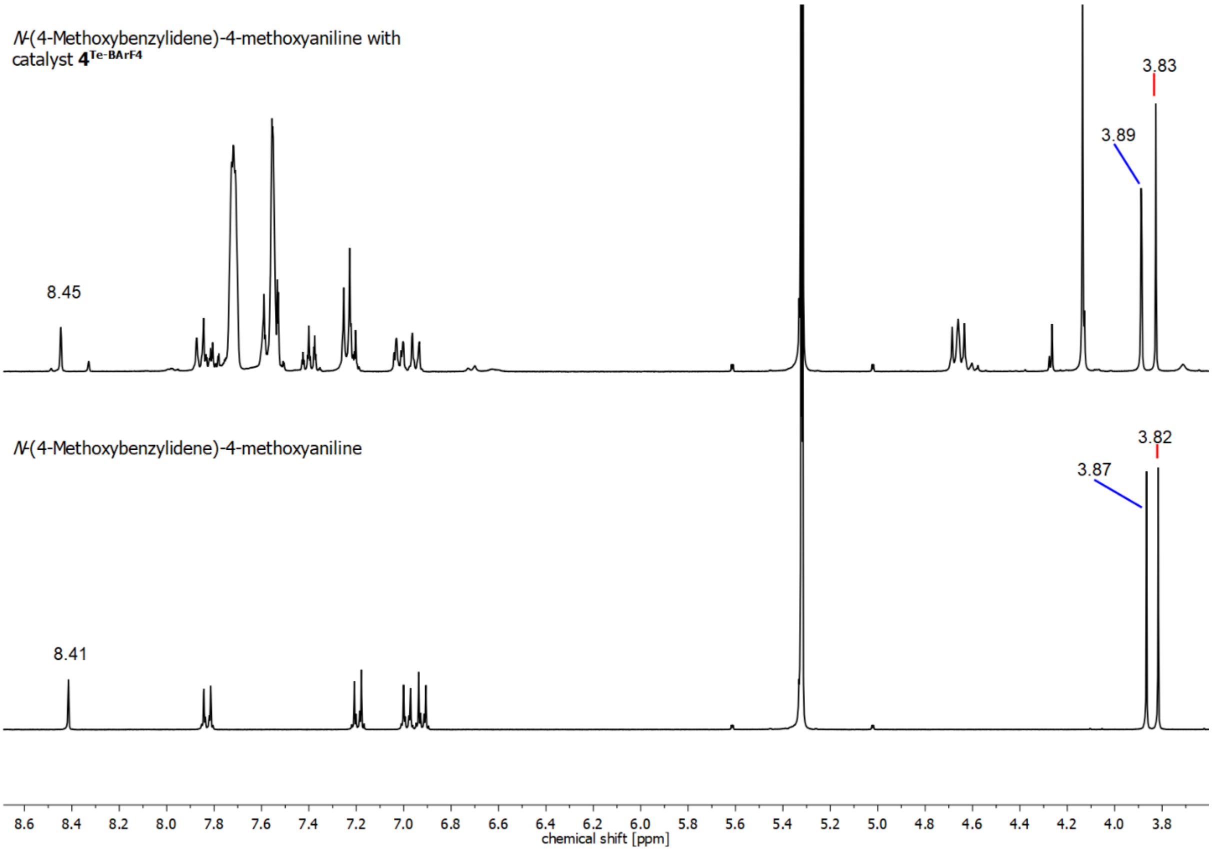

Figure S9: Investigation of the interaction between catalyst **4**$^{Te\text{-}BArF_4}$ and *N*-(4-Methoxybenzylidene)-4-methoxyaniline. The signal for the N=C*H* proton of the corresponding amine is located at 8.41 ppm without **4**$^{Te\text{-}BArF_4}$. With the addition of 1 eq. of **4**$^{Te\text{-}BArF_4}$ the signal shifts to 8.45 ppm which shows a deshielding of the proton. Also, the protons form both methoxy groups (3.87 ppm and 3.82 ppm) are deshielded after the addition of **4**$^{Te\text{-}BArF_4}$. Bottom: ^{1}H NMR Spectrum of *N*-(4-Methoxybenzylidene)-4-methoxyaniline in DCM-d_2 (8.00 mM). Top: S ^{1}H NMR Spectrum of *N*-(4-Methoxybenzylidene)-4-methoxyaniline and **4**$^{Te\text{-}BArF_4}$ in DCM-d_2 (8.00 mM). Referenced to the solvent DCM-d_2.

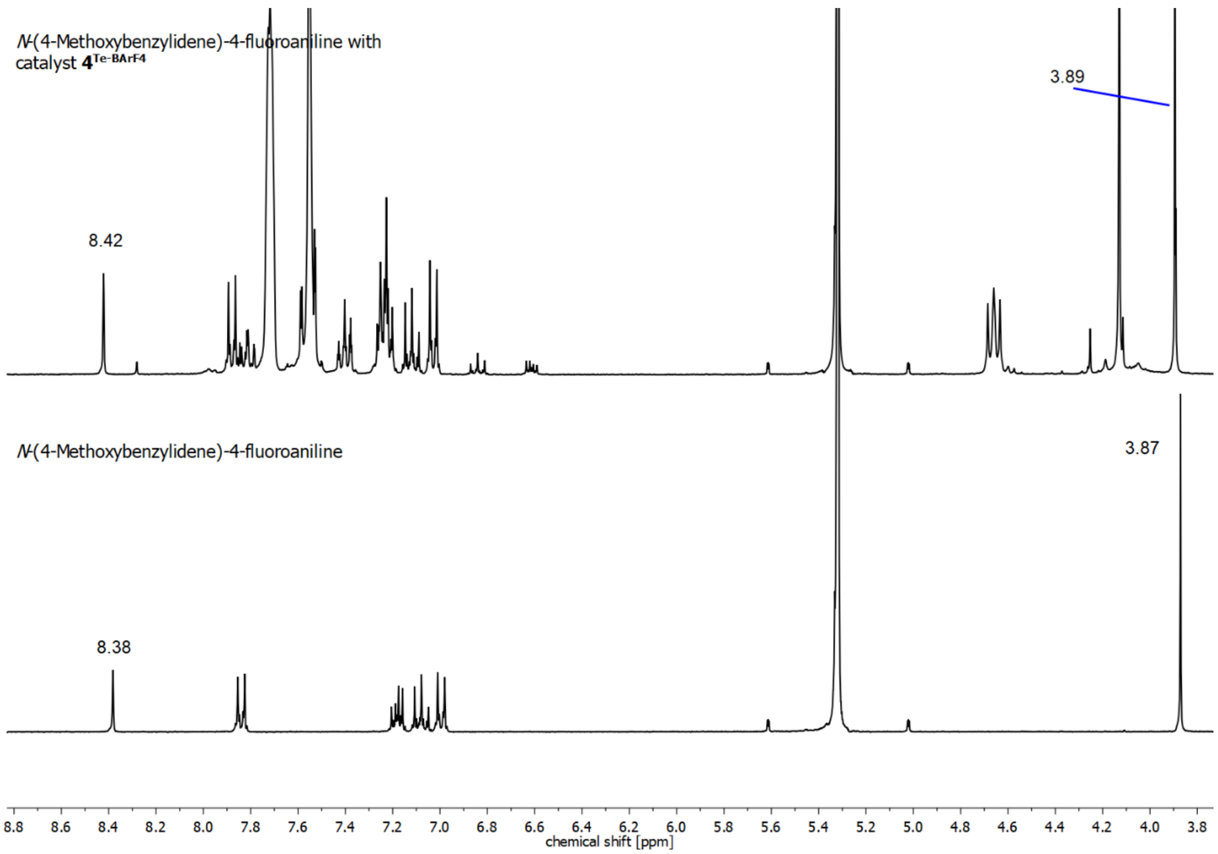

Figure S10: Investigation of the interaction between catalyst **4**$^{\mathbf{Te\text{-}BArF_4}}$ and *N*-(4-Methoxybenzylidene)-4-fluoroaniline. The signal for the N=C*H* proton of the corresponding amine is located at 8.38 ppm without **4**$^{\mathbf{Te\text{-}BArF_4}}$. With the addition of 1 eq. of **4**$^{\mathbf{Te\text{-}BArF_4}}$ the signal shifts to 8.42 ppm which shows a deshielding of the proton. Also, the protons form the methoxy group at 3.87 ppm are deshielded to 3.89 ppm after the addition of **4**$^{\mathbf{Te\text{-}BArF_4}}$. Bottom: ^{1}H NMR Spectrum of *N*-(4-Methoxybenzylidene)-4-fluoroaniline in DCM-d_2 (8.00 mM). Top: S ^{1}H NMR Spectrum *N*-(4-Methoxybenzylidene)-4-fluoroaniline and **4**$^{\mathbf{Te\text{-}BArF_4}}$ in DCM-d_2 (8.00 mM). Referenced to the solvent DCM-d_2.

Furthermore, we determined the decomposition of catalyst **4**$^{\mathbf{Te\text{-}BF_4}}$ to be the respective onefold catalyst **6**$^{\mathbf{BF_4}}$ and the respective hydrogen bonding catalyst **5**$^{\mathbf{BF_4}}$ (Figure S11). For that, a ^{1}H NMR was taken of a reaction mixture after 6 days. Afterwards, the respective onefold catalyst was **6**$^{\mathbf{BF_4}}$ was added and the sample again measured, with an increased signal for the respective triazolium proton, which confirms the decomposition of **4**$^{\mathbf{Te\text{-}BF_4}}$ to **6**$^{\mathbf{BF_4}}$. The same was performed for the hydrogen bonding equivalent **6**$^{\mathbf{BF_4}}$, indicating that after this period of time also this catalyst is formed, as the signal increased.

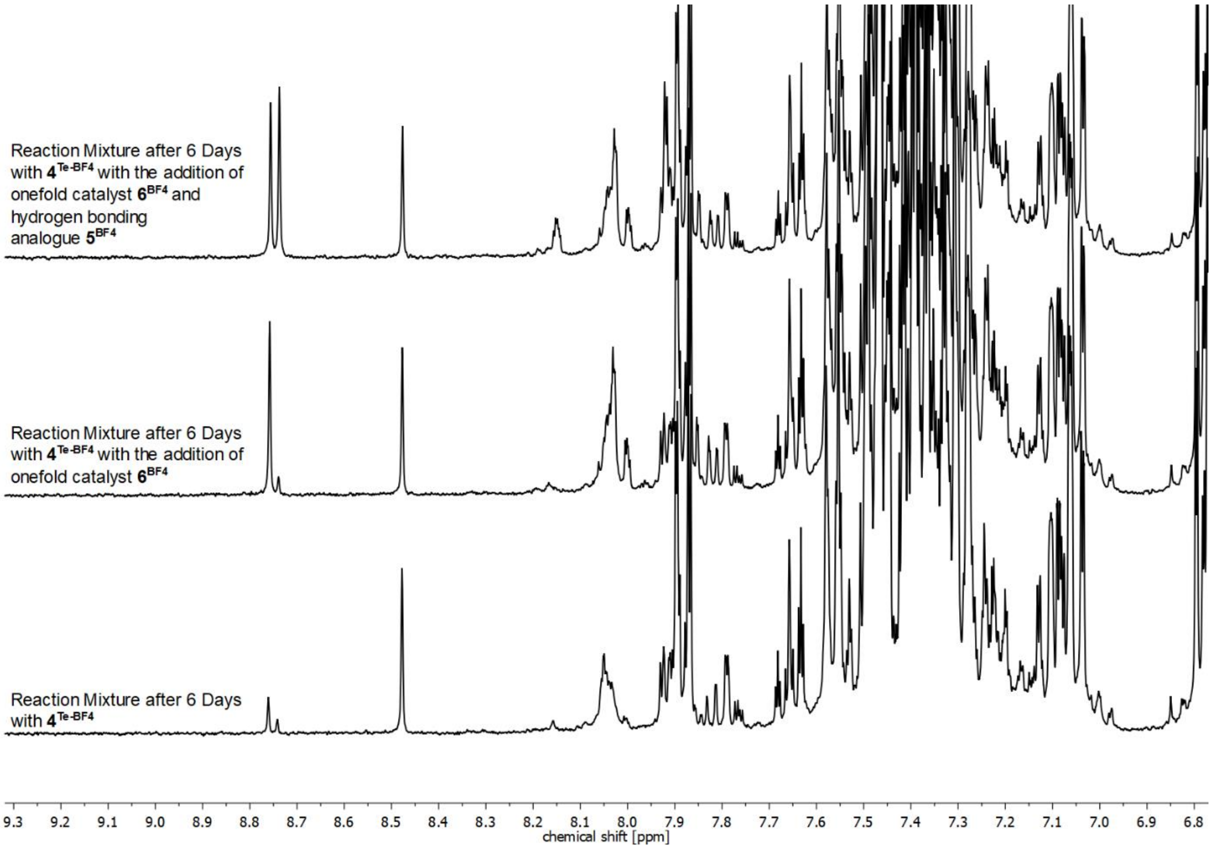

Figure S11: Determination of the decomposition products. The respective onefold catalyst $\mathbf{6^{BF4}}$ and $\mathbf{5^{BF4}}$ were added to a six days old reaction mixture of the Povarov [4+2] cycloaddition with $\mathbf{4^{Te\text{-}BF4}}$. Bottom: Reaction mixture after 6 days. Middle: Addition of $\mathbf{6^{BF4}}$ with an increased signal. Top: Addition of $\mathbf{H^{BF4}}$ with an increased signal.

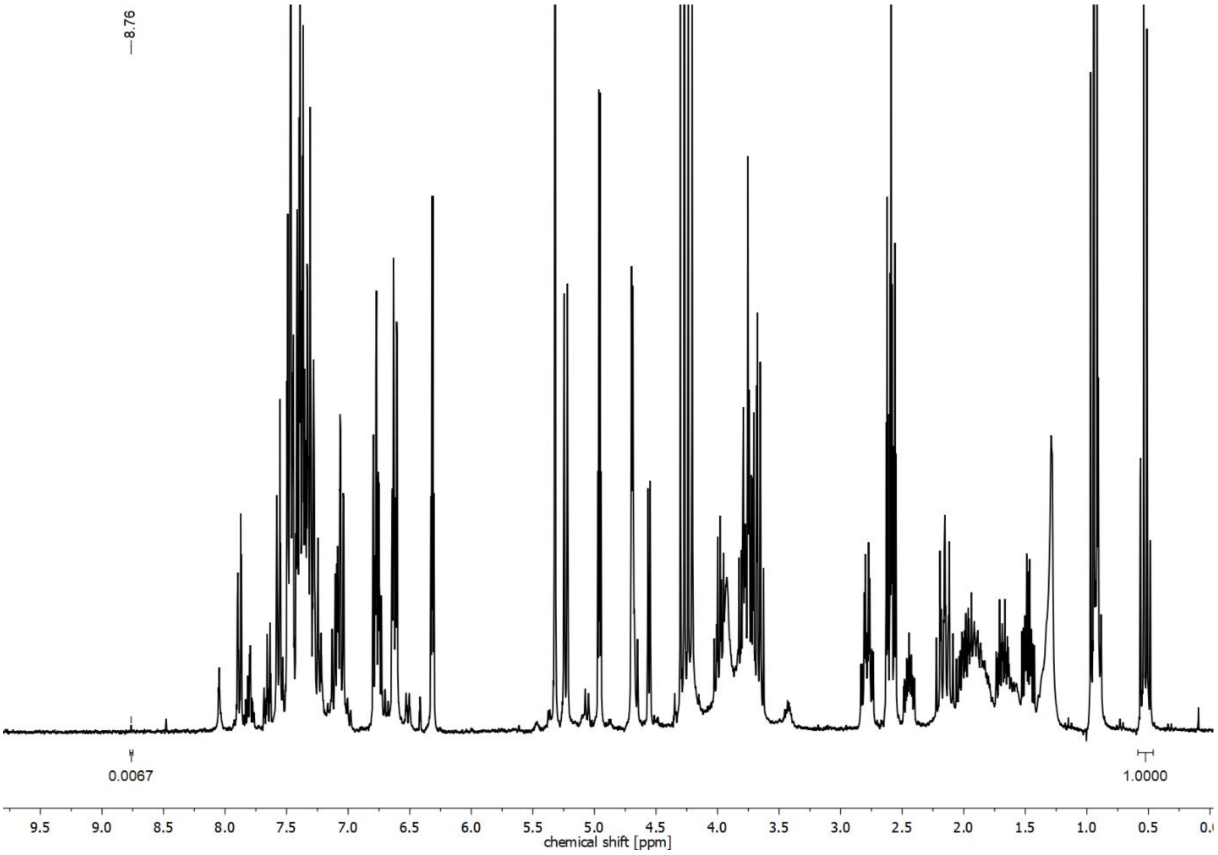

Figure S12: The decomposition was determined by integration of the formed proton signal ($C_{triaz.}$-H, 8.76 ppm) with respect to the internal standard tetraethylsilane. In this example, the integral after 24 h is 0.0067. 100% decomposition to **6$^{Te\text{-}BF_4}$** catalyst would give an integral of 0.05 as 5 mol% catalyst was employed. Therefore, approximately 13% of the catalyst decomposed after 24 h.

5 ^{1}H, ^{13}C, ^{19}F NMR spectra

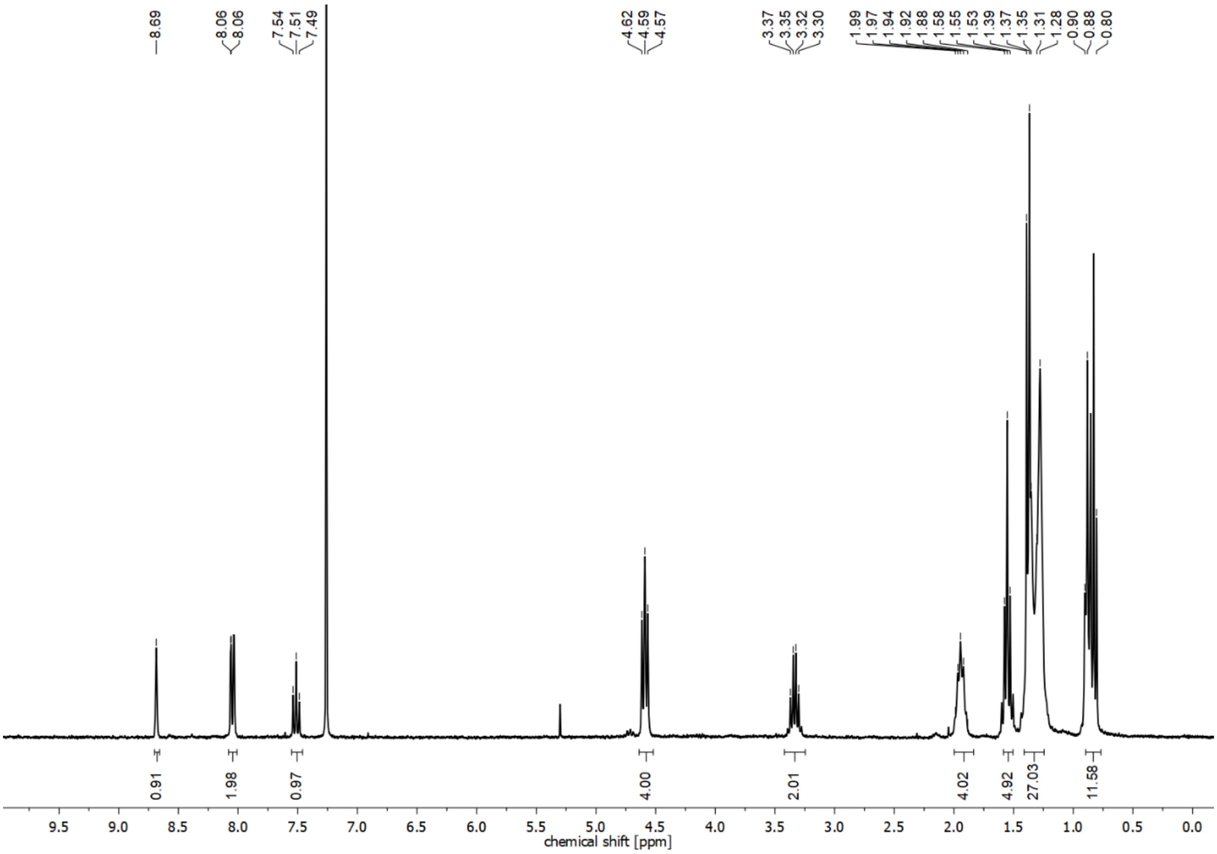

Figure S13: ^{1}H NMR spectrum of $\mathbf{15^{R1}}$ in $CDCl_3$.

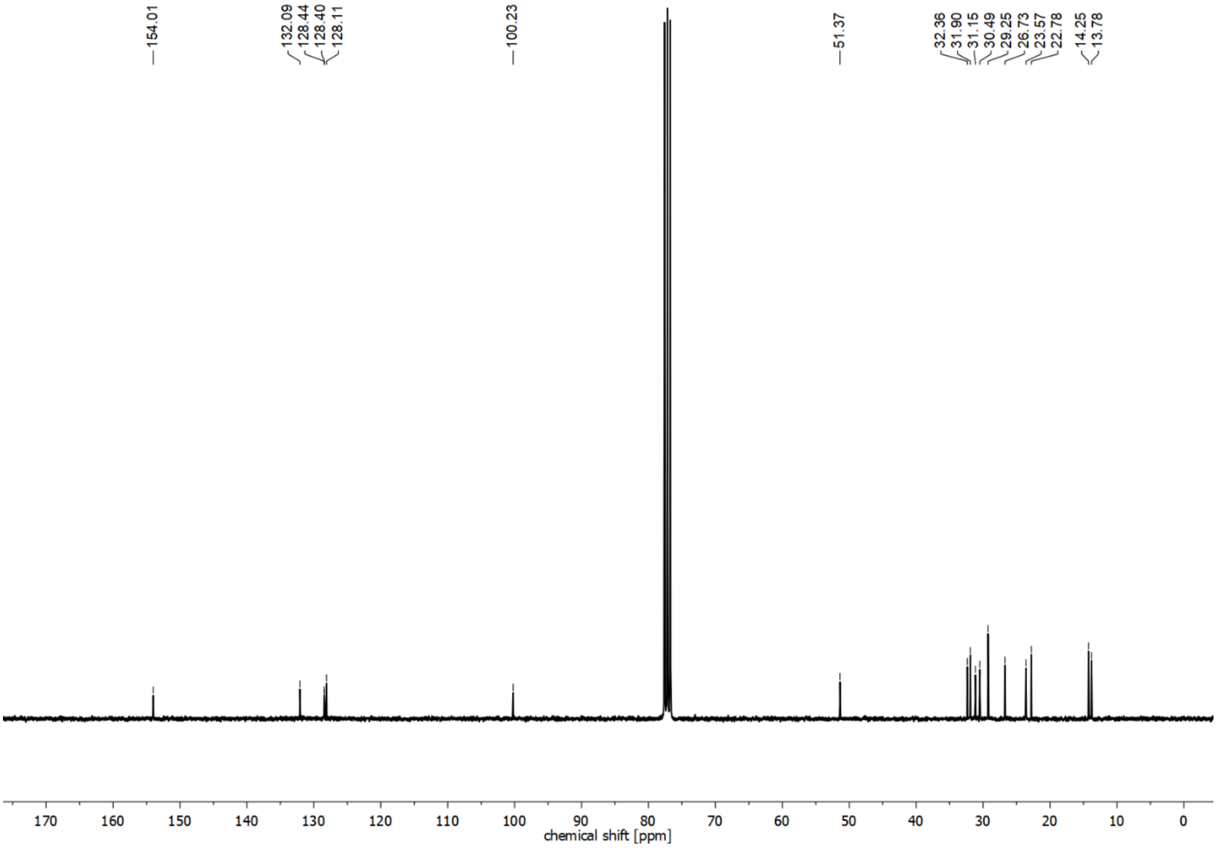

Figure S14: ^{13}C NMR spectrum of **15^{R1}** in $CDCl_3$.

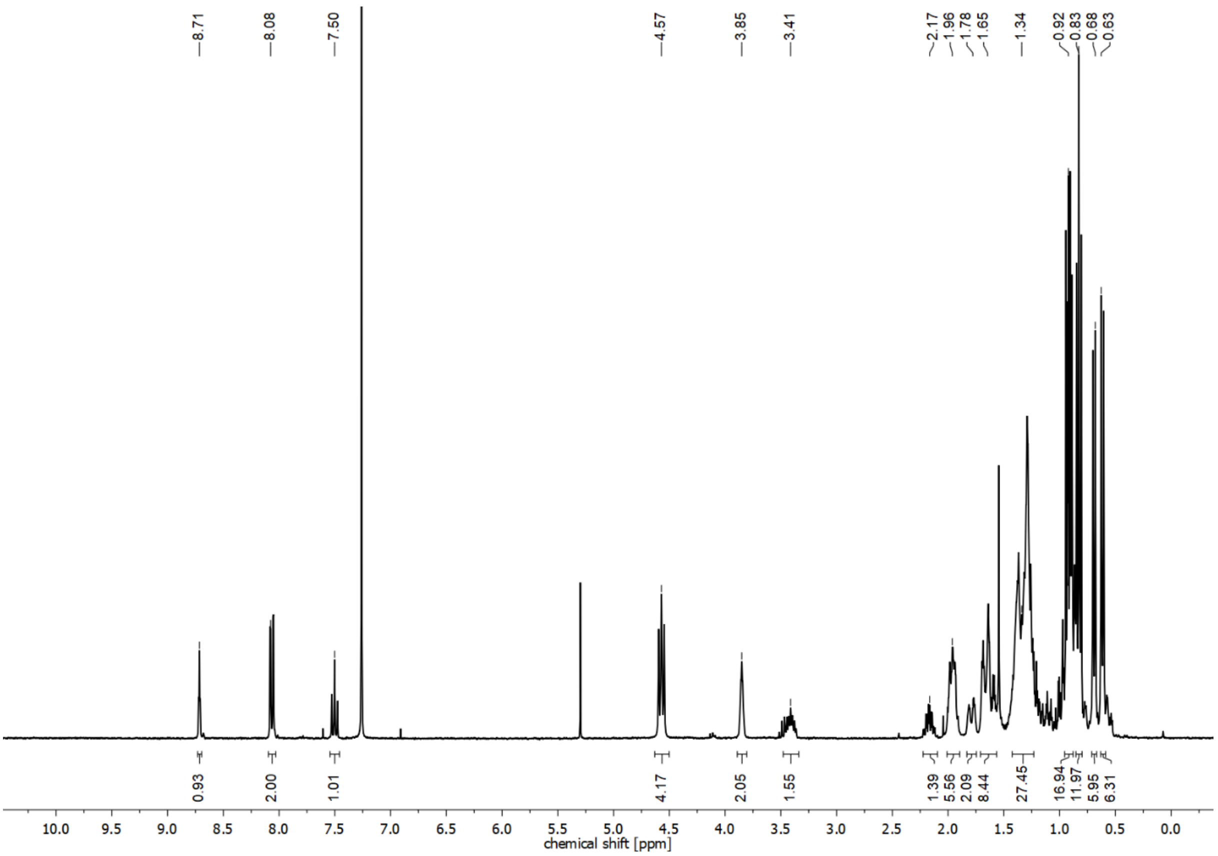

Figure S15: ^{1}H NMR spectrum of **15^{R2}** in $CDCl_3$.

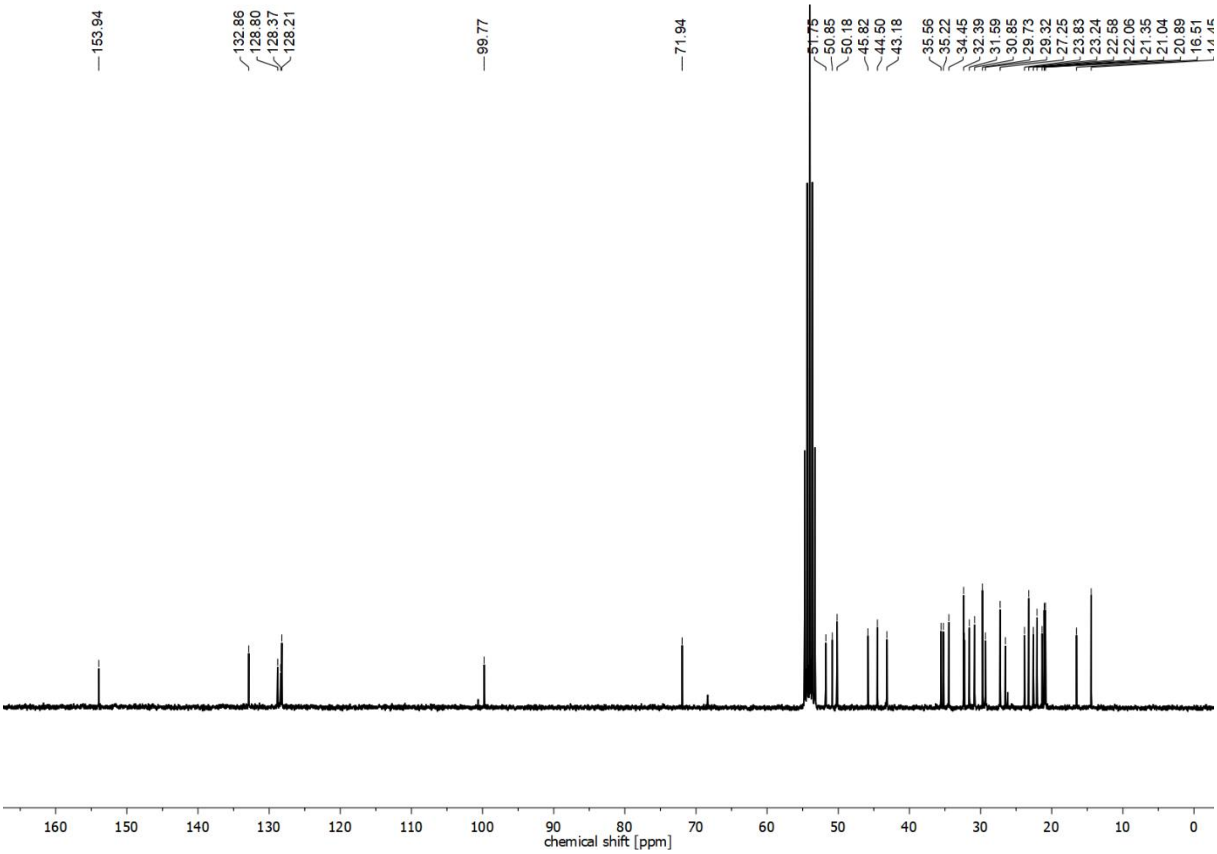

Figure S16: ^{13}C NMR spectrum of **15^{R2}** in CD_2Cl_2.

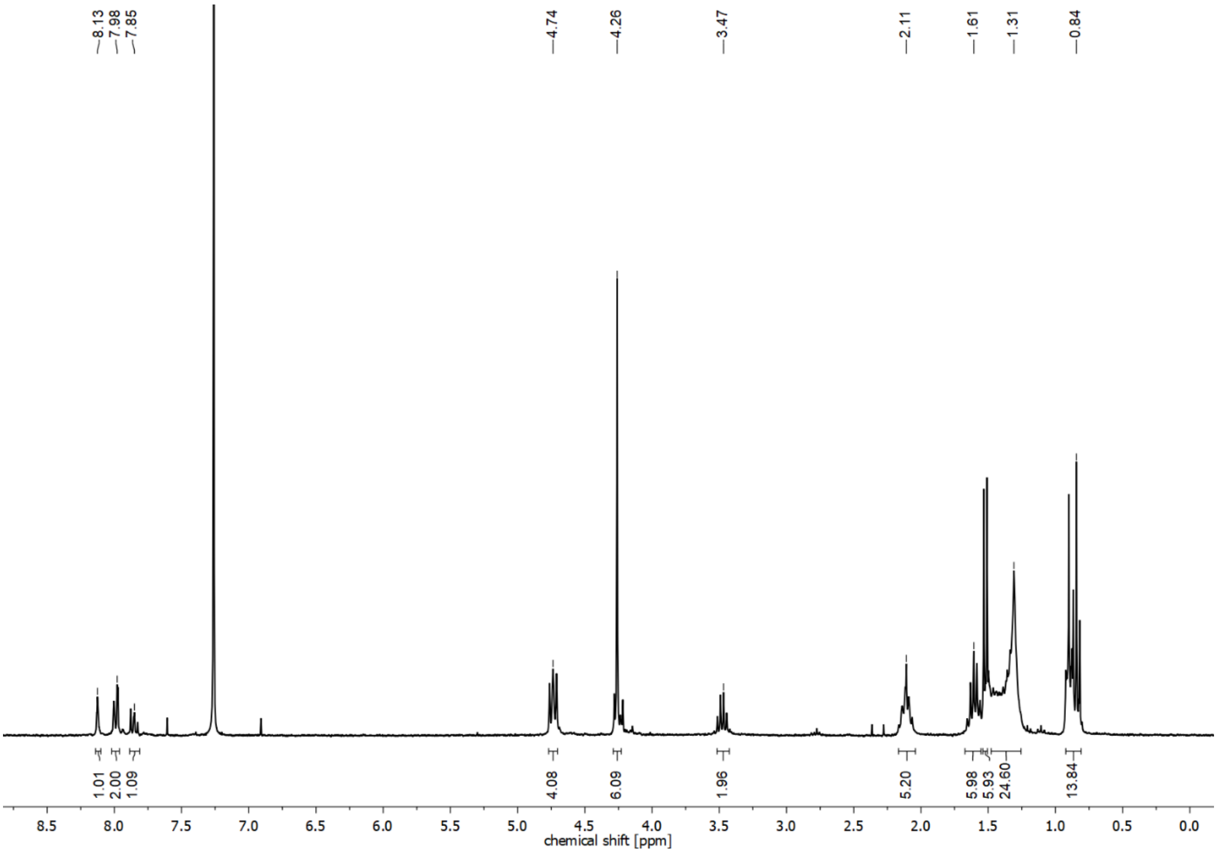

Figure S17: ^{1}H NMR spectrum of **$16^{R1\text{-}BF4}$** in $CDCl_3$.

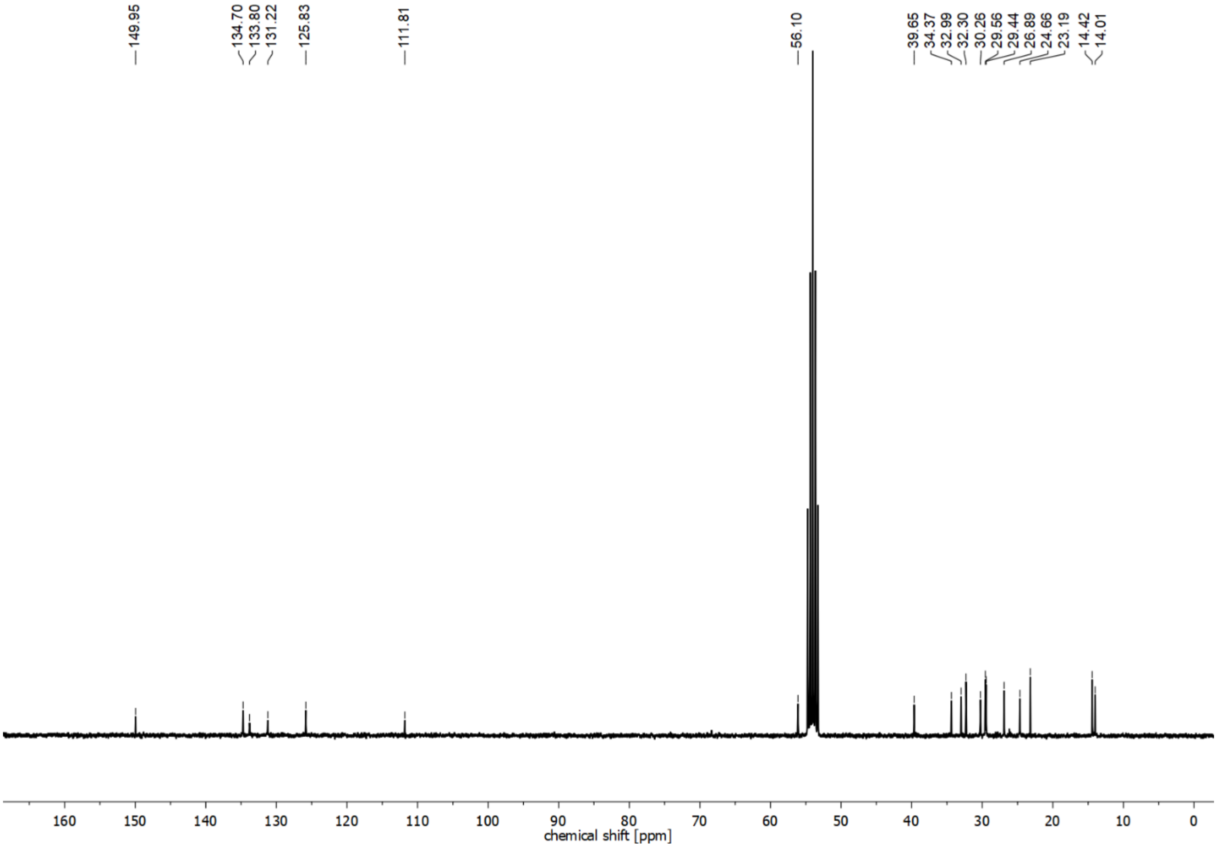

Figure S18: ^{13}C NMR spectrum of **16**$^{\mathbf{R1\text{-}BF_4}}$ in $CDCl_3$.

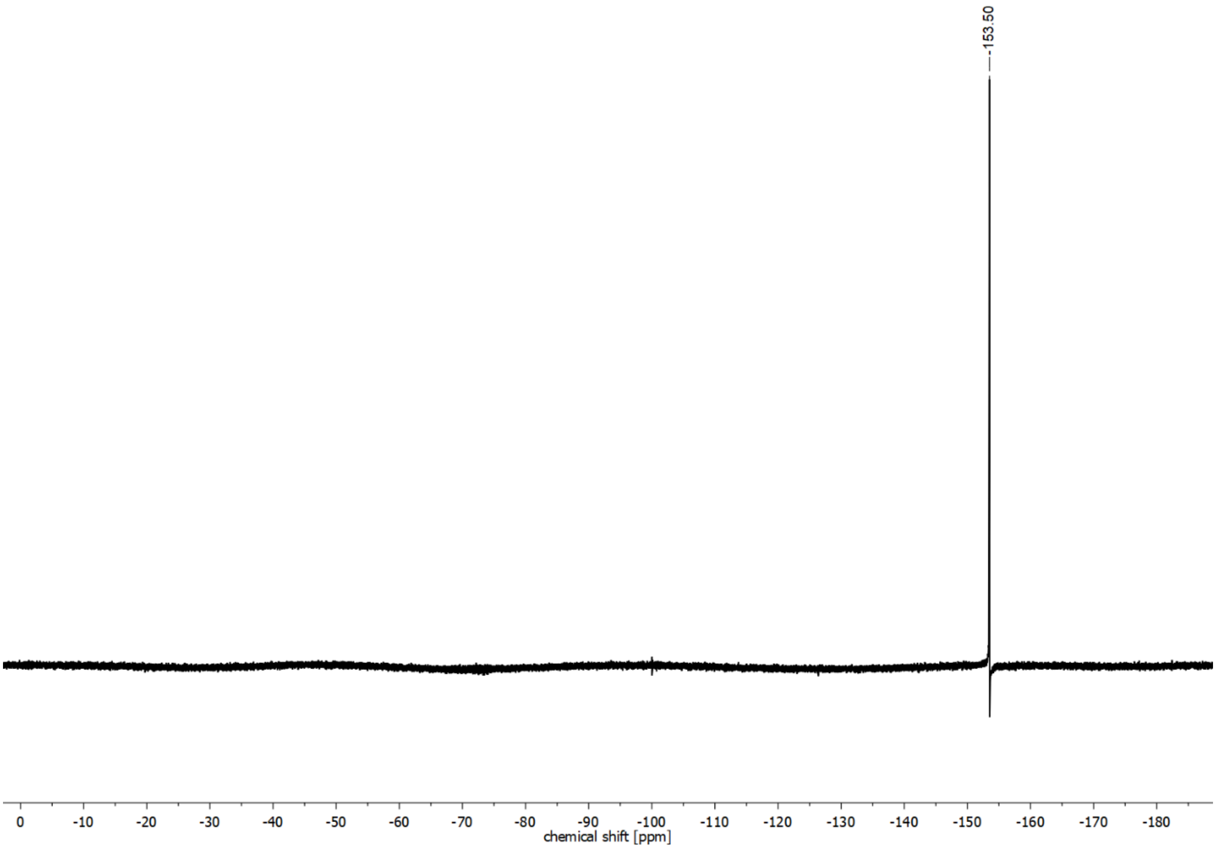

Figure S19: ^{19}F NMR spectrum of **16**$^{\mathbf{R1\text{-}BF_4}}$ in $CDCl_3$.

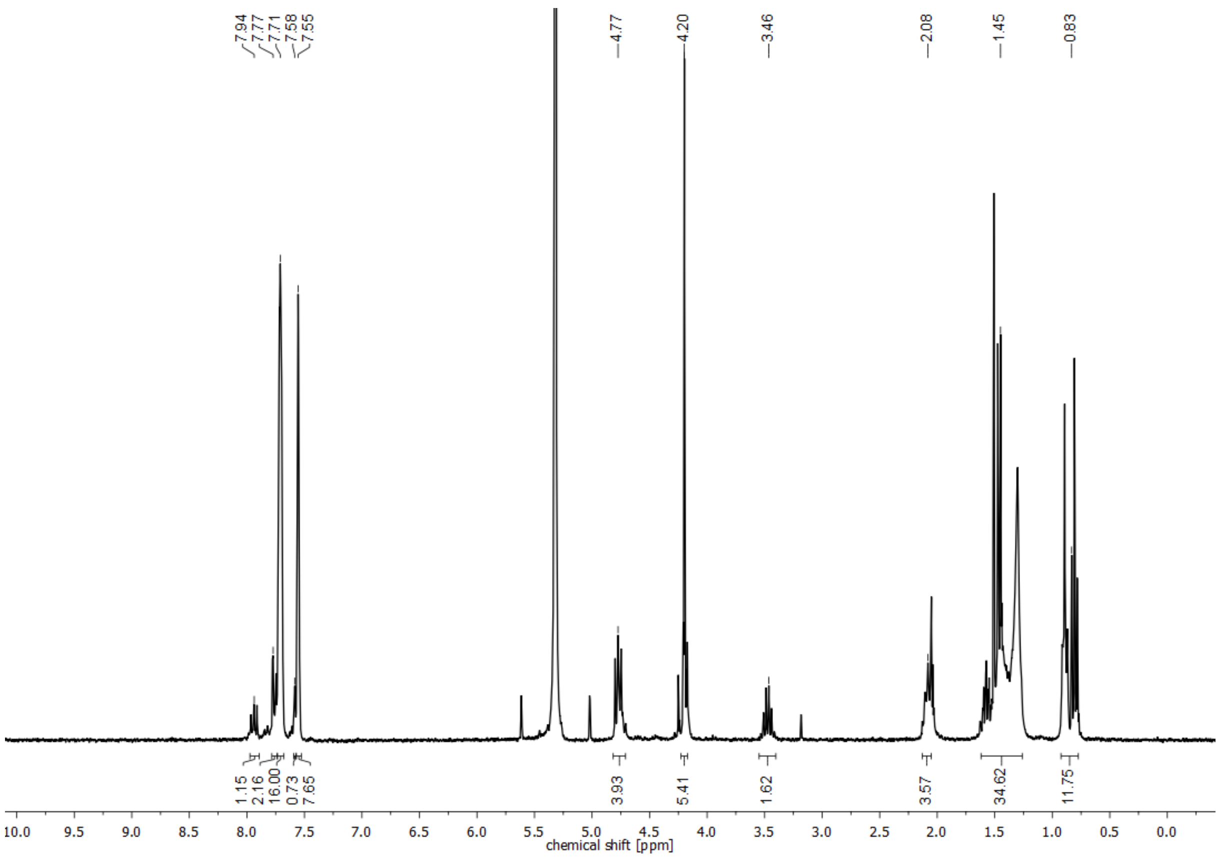

Figure S20: ^{1}H NMR spectrum of **$16^{R1\text{-}BArF_4}$** in CD_2Cl_2.

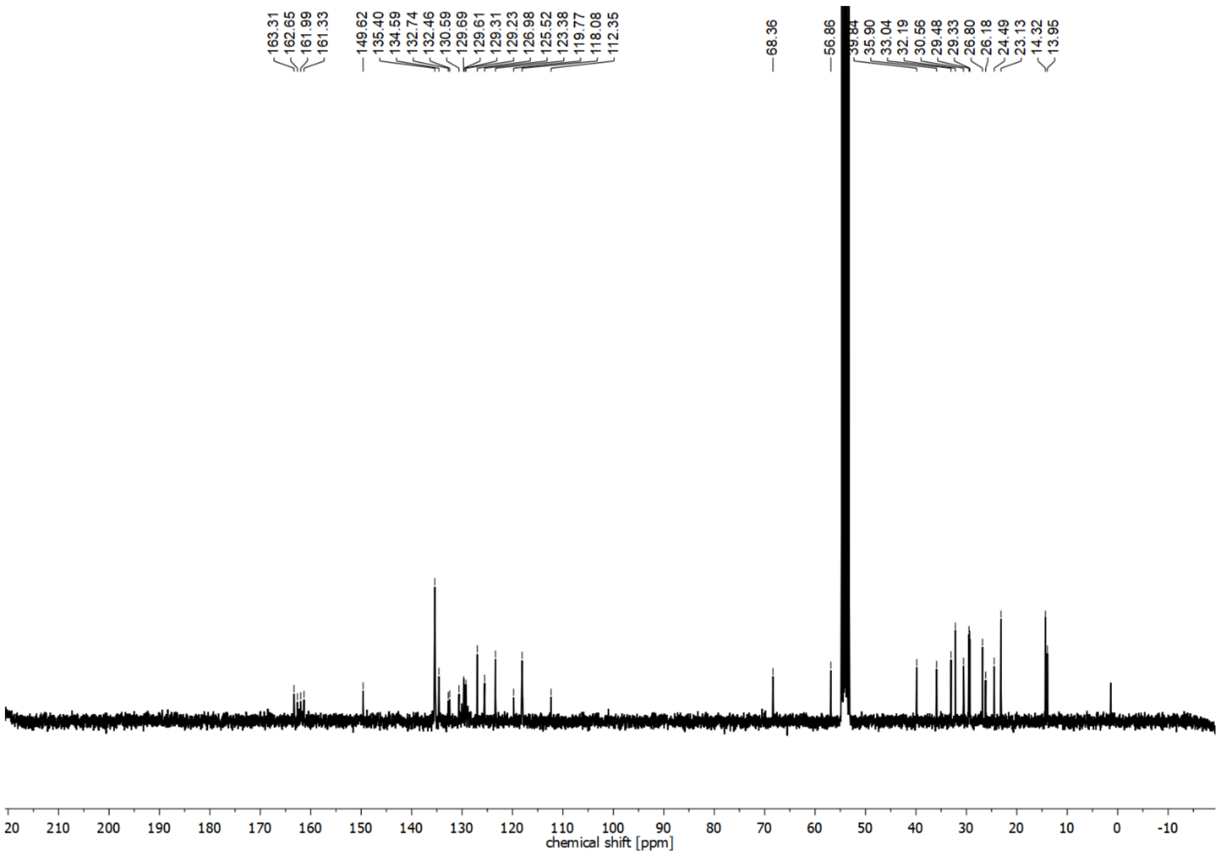

Figure S21: ^{13}C NMR spectrum of **$16^{R1\text{-}BArF_4}$** in CD_2Cl_2.

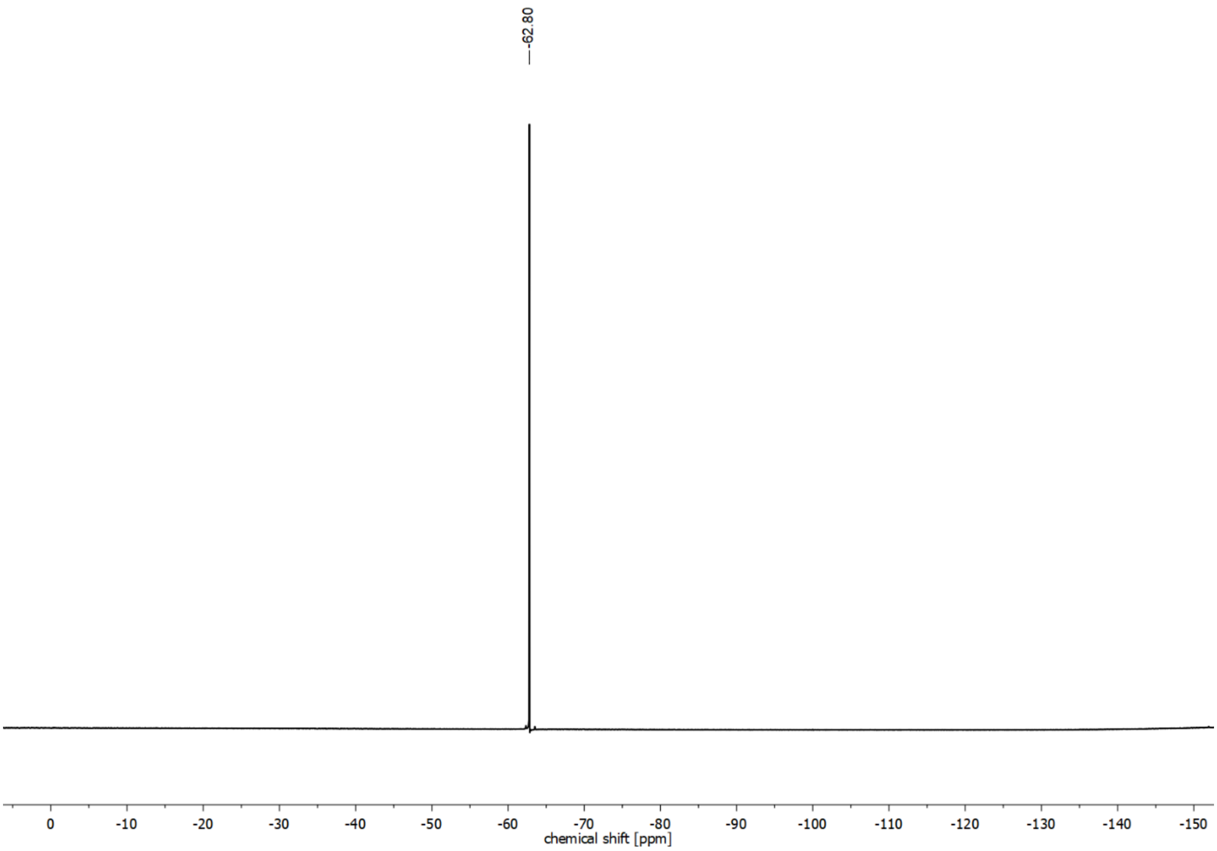

Figure S22: ^{19}F NMR spectrum of **$16^{R1\text{-}BAr^F{}_4}$** in CD_2Cl_2.

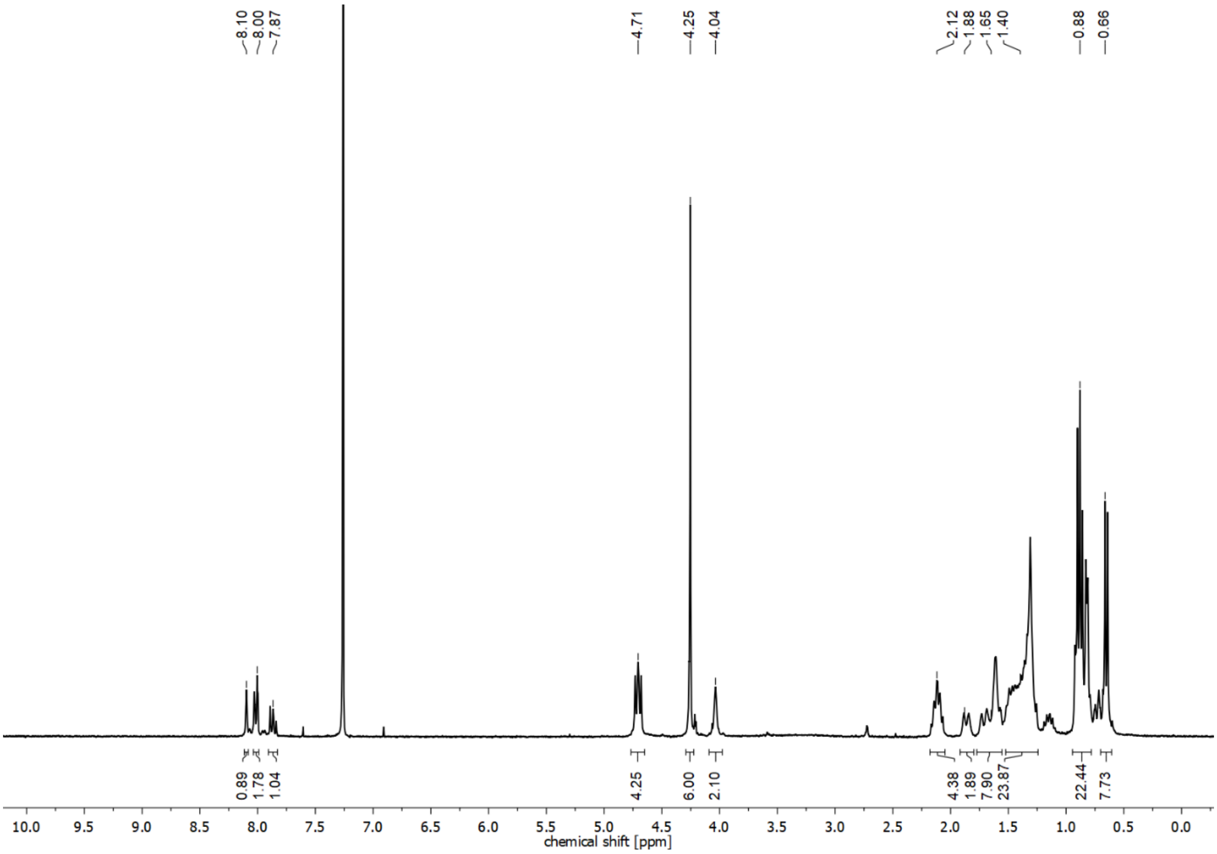

Figure S23: ^{1}H NMR spectrum of **$16^{R2\text{-}BF_4}$** in $CDCl_3$.

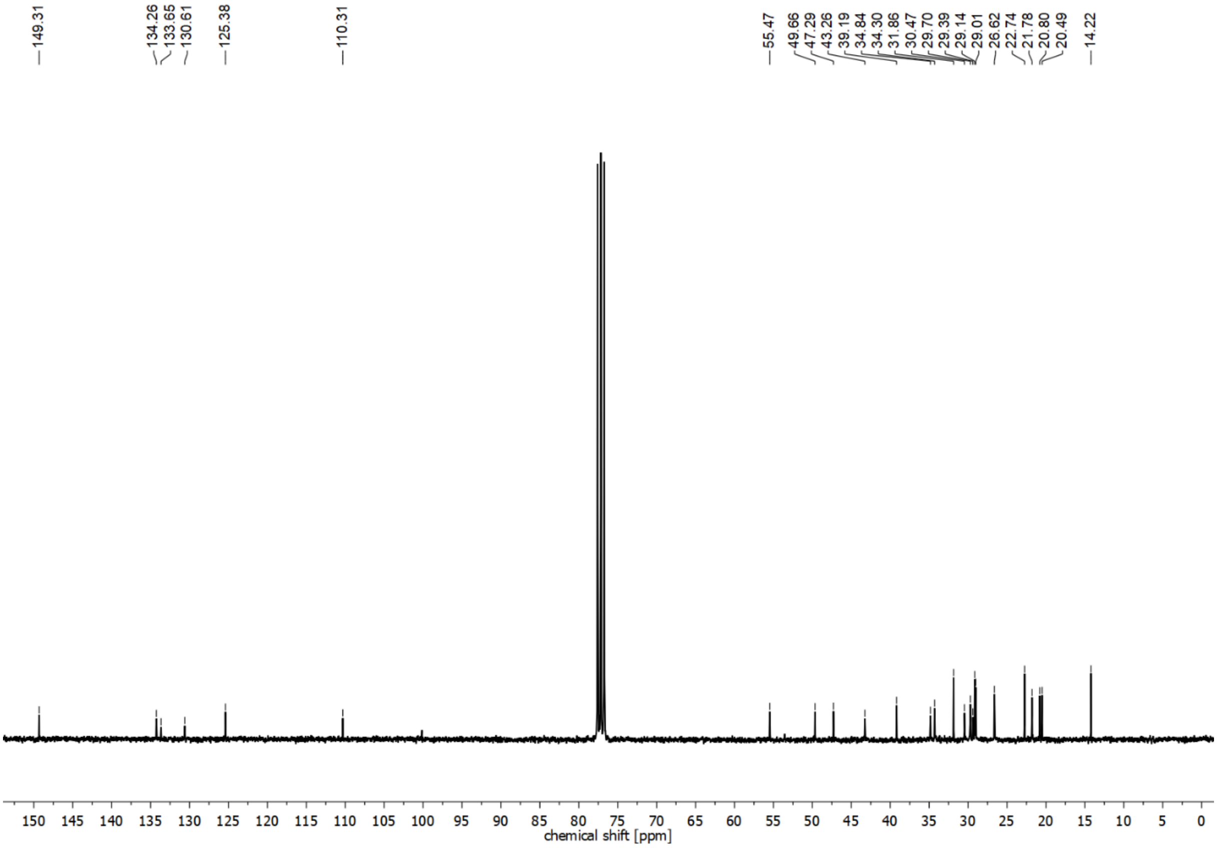

Figure S24: ^{13}C NMR spectrum of **16$^{R2\text{-}BF_4}$** in $CDCl_3$.

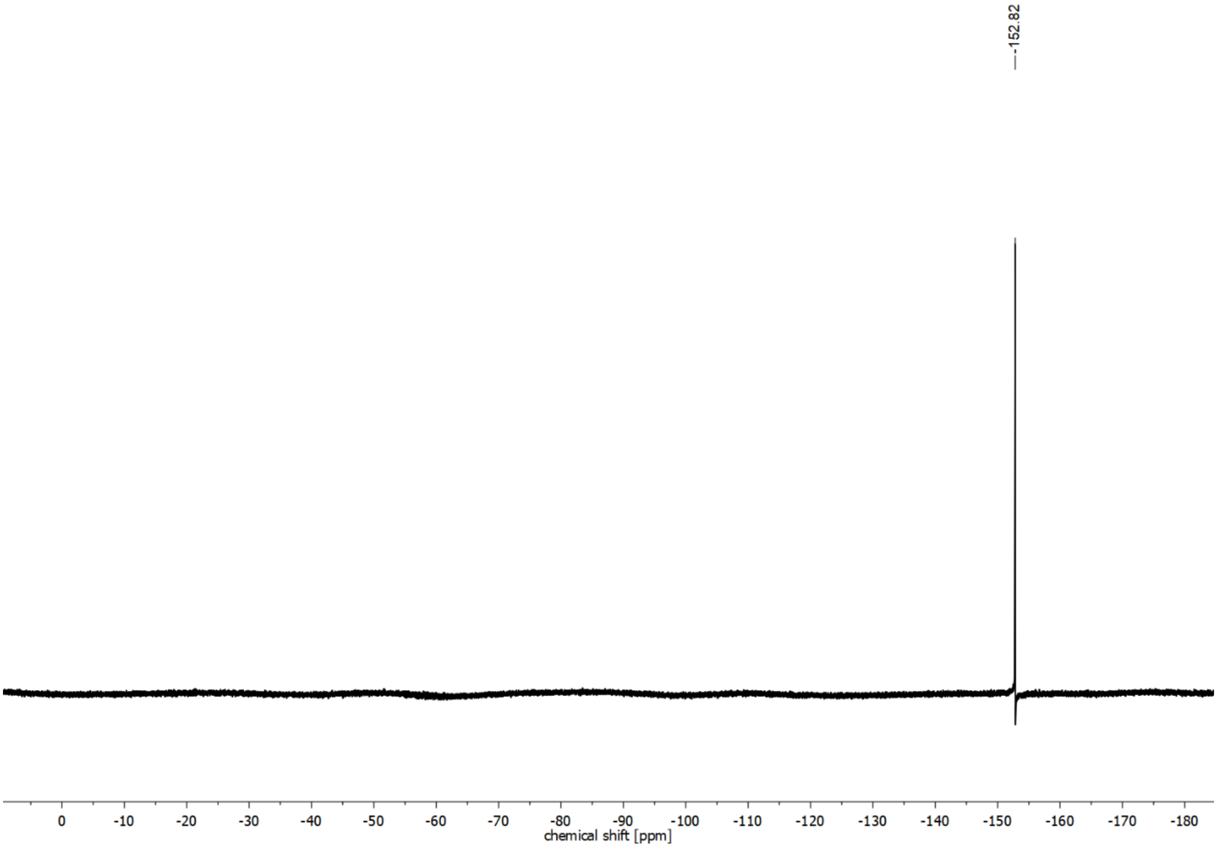

Figure S25: ^{19}F NMR spectrum of **16$^{R2\text{-}BF_4}$** in CD_2Cl_2.

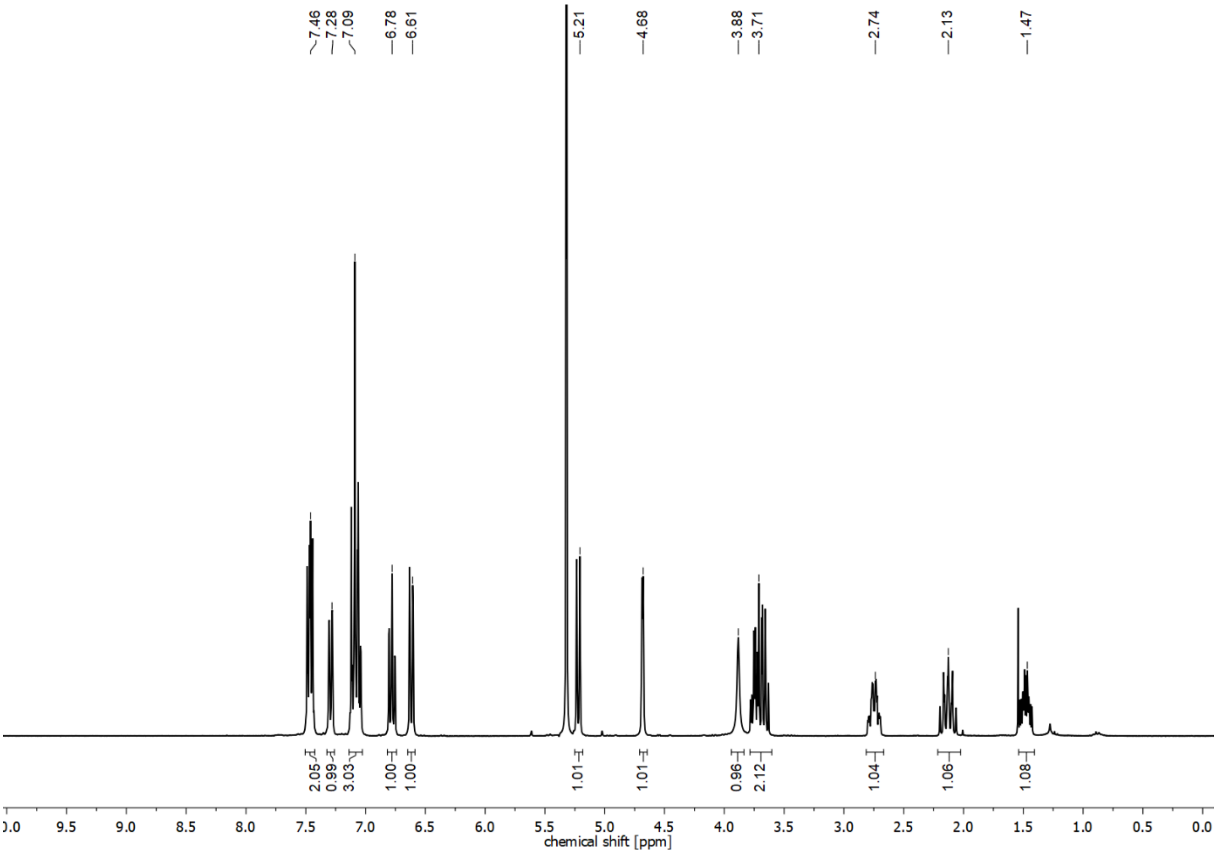

Figure S26: ^{1}H NMR spectrum of endo **(+/-)-8** in CD_2Cl_2.

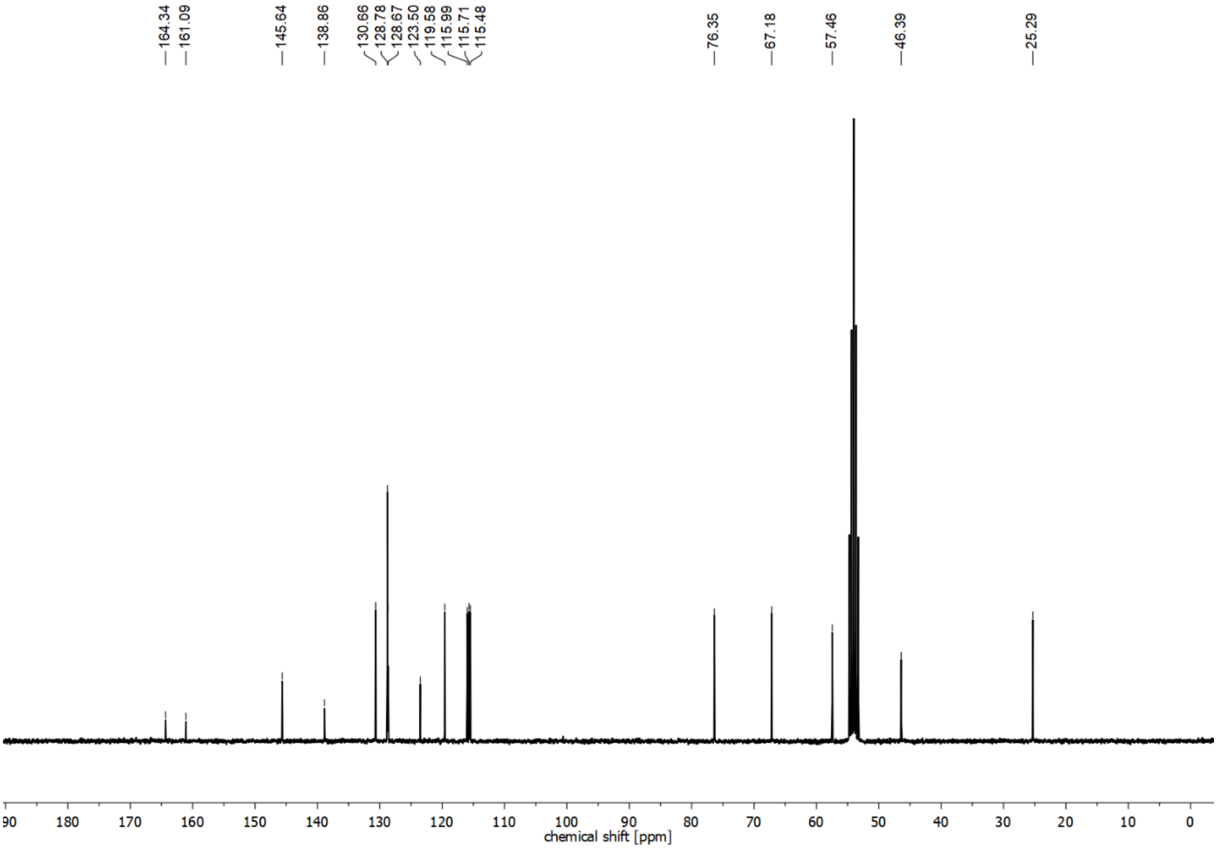

Figure S27: ^{13}C NMR spectrum of endo **(+/-)-8** in CD_2Cl_2.

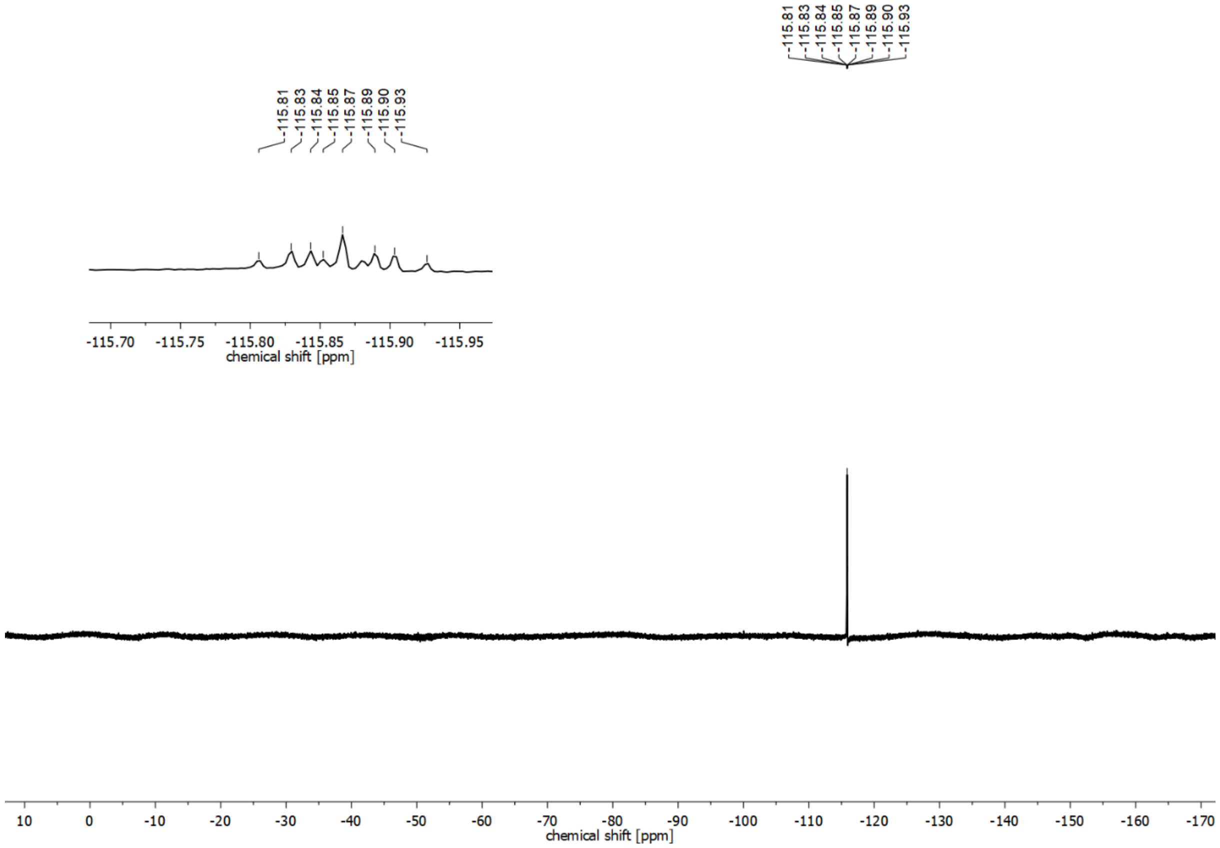

Figure S28: ^{19}F NMR spectrum of endo **(+/-)-8** in CD_2Cl_2.

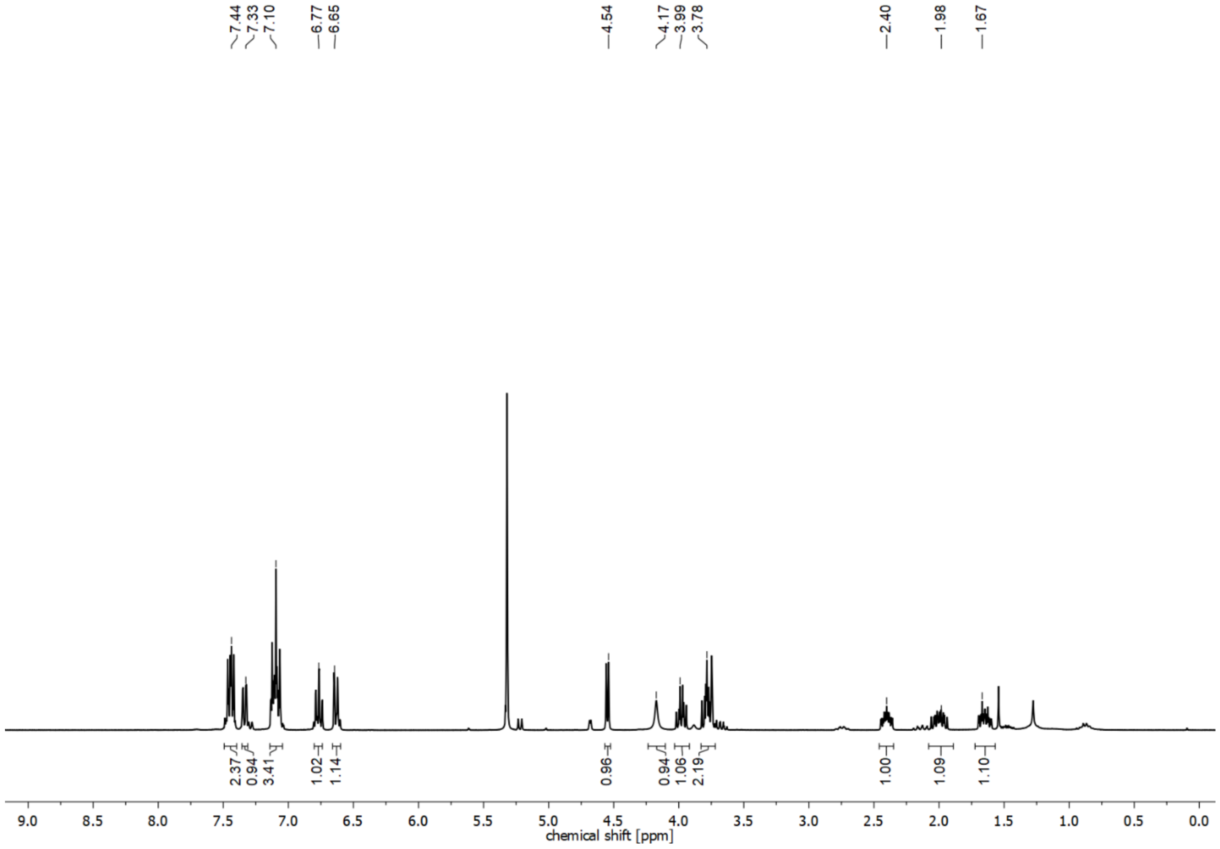

Figure S29: ^{1}H NMR spectrum of exo **(+/-)-8** in CD_2Cl_2 with small impurities of endo **(+/-)-8**.

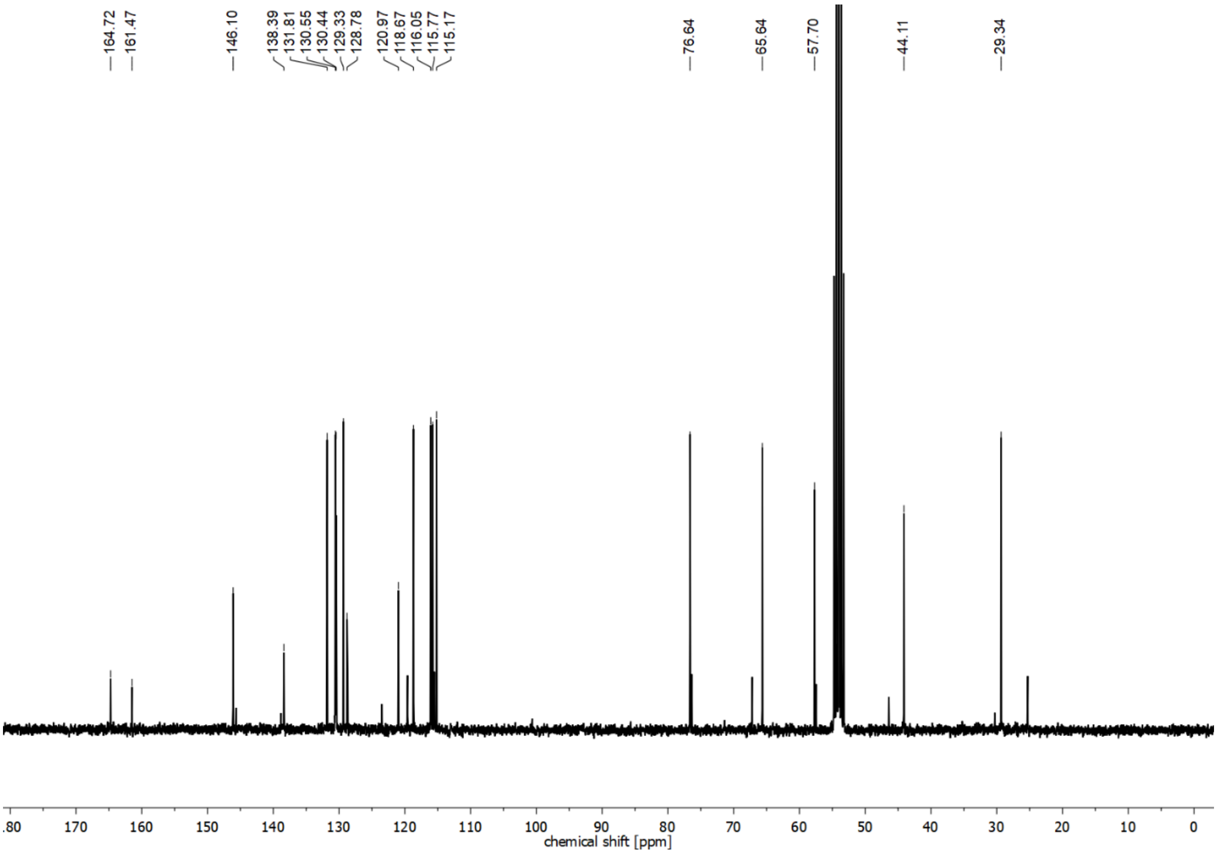

Figure 30: ^{13}C NMR spectrum of exo **(+/-)-8** in CD_2Cl_2 with small impurities of endo **(+/-)-8**.

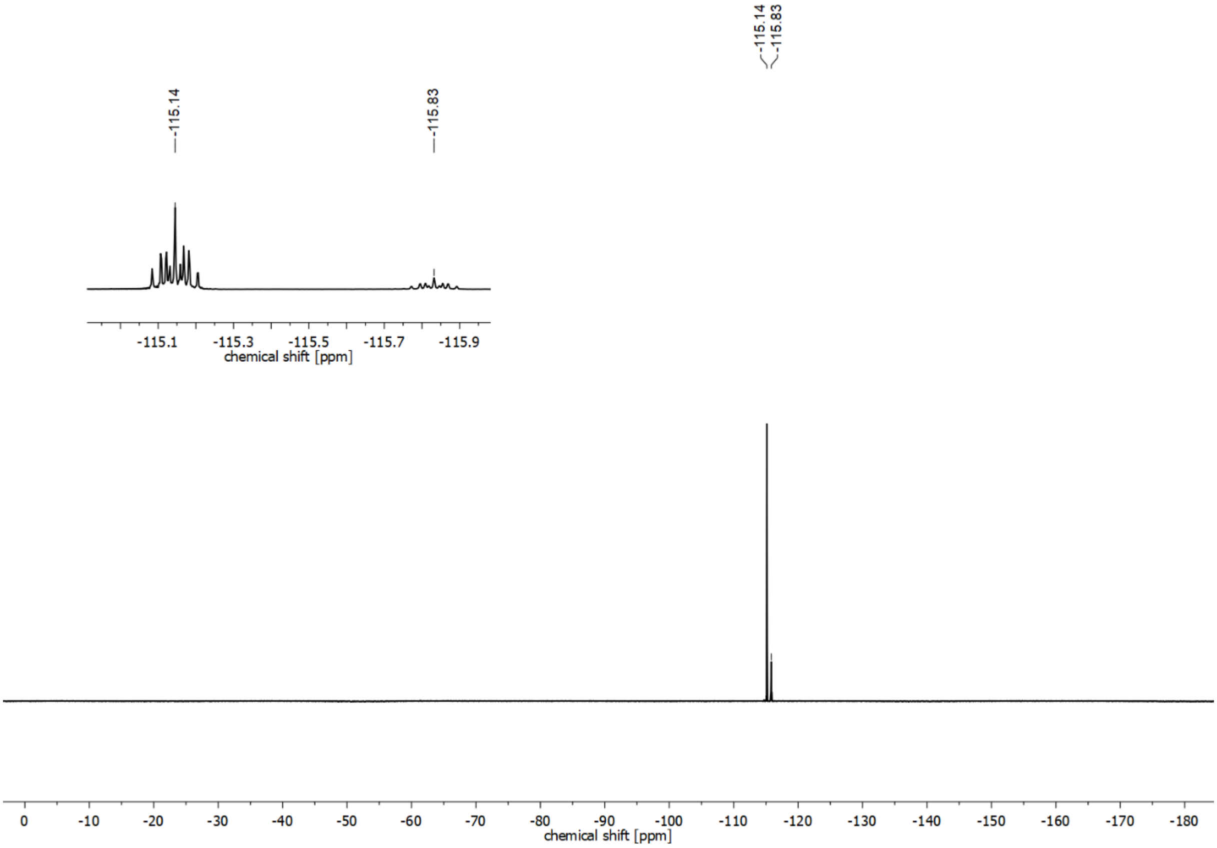

Figure S31: ^{19}F NMR spectrum of exo **(+/-)-8** in CD_2Cl_2 with small impurities of endo **(+/-)-8**.

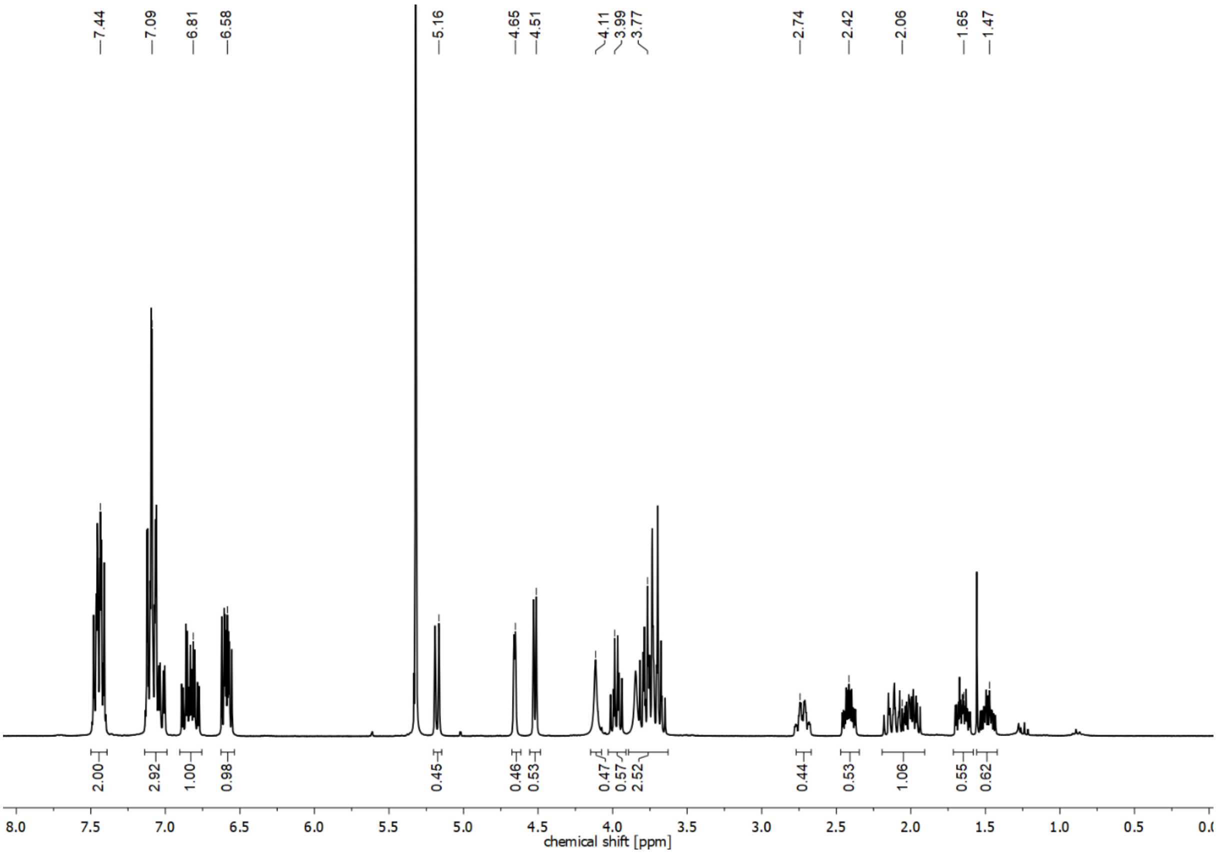

Figure S32: ^{1}H NMR spectrum of the endo/exo mixture of **(+/-)-9** in CD_2Cl_2.

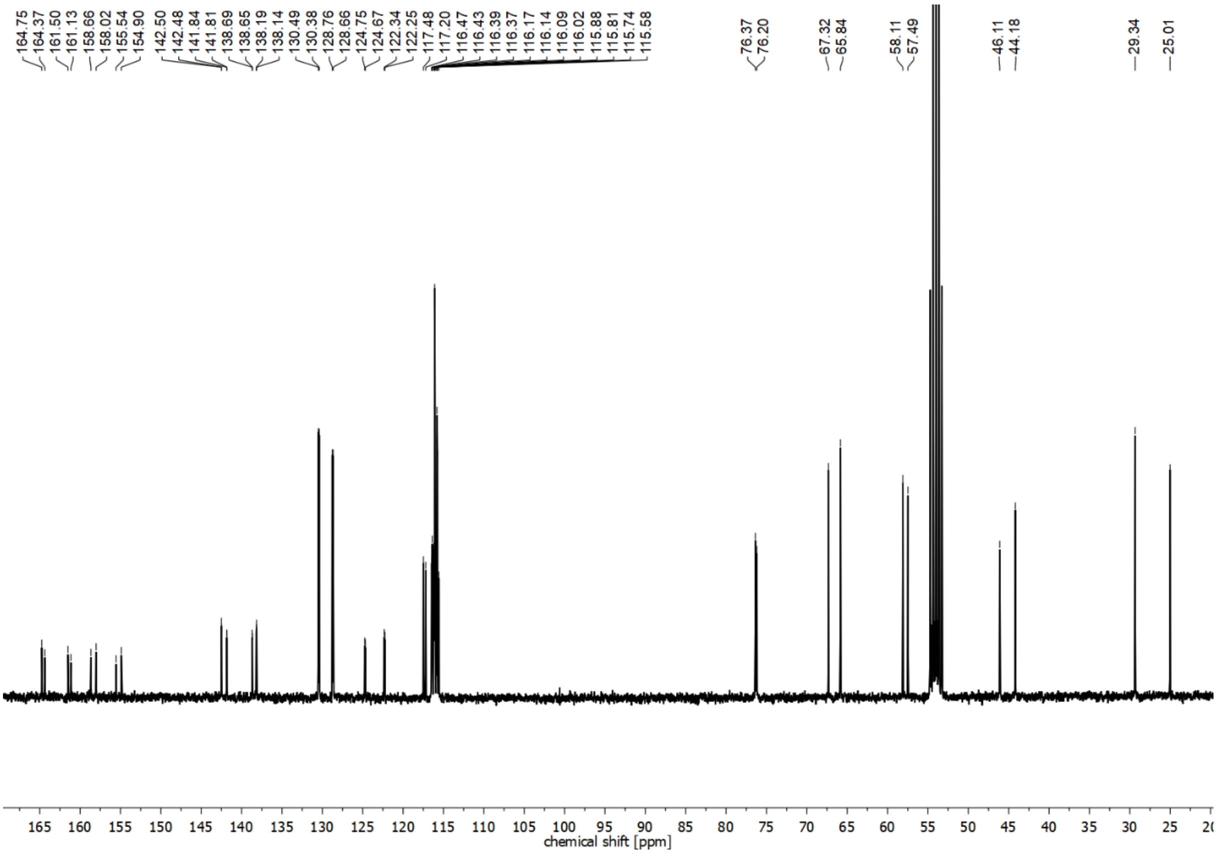

Figure S33: ^{13}C NMR spectrum of the endo/exo mixture of **(+/-)-9** in CD_2Cl_2.

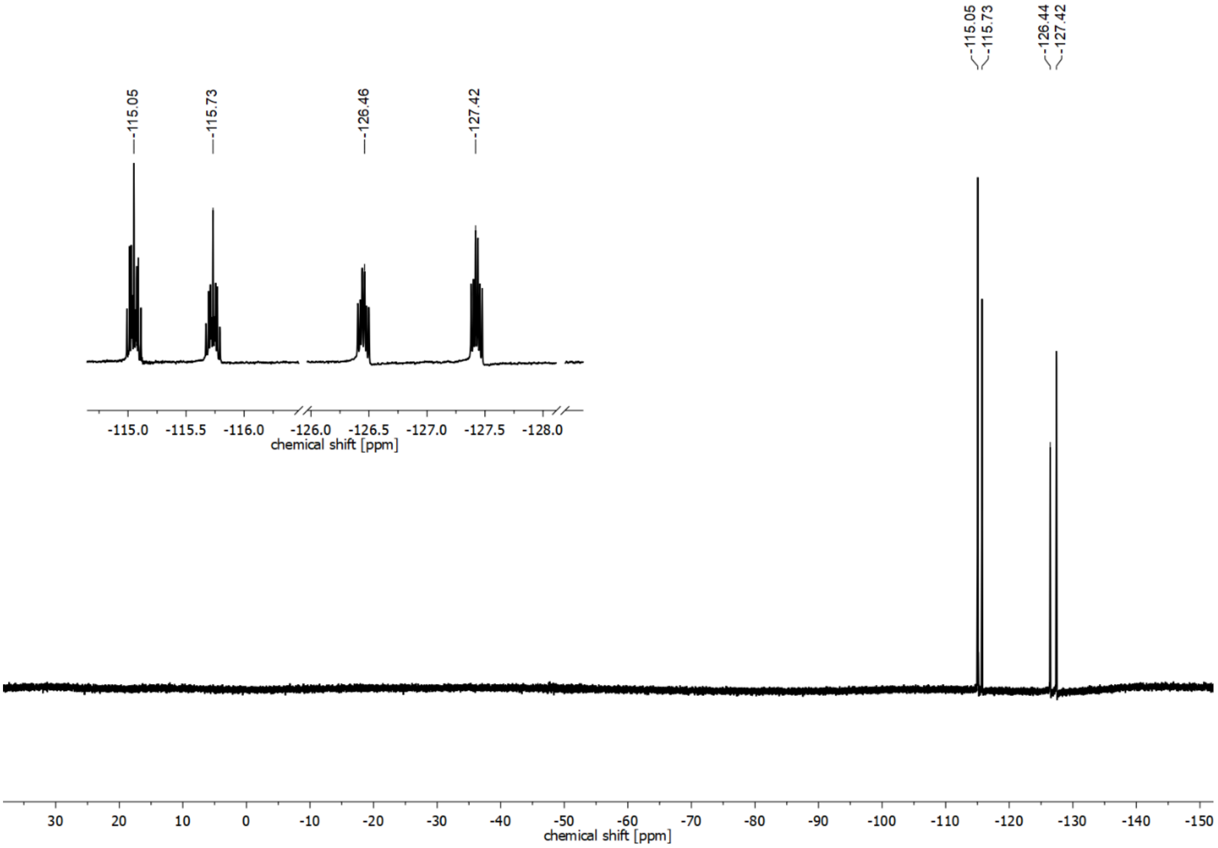

Figure S34: ^{19}F NMR spectrum of the endo/exo mixture of **(+/-)-9** in CD_2Cl_2.

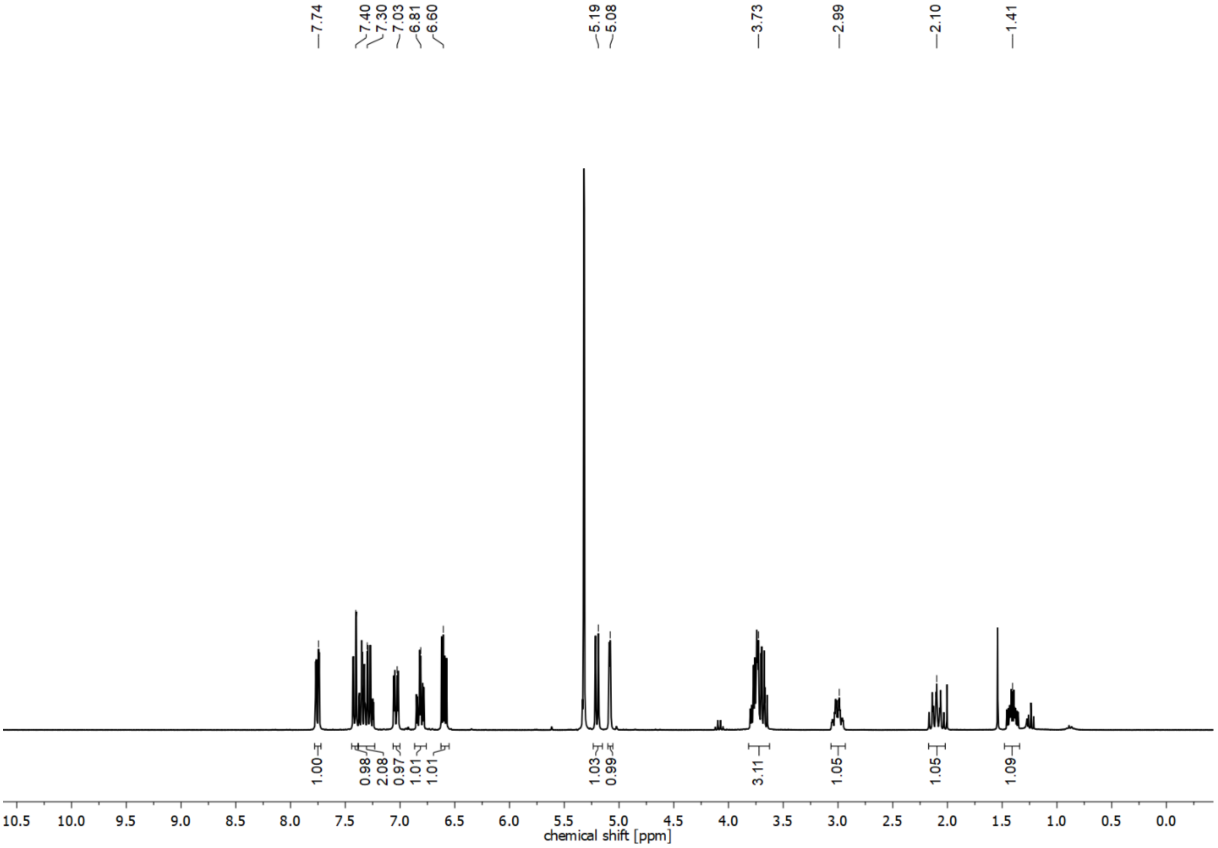

Figure S35: ^{1}H NMR spectrum of endo **(+/-)-10** in CD_2Cl_2.

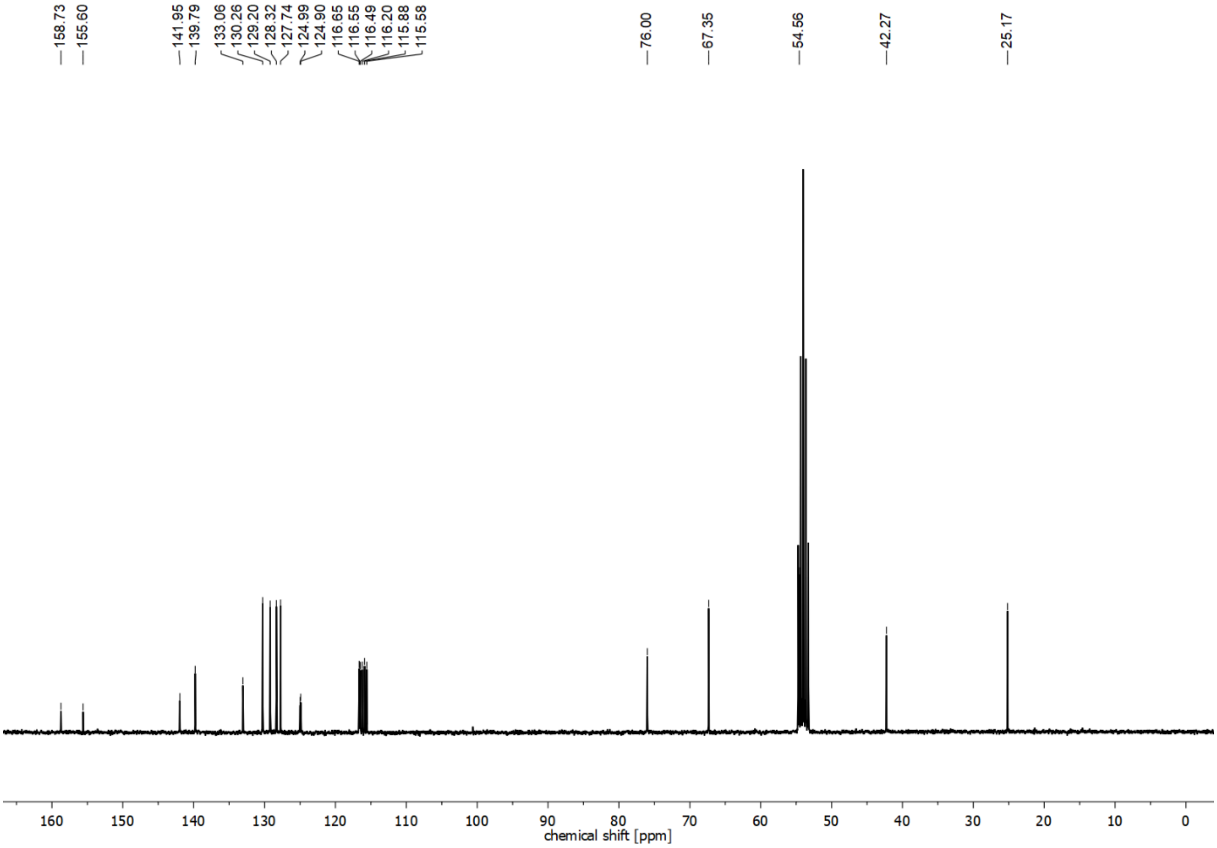

Figure S36: ^{13}C NMR spectrum of endo **(+/-)-10** in CD_2Cl_2.

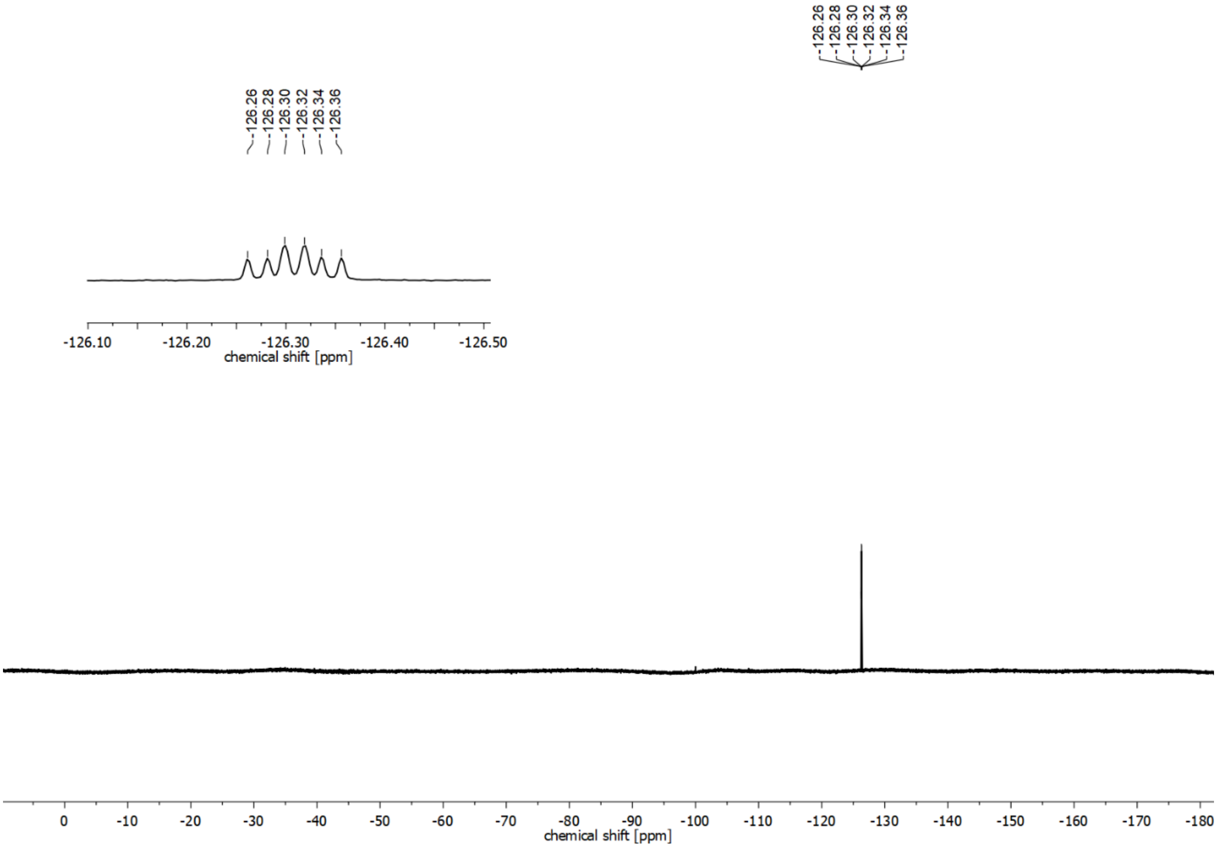

Figure 37: ^{19}F NMR spectrum of endo **(+/-)-10** in CD_2Cl_2.

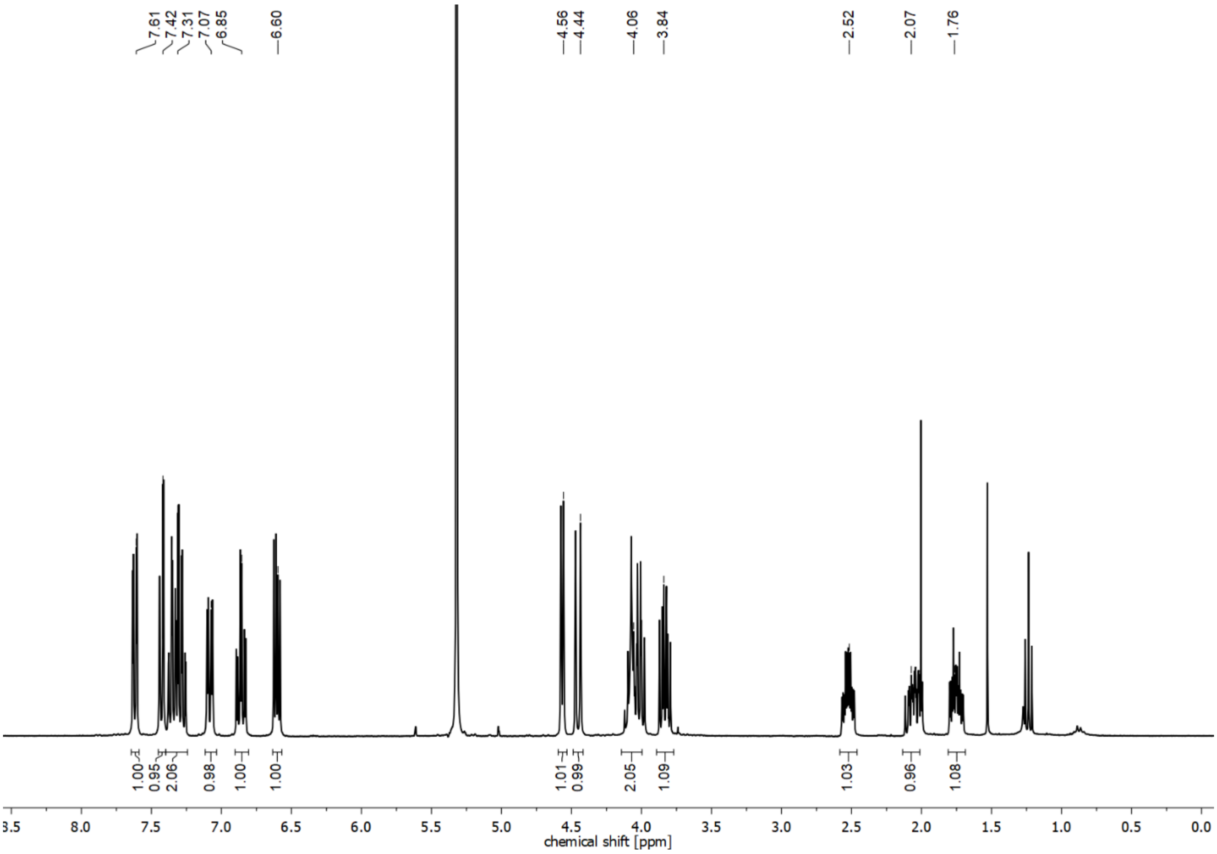

Figure S38: ^{1}H NMR spectrum of exo **(+/-)-10** in CD_2Cl_2 with residual ethyl acetate traces.

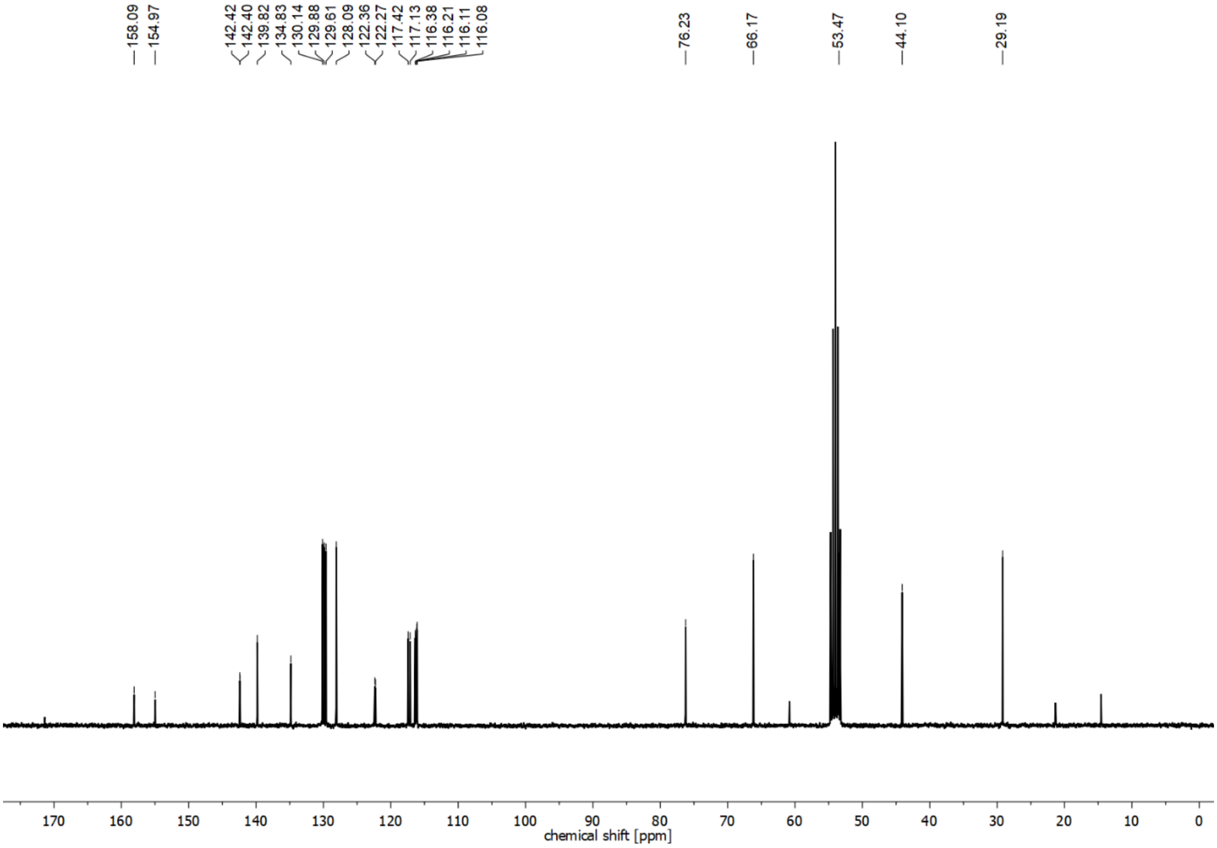

Figure S39: ^{13}C NMR spectrum of exo **(+/-)-10** in CD_2Cl_2 with residual ethyl acetate traces.

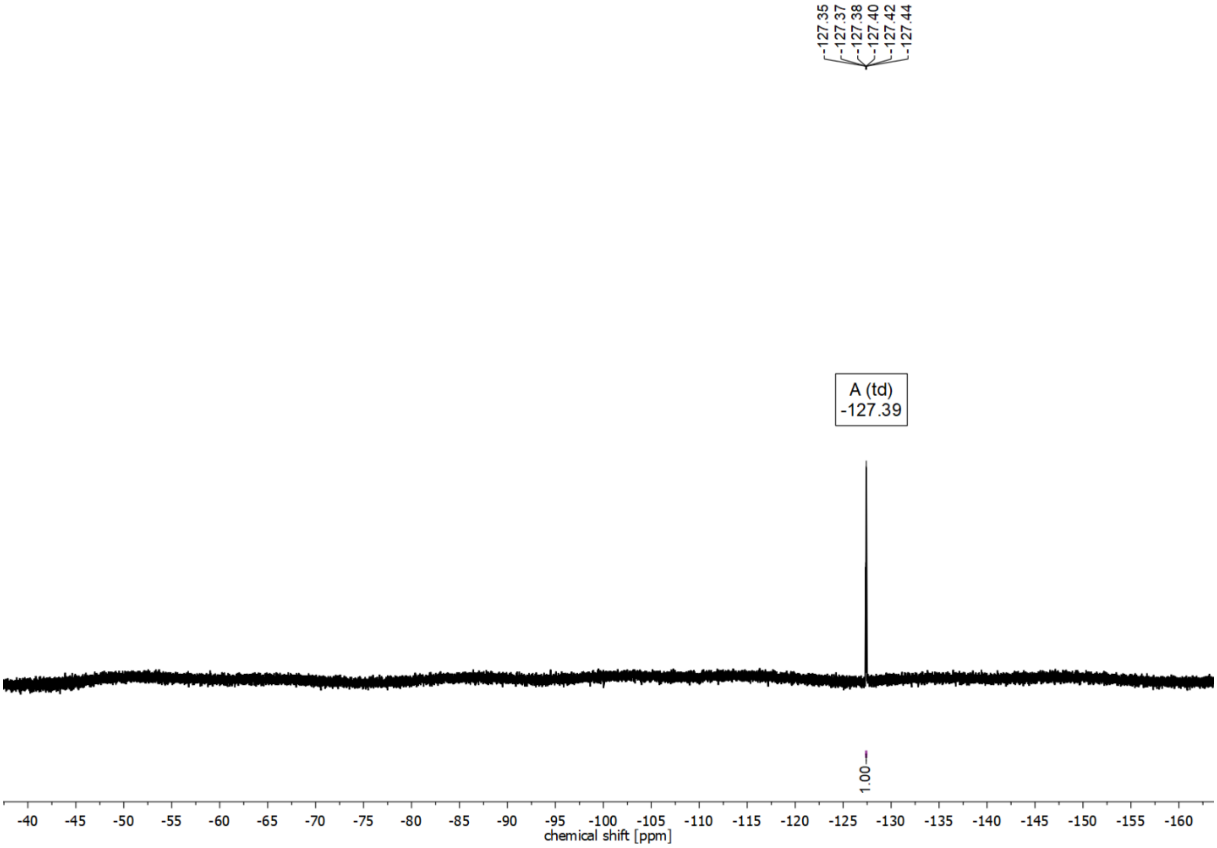

Figure S40: ^{19}F NMR spectrum of exo **(+/-)-10** in CD_2Cl_2.

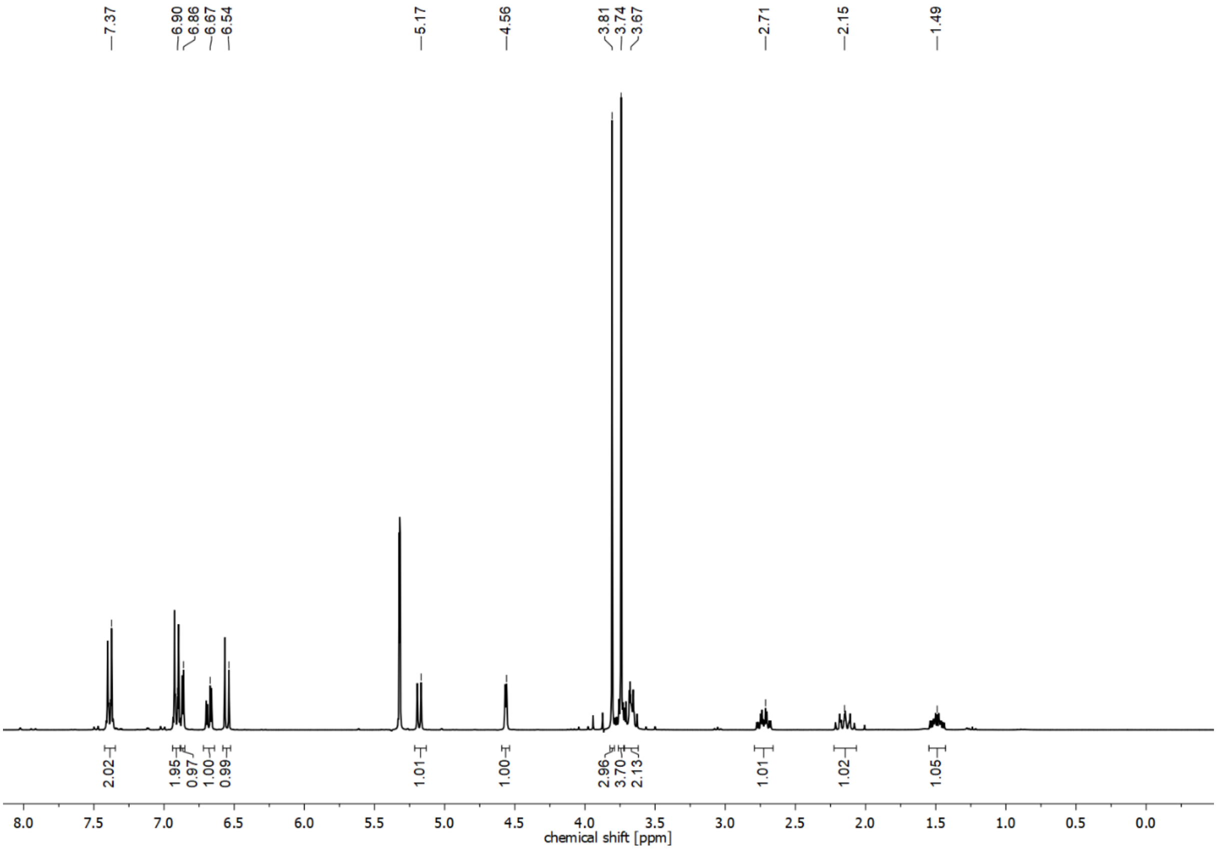

Figure S41: ^{1}H NMR spectrum of endo **(+/-)-11** in CD_2Cl_2.

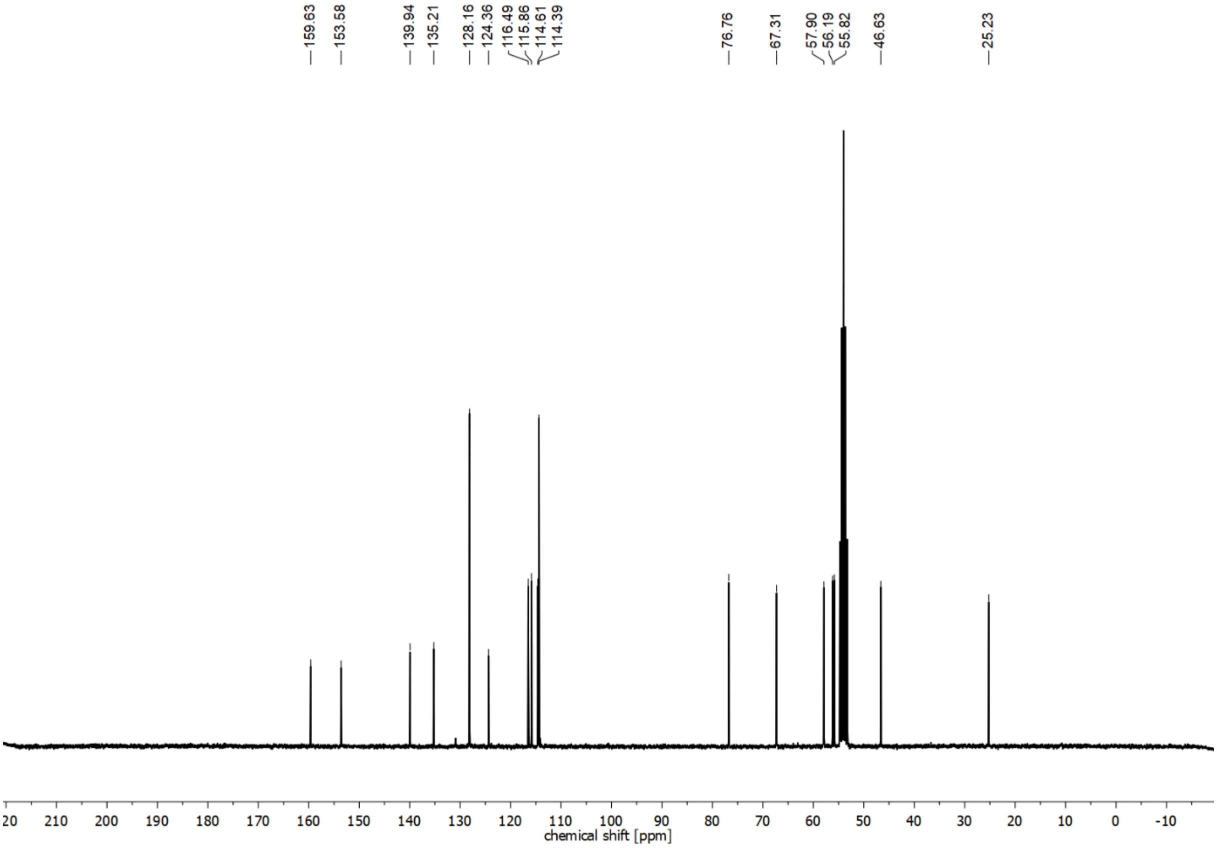

Figure S42: ^{13}C NMR spectrum of endo **(+/-)-11** in CD_2Cl_2.

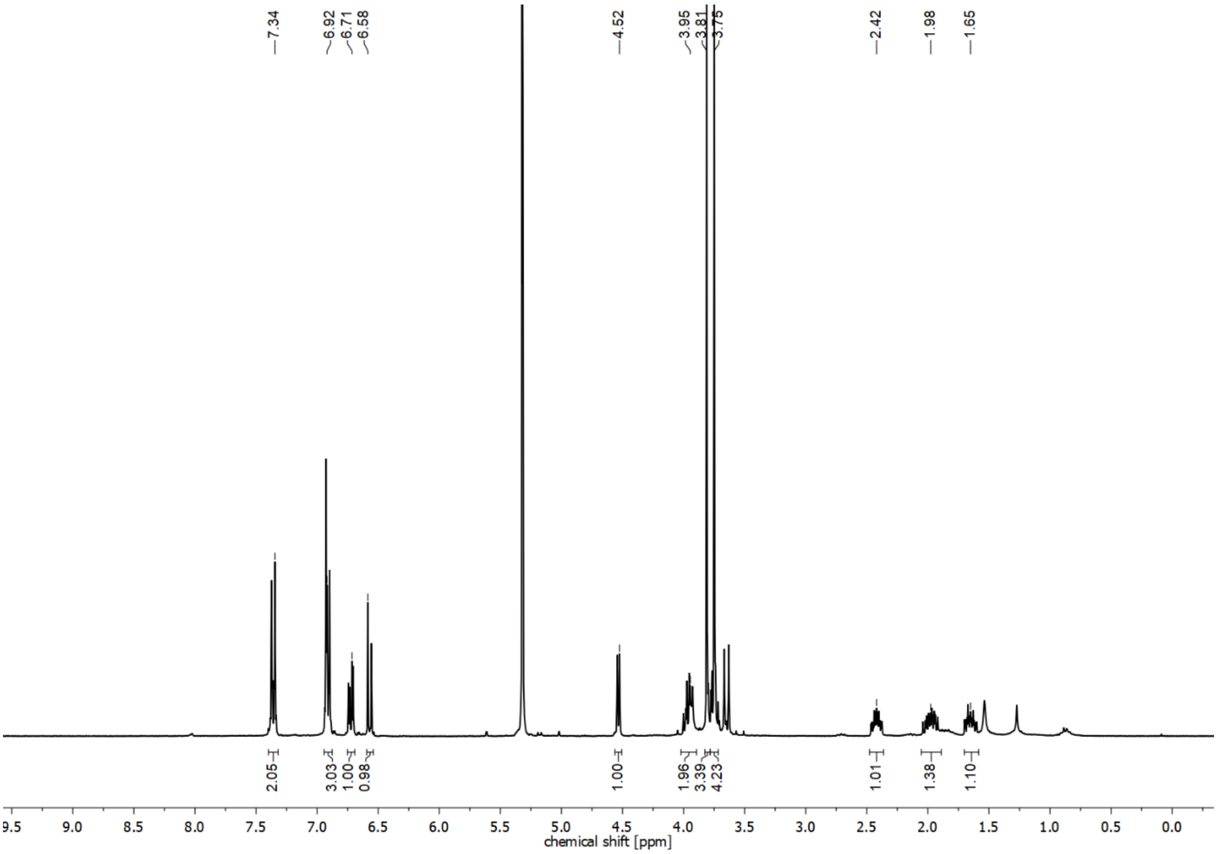

Figure S43: ^{1}H NMR spectrum of exo **(+/-)-11** in CD_2Cl_2.

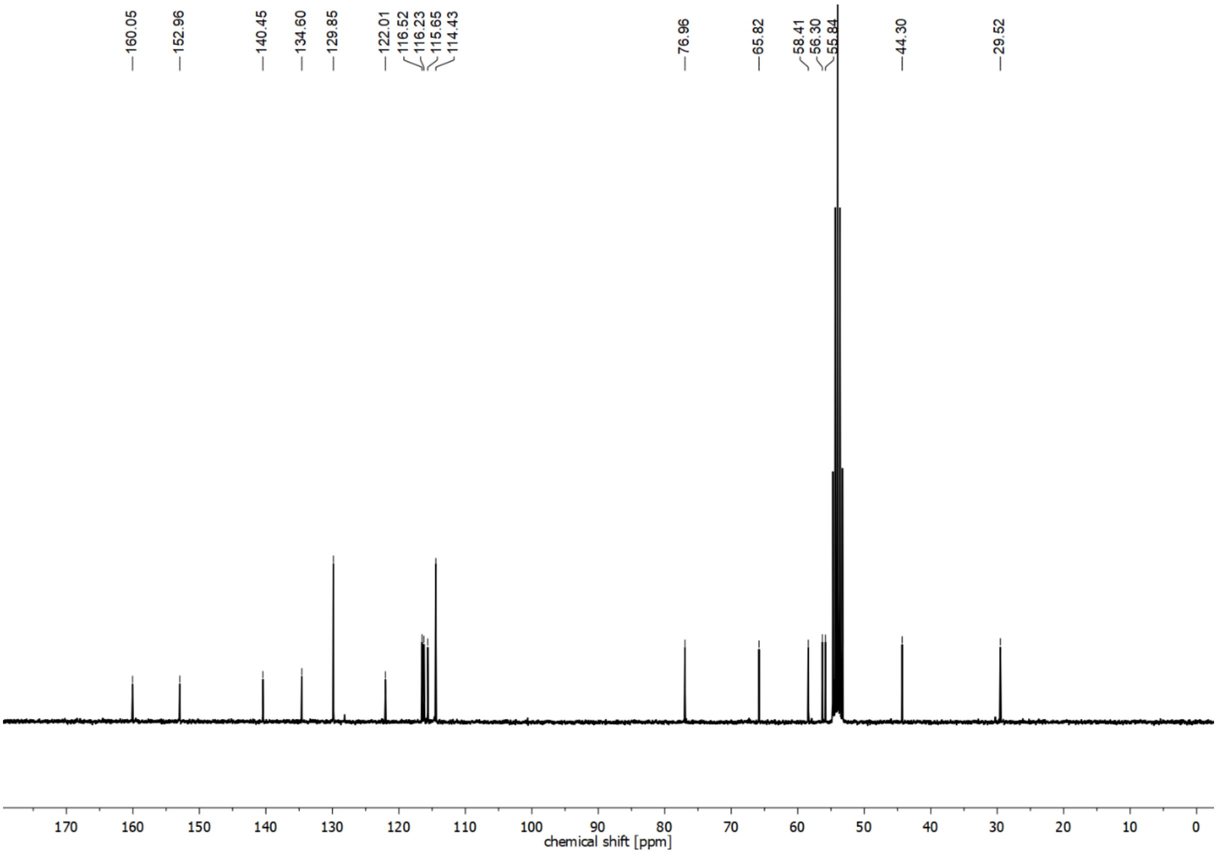

Figure S44: ^{13}C NMR spectrum of exo **(+/-)-11** in CD_2Cl_2.

6 DFT Calculations

DFT gas phase calculations were performed using the M062X[9] density functional with D3 dispersion correction[10] and the triple-zeta def2-TZVP(F) basis set[11] using Gaussian 16.[12] The nature of minima and transition states was confirmed by the appropriate number of imaginary frequencies. Imaginary frequencies of transition states were followed in both directions to ensure that they connect the aspired starting complex and product complex. For all calculations, the all-methylated (on triazolium) variant **4^{Te}** was used to save computer time. Figures were created with CYLview.[13]

Complex of 4^{Te} with 1

First, the complex of **4^{Te}** with imine **1** was calculated (see Figure S45) and the corresponding coordinates are provided below.

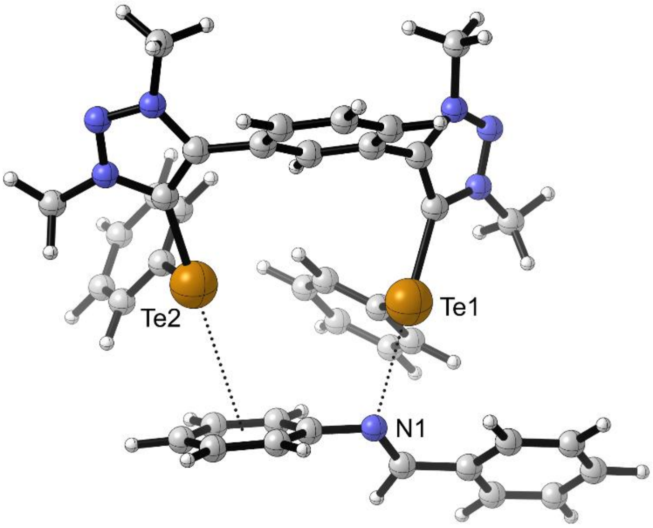

Figure S45: DFT calculation of the complex between the all methylated catalyst **4**$^{\mathbf{Te}}$ and imine **1**.

C	1.18213200	4.42412200	-1.30459800
C	0.97849200	3.46180400	-0.31819200
C	1.88624800	2.41872700	-0.16667100
C	3.00649100	2.34628900	-0.99040300
C	3.19445600	3.30488600	-1.98558400
C	2.28508800	4.33940900	-2.14045900
C	3.99180800	1.27358600	-0.78483600
C	-0.19242800	3.53421000	0.57210900
C	3.84546900	-0.09559900	-0.66183400
N	5.11768700	-0.54516300	-0.46226100
N	5.99929100	0.40926500	-0.44223000
N	5.32816600	1.50493000	-0.64404000
N	-0.38242400	4.52802300	1.48303400
N	-1.47525100	4.36186900	2.16843700
N	-2.00908200	3.27143800	1.69966600
C	-1.27498800	2.69023400	0.70828800
C	5.56730100	-1.92107300	-0.25455400
C	-3.26842700	2.79680200	2.26840100
C	0.45969700	5.69176000	1.75136500
C	6.05764600	2.77337400	-0.66705300
Te	2.14977200	-1.35824500	-0.84050700
Te	-1.88494000	1.00404000	-0.46985900
C	-1.96002400	-0.25710200	1.22714900
C	-0.78068600	-0.70968100	1.80960300
C	-0.82883600	-1.61159700	2.86514100
C	-2.05468600	-2.06459800	3.33458100
C	-3.23318000	-1.60577800	2.75813000
C	-3.18992100	-0.69858700	1.70674300
C	2.54320500	-2.23878300	1.04562800
C	2.72810700	-1.43803800	2.16952700
C	3.02197900	-2.02316200	3.39444600
C	3.10539400	-3.40573800	3.50359800
C	2.90912300	-4.20269800	2.38400100
C	2.64015700	-3.62209200	1.15018400
H	0.46571900	5.22587900	-1.43531800

H	1.72374200	1.67152700	0.60070000
H	4.04300600	3.23460400	-2.65446500
H	2.43094300	5.07427800	-2.92018800
H	6.64616900	-1.93039300	-0.37119200
H	5.28071300	-2.24298200	0.74491700
H	5.08799500	-2.55313700	-0.99876100
H	-3.67799100	3.59870800	2.87414500
H	-3.93814700	2.54274600	1.44879700
H	-3.07505000	1.91272700	2.87241400
H	1.50074800	5.38236000	1.71600700
H	0.27125300	6.45931300	1.00402800
H	0.20229300	6.06103400	2.73909100
H	5.42663000	3.54416400	-0.23407700
H	6.95968500	2.63925200	-0.07815800
H	6.31736200	3.02909400	-1.69173900
H	0.17720300	-0.38423100	1.42281800
H	0.09372700	-1.97095600	3.30450000
H	-2.09365100	-2.77441000	4.15027700
H	-4.18989100	-1.95278200	3.12673400
H	-4.11174600	-0.34977700	1.25580400
H	2.64926300	-0.35896700	2.09810100
H	3.17646700	-1.39908200	4.26513900
H	3.32795300	-3.86051600	4.45959800
H	2.98135900	-5.27938300	2.46435300
H	2.51855300	-4.24764600	0.27488400
N	-2.84360900	-1.46443200	-1.54075700
C	-3.98529100	-1.95866400	-1.27262300
H	-4.09251700	-3.03807200	-1.11944400
C	-1.74789800	-2.35653800	-1.61791300
C	-1.02422500	-2.44105800	-2.80728800
C	-1.38192100	-3.13563700	-0.52342900
C	0.01390100	-3.35147900	-2.91872100
H	-1.31753900	-1.82401300	-3.64682000
C	-0.31994900	-4.02604200	-0.63434500
H	-1.93443800	-3.04653500	0.40533300
C	0.36897400	-4.14866300	-1.83425700
H	0.54366300	-3.44769200	-3.85752500
H	-0.05664600	-4.64392900	0.21483300
H	1.17021000	-4.87002900	-1.93378100
C	-5.20737600	-1.16644900	-1.13129100
C	-5.31912500	0.12023200	-1.66140300
C	-6.28235700	-1.72479400	-0.44002200
C	-6.48383500	0.84411800	-1.47588900
H	-4.50280400	0.52658700	-2.24309000
C	-7.44295700	-0.99199700	-0.24293700
H	-6.20488100	-2.73533000	-0.05538800
C	-7.54182100	0.29299100	-0.75853900
H	-6.58128500	1.83192500	-1.90657600
H	-8.27367600	-1.42709900	0.29594600
H	-8.45351600	0.85968800	-0.62210000

Transition states

Next, the transition state of the reaction was elucidated. Two alternatives were considered, which differ by the orientation of the phenyl groups on Te: in the more favorable (by Gibbs free energy) transition state **TS**, they are directed away from the central phenyl moiety, where in the slightly less favorable transition state **invTV**, the groups are pointing roughly towards the central core (see Figures below).

Below, we provide the coordinates and energies of all relevant structures for the uncatalyzed reaction and for the two ChB-catalyzed ones, proceeding either via transition states **TS** or **invTS**.

The transition states differ in energy ΔE by 0.4 kcal/mol (in favor of TS) and in Gibbs free energy ΔG by 2.1 kcal/mol (in favor of invTS). The barriers of the three processes are: 36.4 kcal/mol (uncatalyzed), 27.1 kcal/mol (proceeding via **TS**) and 28.9 kcal/mol (proceedings via **invTS**).

<u>Transition state (uncatalyzed)</u>

ΔE = -787.880795 ht

ΔG = -787.622365 ht

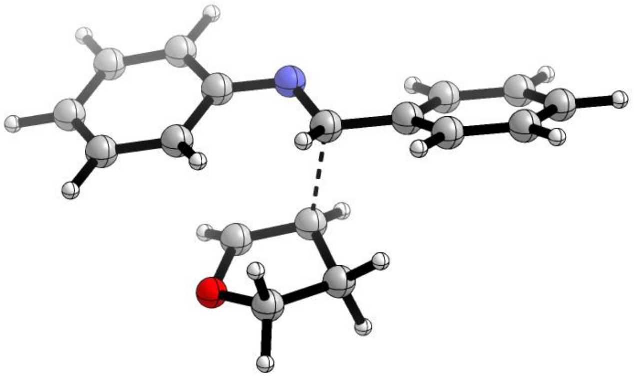

Figure S46: Transition state (for uncatalyzed reaction) according to DFT calculations.

C	-0.54421900	-0.30394400	0.46050200
H	-0.30105500	0.35494800	1.30071100
N	0.22397200	-1.36268700	0.18936600
C	1.54803700	-1.21177900	0.31581600
C	2.39565800	-2.24838600	-0.17841000
C	2.16469000	-0.00545500	0.74200500
C	3.74605700	-2.08148100	-0.24386800
H	1.91647000	-3.15861700	-0.51518000
C	3.57406100	0.12552700	0.70243100
H	1.61728200	0.69351700	1.35850700
C	4.35022100	-0.87479000	0.19313100

H	4.36919900	-2.87616000	-0.63445600
H	4.02456500	1.04143000	1.06384900
H	5.42505400	-0.76215200	0.13987900
C	-2.01129700	-0.47491000	0.28455200
C	-2.90589800	0.21463300	1.09674500
C	-2.50890200	-1.32437200	-0.70185100
C	-4.27647900	0.06609400	0.92726000
H	-2.52579200	0.85944000	1.88191900
C	-3.87489900	-1.47142600	-0.87444600
H	-1.80466900	-1.87581200	-1.31203300
C	-4.76333200	-0.77451900	-0.06208900
H	-4.96158500	0.60181700	1.57154100
H	-4.25243500	-2.13448800	-1.64233300
H	-5.83056100	-0.89268900	-0.19765200
C	0.46399000	3.06205400	0.15250400
H	0.48747100	2.83291100	1.22229600
C	-0.69710500	2.35342600	-0.54242800
H	-1.00352700	2.88976700	-1.44303900
C	1.32722500	1.28206300	-0.91220400
H	2.06631900	0.84069600	-1.56183700
C	-0.05535300	1.02559900	-0.91270200
H	-0.46852100	0.43955200	-1.72063400
O	1.64488400	2.51735900	-0.45511200
H	0.49004700	4.14071100	0.02020500
H	-1.56986600	2.25506000	0.10190900

Starting complex (uncatalyzed)

ΔE = -787.934518 ht

ΔG = -787.680297 ht

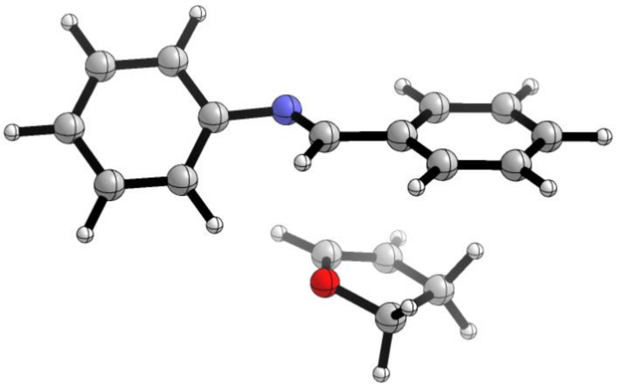

Figure S47: Starting complex (for uncatalyzed reaction) according to DFT calculations.

C	-0.14721500	-0.85637100	-0.57137100
H	-0.44487300	-0.55256000	-1.58142400
N	-0.99125400	-0.97135300	0.36592300
C	-2.33036600	-0.61618600	0.12458300
C	-3.33507000	-1.40246600	0.68765600

C	-2.67650200	0.52270300	-0.60541000
C	-4.66622900	-1.08142400	0.48431700
H	-3.04843900	-2.26612800	1.27331100
C	-4.01353400	0.84697100	-0.78854400
H	-1.89363400	1.16236800	-0.99621600
C	-5.01158800	0.04451000	-0.25458900
H	-5.43973200	-1.70626900	0.91208200
H	-4.27458200	1.73701000	-1.34682400
H	-6.05253000	0.30105500	-0.40056400
C	1.28581100	-1.11121600	-0.37229600
C	2.15471200	-1.00481700	-1.45483000
C	1.79248200	-1.44229100	0.88530800
C	3.51535200	-1.22535600	-1.29046100
H	1.75756200	-0.74800600	-2.43068400
C	3.14798000	-1.66559800	1.04858300
H	1.10369900	-1.51347300	1.71667800
C	4.01228700	-1.55619000	-0.03777600
H	4.18479800	-1.14188600	-2.13660500
H	3.53909300	-1.92211800	2.02465600
H	5.07247400	-1.72948900	0.09505100
C	1.74164600	2.38894600	-0.71138800
H	2.05290300	1.84301200	-1.59951900
C	2.54785800	2.03606100	0.54770700
H	3.27058100	2.81543300	0.78918400
C	0.27974800	1.89813300	0.92721500
H	-0.72393500	1.76237900	1.30258100
C	1.43967200	1.90933300	1.56539600
H	1.57072000	1.78343900	2.62717200
O	0.37253100	2.03879500	-0.42977900
H	1.76933300	3.46194300	-0.91480200
H	3.09282100	1.09394700	0.43810100

<u>Product complex (uncatalyzed)</u>

ΔE = -787.939507 ht

ΔG = -787.676424 ht

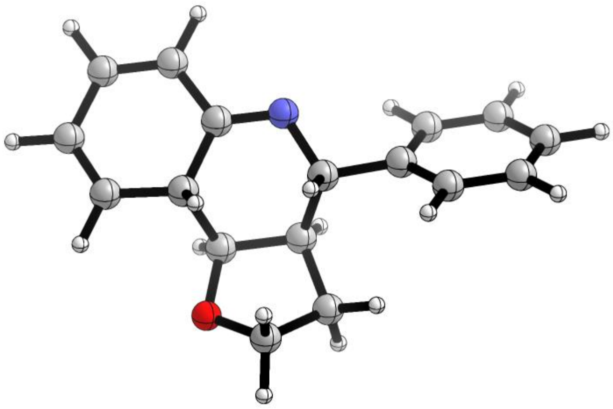

Figure S48: Product complex (for uncatalyzed reaction) according to DFT calculations.

C	-0.55328800	-0.09643400	0.33307000
H	-0.36431700	0.34090900	1.32564800
N	0.20552700	-1.32966400	0.20938300
C	1.47462600	-1.19218300	0.22772700
C	2.33678600	-2.36040700	0.09960800
C	2.10794200	0.17196000	0.35622800
C	3.66847500	-2.22704900	0.04970200
H	1.84558100	-3.32255200	0.03718300
C	3.59733800	0.19508800	0.26676100
H	1.81336800	0.58333000	1.33355300
C	4.31380600	-0.91940200	0.11970800
H	4.29563400	-3.10396000	-0.05329300
H	4.07064800	1.16858300	0.31603500
H	5.39290300	-0.87254000	0.04996000
C	-2.03368800	-0.36287800	0.21543700
C	-2.94393800	0.25618300	1.06366300
C	-2.51061000	-1.21584300	-0.77570900
C	-4.30901500	0.04128600	0.92003800
H	-2.58168500	0.90504400	1.85392400
C	-3.87160400	-1.43333100	-0.92228800
H	-1.80019100	-1.72474500	-1.41573200
C	-4.77586900	-0.80221200	-0.07639600
H	-5.00507300	0.52701500	1.59178700
H	-4.22983600	-2.10208200	-1.69452100
H	-5.83823600	-0.97522900	-0.18913200
C	0.55012600	2.99654900	0.26324200
H	0.52679000	2.73347700	1.32888600
C	-0.60439300	2.34957100	-0.48800400
H	-0.75586500	2.86318500	-1.43728700
C	1.45448600	1.11203800	-0.68532900
H	1.91245700	0.92279900	-1.65711300
C	-0.07964200	0.92758500	-0.72773000
H	-0.39164400	0.54326200	-1.69845100
O	1.71204100	2.46658600	-0.35419500
H	0.58956000	4.08079100	0.17593600
H	-1.54434400	2.36455100	0.06212900

Transition state (TS)

ΔE = -2658.113179 ht

ΔG = -2657.417148 ht

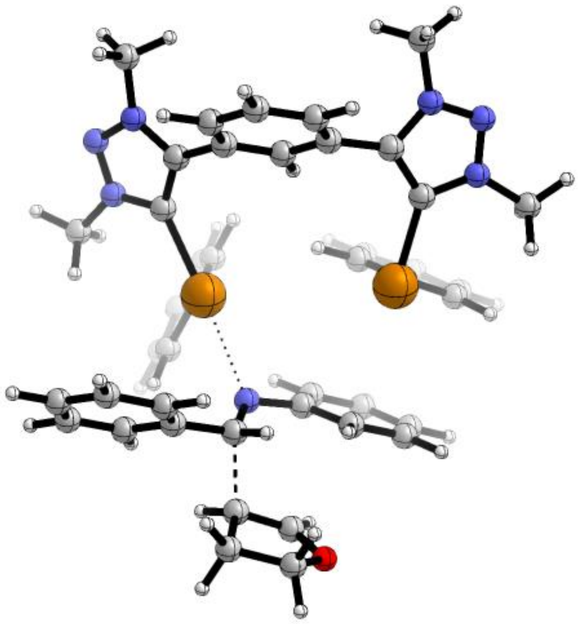

Figure S49: Transition state (for reaction proceeding via **TS**) according to DFT calculations.

C	3.87381200	-1.82407400	-2.77070300
C	3.57351800	-1.18326200	-1.56946900
C	2.70349600	-1.77769000	-0.65943300
C	2.15225200	-3.02533800	-0.94324000
C	2.45862400	-3.65651400	-2.14840000
C	3.31412800	-3.05759700	-3.05945800
C	1.23680400	-3.67540500	0.00859300
C	4.20551400	0.10857300	-1.25734600
C	0.05791700	-3.22815800	0.57647800
N	-0.37018400	-4.30715500	1.29101400
N	0.43378400	-5.33272400	1.22760000
N	1.40225500	-4.95573500	0.44786800
N	5.55752500	0.27129000	-1.21689400
N	5.89828300	1.49096800	-0.91962000
N	4.77887900	2.13445900	-0.77497200
C	3.67810900	1.35055700	-0.96124500
C	-1.60551600	-4.44073200	2.06058500
C	4.83022100	3.54899300	-0.41207000
C	6.61086600	-0.71832500	-1.44169900
C	2.48688700	-5.89579700	0.17393100
Te	-1.04744200	-1.37298600	0.19137500
Te	1.65845900	2.02105600	-0.86601500
C	1.76802300	2.38045800	1.21172400
C	1.50328100	1.34185800	2.09804500
C	1.56470000	1.57168500	3.46546200
C	1.88375600	2.83511200	3.94496400
C	2.12811000	3.87587400	3.05888600
C	2.06420700	3.65297000	1.68956300
C	-1.47896900	-1.26169000	2.26010400
C	-0.46465300	-1.39623500	3.20287700
C	-0.76850100	-1.38742400	4.55955200

C	-2.08221500	-1.22669800	4.97837300
C	-3.09302200	-1.08032300	4.03658800
C	-2.79490400	-1.10413500	2.68049500
H	4.52921700	-1.34832600	-3.48981600
H	2.46622900	-1.27815400	0.27235400
H	2.00638900	-4.61113100	-2.38682400
H	3.53509900	-3.54685000	-3.99810400
H	-1.77260600	-5.50017100	2.22612700
H	-1.50765400	-3.91054200	3.00555700
H	-2.41309700	-4.00398900	1.47656300
H	5.83052000	3.90679800	-0.63394100
H	4.08526100	4.07976200	-0.99970600
H	4.61088300	3.64964500	0.64869800
H	6.27505500	-1.67472300	-1.05124400
H	6.81833200	-0.79871500	-2.50621600
H	7.49600800	-0.37649800	-0.91439900
H	3.40626900	-5.33381900	0.03717900
H	2.56881100	-6.56074300	1.02809000
H	2.26053500	-6.46994300	-0.72177900
H	1.21433700	0.36851200	1.71994900
H	1.34703900	0.76654300	4.15525600
H	1.93109900	3.01290900	5.01130500
H	2.35820700	4.86531700	3.43218500
H	2.22143400	4.47399000	1.00091700
H	0.56542500	-1.52399900	2.88825900
H	0.02305500	-1.50357800	5.28923900
H	-2.31805500	-1.21656700	6.03405400
H	-4.11892900	-0.95614700	4.35792700
H	-3.58220100	-0.98835500	1.94767200
C	-3.10501500	0.81102500	-1.32375100
H	-2.62610000	1.32892700	-2.16156100
N	-2.47842000	0.78815900	-0.11169300
C	-1.89914100	1.96857800	0.30629800
C	-1.73143400	2.18121700	1.69389300
C	-1.56841500	3.04025700	-0.54773600
C	-1.33154500	3.40295700	2.18692700
H	-1.97949600	1.37455100	2.37065300
C	-1.14336500	4.27058700	-0.03776700
H	-1.63572300	2.92882100	-1.62264800
C	-1.04361000	4.46623300	1.32473300
H	-1.25022300	3.54251200	3.25759900
H	-0.92037100	5.07825700	-0.72411100
H	-0.74495800	5.42690400	1.72232200
C	-3.65850000	-0.50271700	-1.77989100
C	-3.29913300	-1.01049800	-3.02220300
C	-4.50038900	-1.24897300	-0.95929100
C	-3.75323200	-2.25902000	-3.43263000
H	-2.65068300	-0.43173200	-3.67102600
C	-4.96351700	-2.48776300	-1.36845800
H	-4.79056800	-0.85132900	0.00610100
C	-4.58489600	-2.99863300	-2.60620800

H	-3.46673200	-2.64569700	-4.40173400
H	-5.62892500	-3.05457300	-0.73026500
H	-4.95269300	-3.96340000	-2.92954100
C	-4.66725800	3.38669500	-3.07074400
H	-3.74846800	3.29613100	-3.65021600
C	-5.22652800	2.03815600	-2.61679000
H	-6.31442300	2.05920000	-2.55936900
C	-4.28126000	3.20748000	-0.86404300
H	-4.07195300	3.60855300	0.11865500
C	-4.61689000	1.88890500	-1.22356800
H	-5.14681700	1.31769200	-0.47297700
O	-4.31520000	4.07434700	-1.83308600
H	-5.36772200	4.02593200	-3.59917100
H	-4.94082000	1.23176600	-3.29070000

<u>Starting complex (TS)</u>

$\Delta E = -2658.150852$ ht

$\Delta G = -2657.46029$ ht

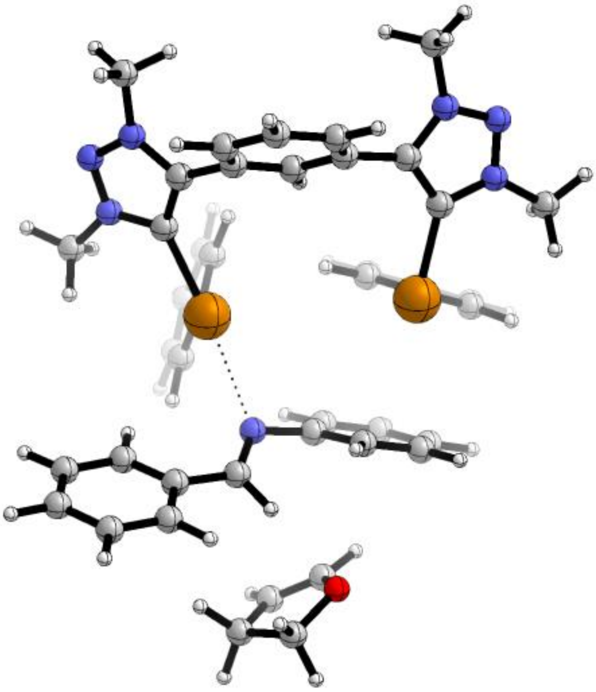

Figure S50: Starting complex (for reaction proceeding via **TS**) according to DFT calculations.

C	-4.31490000	1.35896600	-2.78186900
C	-3.91471900	0.77665800	-1.58018400
C	-3.09851900	1.48302400	-0.70100500
C	-2.70700400	2.78235100	-1.01533900
C	-3.11066700	3.35518100	-2.22074400
C	-3.90874600	2.64409400	-3.10261800
C	-1.87149000	3.55378500	-0.08088400

C	-4.38343600	-0.57454900	-1.23151200
C	-0.64084800	3.27462900	0.48114900
N	-0.36754200	4.38802300	1.21973200
N	-1.30883600	5.28649800	1.17053900
N	-2.21408500	4.79029400	0.38057400
N	-5.70481400	-0.88756400	-1.12351900
N	-5.89029100	-2.13437800	-0.80364500
N	-4.69939400	-2.64667500	-0.71316500
C	-3.70487500	-1.74554900	-0.95701400
C	0.82617200	4.66595000	2.01637900
C	-4.57086300	-4.05649100	-0.35148400
C	-6.87181800	-0.02481400	-1.30545400
C	-3.41998200	5.57362300	0.11559700
Te	0.72085300	1.64208700	0.13135100
Te	-1.61976900	-2.17648600	-1.01520900
C	-1.51040900	-2.66985900	1.03467700
C	-1.24714400	-1.66815400	1.96175800
C	-1.12915500	-1.98833400	3.30812000
C	-1.27549800	-3.30403700	3.72479800
C	-1.52467700	-4.30660500	2.79583300
C	-1.63299400	-3.99357900	1.44803000
C	0.97188500	1.40545500	2.22425400
C	-0.13003700	1.49866500	3.07113000
C	0.04145600	1.43220700	4.44825900
C	1.30882200	1.24732000	4.98524800
C	2.40329600	1.13071700	4.13968100
C	2.24011100	1.21749700	2.76204600
H	-4.92765400	0.79870400	-3.47736600
H	-2.78235400	1.02843600	0.23081400
H	-2.78109500	4.35317400	-2.48189800
H	-4.20584000	3.08768600	-4.04293100
H	0.83171000	5.72886900	2.23500100
H	0.79418000	4.07985000	2.93269700
H	1.69714500	4.38316000	1.42844800
H	-5.54165400	-4.51999400	-0.49491800
H	-3.82330900	-4.50783600	-0.99973200
H	-4.25771700	-4.12826100	0.68776100
H	-6.63375800	0.96375000	-0.92321800
H	-7.12562800	0.03122600	-2.36151700
H	-7.69229500	-0.46555300	-0.74783300
H	-4.25710700	4.89155100	-0.00203200
H	-3.57684100	6.23020500	0.96573400
H	-3.28466400	6.16274800	-0.78864000
H	-1.10632200	-0.64669900	1.63100200
H	-0.90936300	-1.20789000	4.02528400
H	-1.18456200	-3.55264400	4.77405400
H	-1.62171200	-5.33514200	3.11834100
H	-1.78728900	-4.78247100	0.72205400
H	-1.12750700	1.63209900	2.66665900
H	-0.81850400	1.51812000	5.10047300
H	1.44223500	1.19407900	6.05740000

H	3.39367900	0.98377500	4.55046000
H	3.10155500	1.12121600	2.11470400
C	3.46961700	-0.18802600	-1.12254900
H	3.78709300	-1.07201300	-1.68469400
N	2.61302800	-0.31203500	-0.18233300
C	2.15046800	-1.62868000	0.08384300
C	2.12489400	-2.07129000	1.40372800
C	1.77615800	-2.49772300	-0.94226900
C	1.80373100	-3.39048100	1.68718000
H	2.40501000	-1.38937800	2.19551400
C	1.44802100	-3.81629800	-0.65077400
H	1.79001900	-2.15317300	-1.96942800
C	1.47898800	-4.26967800	0.66231100
H	1.81835300	-3.73527900	2.71292000
H	1.19887600	-4.49481000	-1.45688200
H	1.24681800	-5.30260100	0.88586400
C	4.09641100	1.07139500	-1.52783000
C	4.62889800	1.14827200	-2.81561000
C	4.23113300	2.16149500	-0.66694000
C	5.24888000	2.30809600	-3.25248700
H	4.55011500	0.29232000	-3.47618000
C	4.86858500	3.31206300	-1.09640000
H	3.86306600	2.08809500	0.34691000
C	5.36936400	3.39034500	-2.39178600
H	5.65153600	2.36268900	-4.25480700
H	4.99537200	4.14622900	-0.41889200
H	5.87160000	4.28987600	-2.72251000
C	6.02286700	-2.55567700	-2.36028900
H	5.73354200	-1.97270800	-3.23290800
C	6.66374700	-1.73456200	-1.23052400
H	7.74575300	-1.85689500	-1.21454500
C	4.98101400	-3.10079600	-0.44424000
H	4.25973700	-3.67736700	0.11718700
C	5.97971900	-2.33700600	-0.02548600
H	6.25393600	-2.16921400	1.00287900
O	4.82858100	-3.14782900	-1.80304500
H	6.67553100	-3.37037800	-2.67750900
H	6.45811800	-0.66286700	-1.31838400

<u>Product complex (TS)</u>

ΔE = -2658.162044 ht

ΔG = -2657.461129 ht

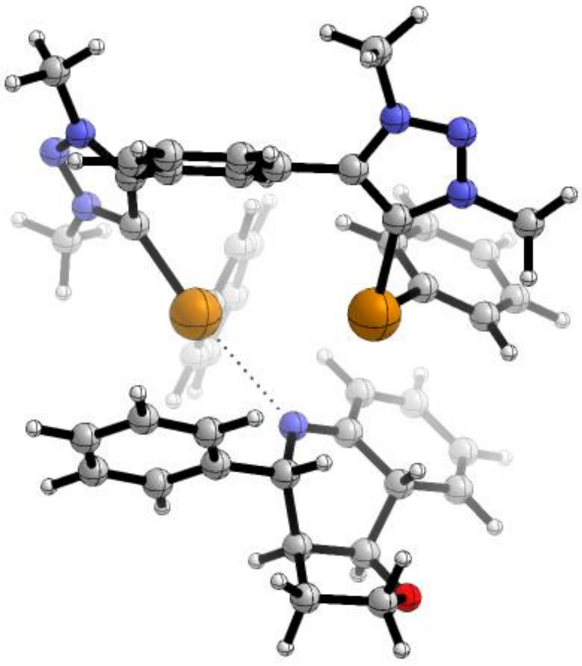

Figure S51: Product complex (for reaction proceeding via **TS**) according to DFT calculations.

C	-4.08196700	-2.36077500	-1.87430900
C	-3.23599700	-2.21035200	-0.77648300
C	-3.15771000	-0.98410900	-0.12118200
C	-3.92915700	0.09001500	-0.55964500
C	-4.76701800	-0.07039400	-1.66307800
C	-4.84573300	-1.29154500	-2.31396100
C	-3.84063000	1.39457900	0.11758400
C	-2.42110200	-3.34498000	-0.31132200
C	-2.74139100	2.17729800	0.42493600
N	-3.28868500	3.29533600	0.97590000
N	-4.59014300	3.25324300	1.04822100
N	-4.92523200	2.11203500	0.52439900
N	-2.94351900	-4.55137500	0.04374100
N	-2.02703200	-5.39181400	0.42843700
N	-0.90200700	-4.75138200	0.31507600
C	-1.05724700	-3.47445800	-0.13297700
C	-2.60104800	4.49579800	1.44883100
C	0.33919300	-5.42616200	0.68968600
C	-4.34227500	-4.98057500	0.05460600
C	-6.34363700	1.76130300	0.46359000
Te	-0.68203400	1.79458200	-0.04366300
Te	0.48470700	-2.07541200	-0.50737600
C	1.16773000	-2.08335200	1.48965700
C	0.43632400	-1.41702000	2.46941900
C	0.87681300	-1.43363700	3.78436300
C	2.04954900	-2.10149400	4.11653800
C	2.78879600	-2.74485800	3.13392700
C	2.35074400	-2.73841900	1.81529000
C	-0.01164200	2.62094500	1.77892900
C	-0.45648300	2.12373200	3.00081200

C	0.02327200	2.66952000	4.18487600
C	0.96260700	3.69289600	4.14962100
C	1.41279900	4.18006300	2.92975700
C	0.92090400	3.65223500	1.74242700
H	-4.12552700	-3.30560200	-2.40191200
H	-2.49983300	-0.86524700	0.73057400
H	-5.34269400	0.76912300	-2.03209900
H	-5.48964400	-1.40472900	-3.17525700
H	-3.34646300	5.27822900	1.54845200
H	-2.12232800	4.29009300	2.40377100
H	-1.84801100	4.76534400	0.71153600
H	0.11983600	-6.48493100	0.78271300
H	1.07417000	-5.24670500	-0.09182200
H	0.69355900	-5.01805200	1.63405100
H	-4.96169300	-4.12950900	0.32284900
H	-4.61845100	-5.35332500	-0.92910900
H	-4.43497500	-5.77175000	0.79214500
H	-6.43841800	0.68374500	0.56173400
H	-6.84100000	2.26571500	1.28621300
H	-6.76439000	2.09303600	-0.48298700
H	-0.46160500	-0.87361100	2.20123300
H	0.30997600	-0.91843900	4.54920900
H	2.39101100	-2.11431900	5.14334200
H	3.71106600	-3.25061400	3.38856300
H	2.93229300	-3.24005900	1.05107400
H	-1.17830200	1.31576000	3.03549400
H	-0.33114900	2.29135900	5.13523600
H	1.34110600	4.11138700	5.07263500
H	2.14382700	4.97723100	2.89980400
H	1.27112100	4.03744400	0.79275500
C	2.40901900	0.79956800	-1.92798400
H	2.36075700	-0.27762100	-2.15610600
N	2.14255200	0.99372000	-0.49672500
C	3.04255700	0.52098200	0.29625800
C	2.95643700	0.71343300	1.73285400
C	4.23030700	-0.22033200	-0.26029800
C	3.94114900	0.28575000	2.53903500
H	2.09391900	1.23238900	2.12641800
C	5.24396200	-0.66912600	0.73498300
H	3.84666100	-1.10710900	-0.79119100
C	5.11335600	-0.41603300	2.03784200
H	3.86992900	0.46857200	3.60459700
H	6.11419500	-1.17747100	0.33733600
H	5.87826400	-0.71954700	2.74030300
C	1.33785000	1.48058200	-2.74603500
C	0.36276100	0.73114500	-3.39376400
C	1.26781700	2.87005500	-2.81112900
C	-0.68096100	1.35225900	-4.07033100
H	0.42219400	-0.35186200	-3.37519900
C	0.23752500	3.49520200	-3.49595500
H	2.01523100	3.47231100	-2.30863700

C	-0.74662200	2.73757000	-4.11918100
H	-1.42774300	0.75488300	-4.57764500
H	0.20156800	4.57560300	-3.54618100
H	-1.54649900	3.22589400	-4.66013400
C	5.03710300	-0.48046100	-3.37840900
H	4.36707900	-1.33830700	-3.23203300
C	4.25505000	0.78705500	-3.69627100
H	4.91261500	1.51795900	-4.16441800
C	4.89189000	0.63602300	-1.37685100
H	5.52005400	1.38986800	-0.90123800
C	3.83008200	1.26631200	-2.30026200
H	3.86266600	2.35085500	-2.21937300
O	5.72376000	-0.17749500	-2.17231700
H	5.77901400	-0.74728600	-4.12725900
H	3.40612000	0.61667200	-4.35857300

Transitions state (invTS)

ΔE = -2658.112474 ht

ΔG = -2657.420504 ht

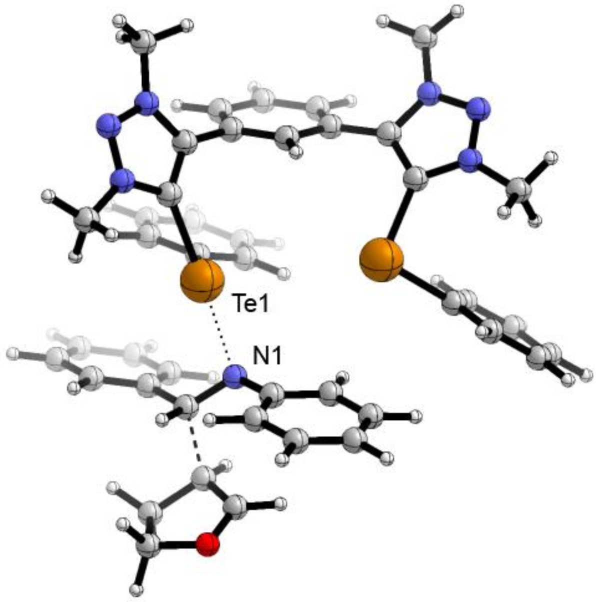

Figure S52: Transition state (for reaction proceeding via **invTS**) according to DFT calculations.

C	-1.09006000	4.15884200	0.97416900
C	-1.11566000	3.43551900	-0.21405500
C	-2.09648100	2.47010200	-0.41768700
C	-3.06296700	2.24224900	0.55770800
C	-3.02387900	2.96369300	1.75086600
C	-2.03908900	3.91607900	1.95629800
C	-4.11368300	1.25029700	0.30435500
C	-0.09259100	3.64602000	-1.24911900
C	-4.03367000	-0.05525000	-0.14629800

N	-5.33477800	-0.44864200	-0.23974900
N	-6.17642300	0.47757900	0.12014600
N	-5.44469700	1.50283600	0.44140500
N	0.07988300	4.81063300	-1.92915000
N	1.04935900	4.73420400	-2.79336400
N	1.51492800	3.52025600	-2.66424600
C	0.86599500	2.77598500	-1.72924200
C	-5.87110300	-1.73657800	-0.67633200
C	2.63820000	3.10478900	-3.49851200
C	-0.66123500	6.06165800	-1.79552900
C	-6.11074400	2.73397600	0.86499100
Te	-2.29901500	-1.21697200	-0.55830300
Te	1.42494100	0.75827300	-1.02410100
C	1.31653600	1.58831700	0.91916400
C	2.06550100	2.71262000	1.24585400
C	1.96507000	3.26578600	2.51612600
C	1.12813000	2.68991400	3.46286500
C	0.39391100	1.55621100	3.13785900
C	0.48285100	1.00464700	1.86567900
C	-3.09535900	-2.93004800	0.39161000
C	-3.44947300	-4.04337500	-0.36563500
C	-4.00085600	-5.15330800	0.26022500
C	-4.20996500	-5.14625000	1.63390900
C	-3.85334300	-4.03594400	2.38746200
C	-3.28548200	-2.92785000	1.77014000
H	-0.30621600	4.88548800	1.14730600
H	-2.11924700	1.92100700	-1.35232200
H	-3.74998800	2.76580800	2.52940800
H	-2.00075500	4.46095500	2.88955500
H	-6.91556900	-1.58170500	-0.92750000
H	-5.30552000	-2.06321800	-1.54559200
H	-5.76905100	-2.46372300	0.12656900
H	3.01627600	3.98514000	-4.00822500
H	3.39923300	2.66891100	-2.85503900
H	2.29732900	2.36611300	-4.22038900
H	-1.71007600	5.82717900	-1.63543800
H	-0.27225300	6.62876100	-0.95271700
H	-0.52984700	6.62260100	-2.71545200
H	-5.53111400	3.58015200	0.50701200
H	-7.10391800	2.73435800	0.42687700
H	-6.18231000	2.75843400	1.94988700
H	2.74897000	3.14577000	0.52467900
H	2.56376700	4.13061100	2.77323700
H	1.06182200	3.11355400	4.45673800
H	-0.24942900	1.09701000	3.87804400
H	-0.08184100	0.11482900	1.61482100
H	-3.30002200	-4.04591000	-1.43870000
H	-4.27384500	-6.02047500	-0.32674900
H	-4.64672400	-6.00971800	2.11781800
H	-4.00660400	-4.03421000	3.45858200
H	-2.99247400	-2.07008100	2.36304100

C	3.53467500	-1.33667200	0.18260100
H	4.08821700	-1.36798000	-0.76469600
N	2.16692200	-1.41004500	0.16375000
C	1.62168100	-2.37220800	-0.67863700
C	0.49494500	-3.08211000	-0.22195000
C	2.15453700	-2.73011500	-1.92580700
C	-0.03483200	-4.12768400	-0.95855800
H	0.11122800	-2.83972100	0.76359000
C	1.60039200	-3.76470200	-2.67405700
H	3.00521000	-2.19088300	-2.32588500
C	0.51352700	-4.47494700	-2.19374000
H	-0.85884700	-4.70098500	-0.55380400
H	2.03488400	-4.02263200	-3.63136200
H	0.10304500	-5.29813400	-2.76240800
C	4.08902900	-0.27909000	1.08509100
C	5.07342500	0.58878600	0.62708900
C	3.62854400	-0.16000900	2.39479900
C	5.59769000	1.56563600	1.46669100
H	5.43429600	0.49994600	-0.39221100
C	4.16049500	0.80068300	3.23721500
H	2.84475500	-0.82378500	2.74022000
C	5.14787200	1.66536700	2.77441300
H	6.36774600	2.23378400	1.10381100
H	3.80744600	0.87919800	4.25721200
H	5.56921000	2.41051100	3.43658100
C	6.05436800	-3.58635900	-0.43723000
H	6.16818300	-2.91698900	-1.28892200
C	5.75329600	-2.85836100	0.87234100
H	6.17466800	-3.38888900	1.72581800
C	3.90032700	-3.97586500	0.07651300
H	2.96978500	-4.52399100	0.00014100
C	4.22594500	-2.89704700	0.91820900
H	3.71603000	-2.85494600	1.87182100
O	4.85619400	-4.38553900	-0.69434900
H	6.89002000	-4.27847300	-0.40364400
H	6.15067100	-1.84521400	0.87544700

<u>Starting complex (invTS)</u>

ΔE = -2658.152374 ht

ΔG = -2657.466540 ht

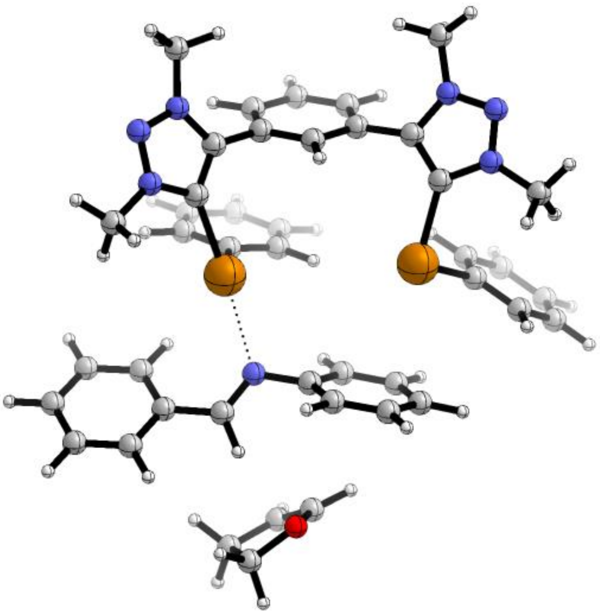

Figure S53: Starting complex (for reaction proceeding via **invTS**) according to DFT calculations.

C	1.77726800	-3.81774800	1.51182900
C	1.50943200	-3.41582200	0.20658100
C	2.29393300	-2.43678900	-0.39697300
C	3.36825300	-1.88773400	0.29586200
C	3.63231700	-2.29579500	1.60305300
C	2.83356600	-3.25183900	2.21003700
C	4.23779500	-0.89698500	-0.35702800
C	0.37978700	-3.99502000	-0.53679900
C	3.97818800	0.35444200	-0.87995800
N	5.19291100	0.77544600	-1.33629300
N	6.14325200	-0.08742900	-1.13412000
N	5.57087600	-1.09870900	-0.54937300
N	0.29303600	-5.30287900	-0.89875600
N	-0.80261800	-5.56101800	-1.55128600
N	-1.44030700	-4.42443700	-1.60333000
C	-0.77559400	-3.40134200	-1.00049900
C	5.52630200	2.05416800	-1.96136100
C	-2.74129400	-4.36963800	-2.26694800
C	1.24445800	-6.38217800	-0.64405800
C	6.38144800	-2.26309900	-0.19453200
Te	2.20819200	1.52934100	-0.89393900
Te	-1.54325100	-1.41670400	-0.67334400
C	-0.99014600	-1.60558300	1.35726700
C	-1.55487000	-2.60586900	2.14255700
C	-1.16600500	-2.74437500	3.46918100
C	-0.22961200	-1.87526400	4.01485200
C	0.32032700	-0.86947500	3.23169000
C	-0.05067100	-0.73610400	1.89886100
C	3.11204000	2.95120700	0.38553100
C	3.40627800	4.22485400	-0.09064300
C	3.95173900	5.17021100	0.76918000

C	4.20969700	4.84095700	2.09325800
C	3.92001500	3.56672300	2.56371000
C	3.36330700	2.62030100	1.71354400
H	1.14093200	-4.55002200	1.99353800
H	2.07851900	-2.12171400	-1.41116600
H	4.45249800	-1.85043100	2.15310500
H	3.02828200	-3.55087800	3.23083900
H	6.50381200	1.94737400	-2.42053900
H	4.76631500	2.28053100	-2.70515400
H	5.54015400	2.82818600	-1.19647400
H	-3.04897500	-5.38975800	-2.47279000
H	-3.44578900	-3.87517800	-1.60085200
H	-2.64976800	-3.80754300	-3.19366400
H	2.25086200	-5.99666000	-0.78266800
H	1.11955200	-6.74596000	0.37334400
H	1.03656800	-7.17663100	-1.35385100
H	5.79020100	-3.15905900	-0.36132400
H	7.25909300	-2.25624600	-0.83330600
H	6.67859500	-2.19841500	0.84962400
H	-2.30873100	-3.26660400	1.72979500
H	-1.61218300	-3.51704800	4.08196500
H	0.05768500	-1.97340800	5.05352600
H	1.04106000	-0.18351600	3.65903000
H	0.38967100	0.04164900	1.28641500
H	3.20833000	4.48335700	-1.12387400
H	4.17625000	6.16311200	0.40215200
H	4.63420400	5.57938400	2.76021300
H	4.11572300	3.31304100	3.59728800
H	3.11349000	1.63449900	2.08821800
C	-3.85273400	1.16531200	-0.21764000
H	-4.19075100	2.08804800	-0.70053400
N	-2.61901900	1.00326300	0.06436500
C	-1.73221600	2.06507000	-0.23328700
C	-0.95247100	2.58589600	0.79806700
C	-1.62841100	2.58880100	-1.51889700
C	-0.10912800	3.65828900	0.54958300
H	-1.05506900	2.17188700	1.79332600
C	-0.77221000	3.65336000	-1.76524400
H	-2.23848500	2.17864300	-2.31403900
C	-0.01668100	4.19537200	-0.73292600
H	0.47167700	4.08599700	1.35760300
H	-0.71286700	4.07266800	-2.76095700
H	0.62676100	5.04541300	-0.91856200
C	-4.87370700	0.15891900	0.07502400
C	-6.07452300	0.19383300	-0.63192100
C	-4.67971800	-0.80993300	1.06196200
C	-7.05758100	-0.75316200	-0.38719100
H	-6.23213600	0.96271100	-1.37983400
C	-5.66831600	-1.74382500	1.31595800
H	-3.76743500	-0.79395300	1.64567200
C	-6.85343100	-1.72195700	0.58569300

H	-7.98574800	-0.72689100	-0.94183000
H	-5.53156000	-2.47820500	2.09929600
H	-7.62805100	-2.44869000	0.79280100
C	-5.77217900	4.28866100	-0.90597700
H	-6.17124500	3.55073500	-1.59970900
C	-5.96178400	3.94061400	0.57856800
H	-6.78729100	4.49975000	1.01632300
C	-3.78945000	4.55606500	0.11584900
H	-2.74551400	4.83517800	0.11016200
C	-4.61268300	4.33803100	1.13128500
H	-4.35934800	4.41710500	2.17546000
O	-4.34639900	4.37619900	-1.12036000
H	-6.20006400	5.26465600	-1.14036100
H	-6.16628900	2.87716200	0.74232700

Product complex (invTS)

ΔE = -2658.163986 ht

ΔG = -2657.465893 ht

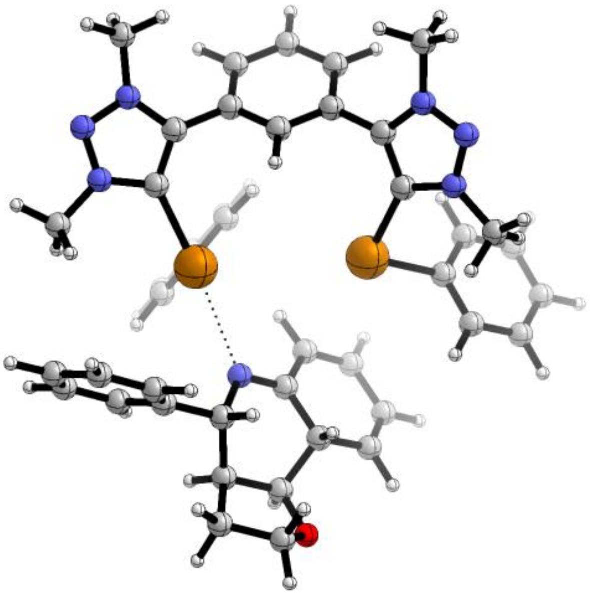

Figure S54: Product complex (for reaction proceeding via **invTS**) according to DFT calculations.

C	2.35838000	-4.36531500	1.02476700
C	1.53436700	-3.74380800	0.08766500
C	2.03313600	-2.68498900	-0.66889700
C	3.34838200	-2.26583500	-0.49594900
C	4.17020900	-2.90672600	0.43001500
C	3.67225100	-3.95319700	1.18904900
C	3.85208200	-1.11608500	-1.26045600

C	0.13844900	-4.17063700	-0.08076500
C	3.41351500	0.19288200	-1.29369000
N	4.27342300	0.80248100	-2.15664600
N	5.17964000	-0.00335800	-2.63051500
N	4.92441300	-1.16117100	-2.09585500
N	-0.24636400	-5.47607000	-0.16496500
N	-1.53753900	-5.59807700	-0.28017900
N	-1.99783800	-4.38334300	-0.26923500
C	-1.02833600	-3.43225400	-0.15397300
C	4.30326700	2.20733700	-2.55937400
C	-3.44482800	-4.17800700	-0.35727900
C	0.56563400	-6.69281000	-0.18022800
C	5.76981600	-2.30503100	-2.43581200
Te	1.83321500	1.10000000	-0.19557300
Te	-1.50236500	-1.31957500	-0.10899300
C	-1.25612700	-1.27861700	1.98865900
C	-2.35829200	-1.00474000	2.79006200
C	-2.20084800	-0.91654600	4.16762900
C	-0.95167200	-1.11102800	4.74076100
C	0.14665100	-1.38962200	3.93721600
C	0.00027200	-1.46583100	2.55819400
C	3.08237900	2.67448100	0.46184700
C	2.82420100	3.97603700	0.04350200
C	3.64953800	5.00965700	0.46845800
C	4.73367000	4.74134700	1.29348300
C	4.98823100	3.44050500	1.70970000
C	4.15741900	2.40425600	1.30423000
H	1.96809800	-5.15752200	1.65055400
H	1.39900700	-2.19578300	-1.39786400
H	5.18809000	-2.56791200	0.57894400
H	4.30172700	-4.43677700	1.92338000
H	4.88793900	2.27306900	-3.47143100
H	3.27964000	2.53438500	-2.72572200
H	4.75485800	2.79823400	-1.76470700
H	-3.90398000	-5.15712800	-0.44863200
H	-3.78087200	-3.67062000	0.54440400
H	-3.66753400	-3.55474100	-1.22014300

H	1.52076200	-6.46744300	-0.64565100
H	0.71385600	-7.05078600	0.83607900
H	0.02592900	-7.43665600	-0.75815300
H	5.14604700	-3.19363200	-2.47680700
H	6.21843800	-2.10524200	-3.40390400
H	6.54518400	-2.42334600	-1.68224200
H	-3.33102500	-0.84820500	2.34130300
H	-3.05679400	-0.69844300	4.79258500
H	-0.83320900	-1.04313800	5.81398400
H	1.12198800	-1.53507400	4.38347800
H	0.86648700	-1.64580800	1.93192700
H	1.98514500	4.18823200	-0.60722400
H	3.44742500	6.02332200	0.14780400
H	5.37872100	5.54729600	1.61723800
H	5.82673100	3.23335100	2.36148300
H	4.34366700	1.39510000	1.65083000
C	-3.28852800	1.76269000	-0.83230800
H	-2.75496600	2.04837700	-1.75128600
N	-2.29209000	1.40482400	0.18303900
C	-1.57796400	2.37948400	0.61912900
C	-0.59326200	2.17211300	1.67114100
C	-1.75082400	3.76543400	0.05897400
C	0.12873300	3.20442300	2.14601200
H	-0.50594500	1.17999700	2.09564400
C	-0.90959200	4.82655700	0.67880800
H	-1.50449100	3.71137300	-1.01314300
C	-0.03314500	4.56265400	1.64978300
H	0.83715700	3.03293600	2.94852600
H	-1.06602400	5.83482700	0.31350000
H	0.55582500	5.35128400	2.09848800
C	-4.16490900	0.57551700	-1.15045400
C	-4.19451300	0.02754600	-2.42540700
C	-4.94190500	-0.00294600	-0.14943100
C	-4.97997000	-1.08758200	-2.70065600
H	-3.60026500	0.47464000	-3.21476200
C	-5.72808000	-1.11227100	-0.41690700
H	-4.92782200	0.41973800	0.84897800

C	-5.74815600	-1.65864900	-1.69667600
H	-5.00615500	-1.49471500	-3.70325100
H	-6.34275500	-1.54053200	0.36491400
H	-6.38013200	-2.51058300	-1.91391300
C	-4.04663600	4.73723200	-1.98619100
H	-3.25392300	4.38891100	-2.66099400
C	-4.94057300	3.58883900	-1.53840600
H	-5.88770600	3.98094600	-1.17104400
C	-3.25485800	4.15098900	0.08752200
H	-3.50894400	4.47201900	1.09834500
C	-4.13379200	2.97588400	-0.38387900
H	-4.78203500	2.64411800	0.42530100
O	-3.48508700	5.23719800	-0.77955500
H	-4.57732500	5.55344600	-2.47030400
H	-5.15245200	2.87179900	-2.33134900

References

[1] S. G. Alvarez, M. T. Alvarez, *Synthesis* **1997**, *1997*, 413-414.

[2] F. Kniep, L. Rout, S. M. Walter, H. K. V. Bensch, S. H. Jungbauer, E. Herdtweck, S. M. Huber, *Chem. Commun.* **2012**, *48*, 9299–9301.

[3] P. Wonner, A. Dreger, L. Vogel, E. Engelage, S. M. Huber, *Angew. Chem. Int. Ed.* **2019**, *58*, 16923-16927.

[4] T. Steinke, P. Wonner, E. Engelage, S. M. Huber, *Synthesis* **2021**, *53*, 2043-2050.

[5] P. Wonner, T. Steinke, L. Vogel, S. M. Huber, *Chem. Eur. J.* **2020**, *26*, 1258-1262.

[6] J. Ścianowski, A. J. Pacuła, A. Wojtczak, *Tetrahedron: Asymmetry* **2015**, *26*, 400-403.

[7] a) H. Xu, S. J. Zuend, M. G. Woll, Y. Tao, E. N. Jacobsen, *Science* **2010**, *327*, 986-990; b) X. Liu, P. H. Toy, *Adv. Synth. Catal.* **2020**, *362*, 3437-3441; c) Z. Tang, F. Jiang, L.-T. Yu, X. Cui, L.-Z. Gong, A.-Q. Mi, Y.-Z. Jiang, Y.-D. Wu, *J. Am. Chem. Soc.* **2003**, *125*, 5262–5263; d) G. Sundararajan, N. Prabagaran, B. Varghese, *Org. Lett.* **2001**, *3*, 1973-1976.

[8] a) J. Burés, *Angew. Chem. Int. Ed.* **2016**, *55*, 16084-16087; b) C. D. T. Nielsen, J. Burés, *Chem. Sci.* **2019**, *10*, 348-353.

[9] Y. Zhao, D. G. Truhlar, *Theor. Chem. Acc.* **2008**, *120*, 215-241.

[10] S. Grimme, *Chem. Eur. J.* **2012**, *18*, 9955-9964.

[11] a) F. Weigend, R. Ahlrichs, *Phys. Chem. Chem. Phys.* **2005**, *7*, 3297–3305; b) D. Rappoport, F. Furche, *J. Chem. Phys.* **2010**, *133*, 134105; c) E. Engelage, D. Reinhard, S. M. Huber, *Chem. Eur. J.* **2020**, *26*, 3843-3861.

[12] R. C. Gaussian 16, G. W. T. M. J. Frisch, H. B. Schlegel, G. E. Scuseria, , J. R. C. M. A. Robb, G. Scalmani, V. Barone, , H. N. G. A. Petersson, X. Li, M. Caricato, A. V. Marenich, , B. G. J. J. Bloino, R. Gomperts, B. Mennucci, H. P. Hratchian, , A. F. I. J. V. Ortiz, J. L. Sonnenberg, D. Williams-Young, , F. L. F. Ding, F. Egidi, J. Goings, B. Peng, A. Petrone, , D. R. T. Henderson, V. G. Zakrzewski, J. Gao, N. Rega, , W. L. G. Zheng, M. Hada, M. Ehara, K. Toyota, R. Fukuda, , M. I. J. Hasegawa, T. Nakajima, Y. Honda, O. Kitao, H. Nakai, , K. T. T. Vreven, J. A. Montgomery, Jr., J. E. Peralta, , M. J. B. F. Ogliaro, J. J. Heyd, E. N. Brothers, K. N. Kudin, , T. A. K. V. N. Staroverov, R. Kobayashi, J. Normand, , A. P. R. K. Raghavachari, J. C. Burant, S. S. Iyengar, , M. C. J. Tomasi, J. M. Millam, M. Klene, C. Adamo, R. Cammi, , R. L. M. J. W.

Ochterski, K. Morokuma, O. Farkas, , a. D. J. F. J. B. Foresman, Gaussian, Inc., Wallingford CT, 2019., **2019**.
[13] C. Legault C. Y., 1.0b, Université de Sherbrooke, 2009, http://www.cylview.org.
